# Risk identification method for automotive styling design tasks based on an improved FAHP-VIKOR approach

**DOI:** 10.1371/journal.pone.0352278

**Published:** 2026-07-06

**Authors:** Jiefeng Lyu, Ganshu Sheng, Zihao Wang

**Affiliations:** 1 School of Art and Design, Wuhan University of Technology, Hubei, Wuhan, China; 2 School of Industrial Design, Hubei University of Technology, Hubei, Wuhan, China; Institute of Infrastructure Technology Research and Management, INDIA

## Abstract

To address the difficulty of quantifying risks in current automotive styling-design task management, this study proposes a risk identification method for automotive styling design tasks based on an improved FAHP-VIKOR approach. First, the work breakdown structure (WBS) is constructed based on the principle of task modularity, and the risk breakdown structure (RBS) is established through the analysis of factors influencing risk, thereby forming the WBS-RBS coupling matrix for automotive styling design. Then, the improved fuzzy analytic hierarchy process (FAHP) is employed to determine the weights of risk factors such as technical, schedule, and cost risks, while the VlseKriterijumska Optimizacija I Kompromisno Resenje (VIKOR) method is applied to perform comprehensive ranking that adapts to the multidimensional and dynamic characteristics of styling design risks, thereby enabling quantitative risk assessment and identification. Finally, the design tasks involved in the exterior styling concept-design phase of a certain sport utility vehicle (SUV) model are taken as a case study and compared with the single FAHP method, the FAHP-TOPSIS method and the FAHP-VIKOR method. The results indicate that the proposed approach can classify the 27 core design tasks into high, medium and low risks, and can effectively identifies high-risk nodes such as the front bumper surface, lidar surface, and rear bumper surface. Compared with the other methods, the proposed method produces more stable and reasonable risk identification results and can provide decision-making support for risk management and optimal resource allocation in automotive styling design projects.

## 1 Introduction

The global automotive industry is experiencing a transformation characterized by the “new four modernizations,” through which automobiles are shifting from mechanical tools to intelligent terminals. As the key interface connecting brand, users, and technology, automotive styling design has gained unprecedented strategic significance. Successful styling design can shape brand recognition, lead aesthetic trends, and directly influence commercial performance [1]. However, automotive styling design projects are complex activities that combine artistic creativity with engineering rigor [2]. Their processes cover multiple key tasks, including market aesthetic research, styling concept generation, digital and clay model development, as well as engineering feasibility verification, cost control, and mass production process matching [3]. As a result, such projects are typically characterized by long development cycles, high investment, and strong interdependencies among different stages. Numerous risks emerge throughout the design process, such as conflicts between creative styling and engineering feasibility, mismatches between design schemes and user preferences, and failures in new technology integration. If these risks are not identified or managed in time, they may cause repeated revisions, schedule delays, or even unfavorable market performance after vehicle launch, potentially resulting in economic loss and wasted research and development resources. Therefore, systematically and scientifically identifying potential risks in automotive styling design tasks is crucial for enhancing design-management quality and project success rates.

Traditional risk management approaches mainly include the Delphi method [4], brainstorming techniques [5], and fault tree analysis (FTA) [6]. These methods rely heavily on expert knowledge and experience to identify risk sources through qualitative judgment. However, they lack systematic identification frameworks and quantitative evaluation approaches, making them difficult to apply to the complex and highly interconnected risk structures found in modern automotive styling design projects.

In recent years, with the increasing digitalization and parallelization of vehicle development processes, researchers have begun to focus on risk identification within automotive product development projects. Yousefi et al. [7] applied a robust data envelopment analysis-failure mode and effects analysis (DEA-FMEA) method to prioritize health, safety, and environment risks in automotive component manufacturing. Li et al. [8] conducted risk assessment for automotive component projects using the analytic hierarchy process (AHP). Mzougui et al. [9] assessed risks in the automotive supply chain using a modified failure mode, effects and criticality analysis approach based on multi-criteria decision making (MCDM). Hu et al. [10] used Bayesian networks to assess automotive supply chain risks. Li et al. [11] applied fuzzy analytic hierarchy process (FAHP) to construct a hierarchical outcome model and evaluated risks in automotive styling design projects from a project management perspective. Ju et al. [12] integrated AHP and the entropy weight method to quantify evaluation results from both subjective and objective dimensions and thereby select the optimal styling scheme, offering an alternative perspective for risk identification in automotive styling.

In the broader fields of design and engineering, researchers have attempted to systematize risk analysis through methods such as risk matrix analysis [13], fuzzy comprehensive evaluation (FCE), analytic hierarchy process (AHP) [14] and multi-criteria decision making (MCDM) [15]. Zhang et al. [16] applied the work breakdown structure-risk breakdown structure (WBS-RBS) method to identify risks among automotive component suppliers. Wu et al. [17] used the WBS–RBS method to identify risks in tunnel and foundation pit construction and applied FCE for risk evaluation. Zhou et al. [18] proposed a risk identification method for metro foundation pits based on fault tree analysis combined with WBS-RBS. Xiang et al. [19] calculated the risk level of bridge design using an AHP-FCE model. Lei et al. [20] integrated AHP with a cloud model to assess risks in highway survey and design. Hyun et al. [21] combined AHP with FTA to identify risks in shield tunnel construction. Pour et al. [22] adopted FAHP to identify risks in natural gas and petroleum drilling processes. Chen et al. [23] constructed an evaluation model using WBS-RBS and AHP to identify risks in cross-sea bridge engineering. Dong et al. [24] analyzed the risk factors associated with immersed tunnel construction using the WBS–RBS framework and calculated risk weights through an improved AHP enhanced by a genetic algorithm. Chen et al. [25] applied WBS-RBS and an improved FAHP method to identify risks in information system construction projects within coal enterprises. Ji et al. [26] developed evaluation indicators for bridge construction using WBS-RBS and conducted risk identification through Delphi-modified FAHP.

It is evident that current risk identification research for automotive products predominantly focuses on the production and manufacturing stages of automotive components or the procurement and supply chain phases, while studies specifically dedicated to risk identification within vehicle styling design tasks themselves remain scarce. Methods such as WBS-RBS, AHP, and their combinations have been applied in tunneling, bridge construction, and large-scale engineering projects. However, single risk assessment methods such as WBS-RBS and AHP have difficulty fully capturing the fuzziness and uncertainty inherent in expert judgments, The weight-determination process often lacks stability and cannot adequately describe the complex relationships between risks and design tasks in automotive styling design projects. WBS-RBS emphasizes risk identification but not risk evaluation, while AHP suffers from subjectivity in indicator design and judgment-matrix construction, together with challenges in consistency testing. The method combining WBS-RBS and AHP generally adopts a single subjective scoring approach, which involves strong subjectivity. Moreover, for complex automotive styling design projects, conducting the consistency test of the judgment matrix becomes extremely difficult.

At the risk-ranking stage, fully compensatory methods are commonly adopted, which makes it difficult to reflect the dominant influence of critical risk factors on overall decision-making. Therefore, most studies on risk assessment and multi-attribute decision-making in complex engineering systems have introduced MCDM methods to achieve comprehensive ranking of risk nodes. For instance, Han et al. [27] integrated random forest, the VlseKriterijumska Optimizacija I Kompromisno Resenje (VIKOR) method, and geographic information systems (GIS) to identify risks of coal mine floor water inrush. Jianxing et al. [28] improved the FMEA approach by incorporating a cloud model and an extended VIKOR method for risk assessment of subsea pipelines. Wang et al. [29] employed FAHP to determine the weights of risk factors and applied the VIKOR method to evaluate the risks of existing buildings adjacent to tunnel excavation. Liu et al. [30] integrated clustering algorithms into the AHP–VIKOR framework to assess geological disaster risks induced by extreme rainfall. Yadav et al. [31] proposed a hybrid method based on Technique for Order Preference by Similarity to Ideal Solution (TOPSIS) and PSI, which constructs comprehensive evaluation indicators and ranks alternatives to achieve optimal selection of multi-performance materials. Gangwar et al. [32] combined AHP and multi objective optimization on the basis of ratio analysis (MOORA) methods to perform comprehensive evaluation and optimal selection of materials under multiple performance criteria. Lyu et al. [33] integrated MCDM with GIS techniques to assess flood risks in metro systems. Mir et al. [34] applied the Preference Ranking Organization Method for Enrichment Evaluation II (PROMETHEE II) method to conduct disaster risk assessment for mountainous geographical education infrastructure. Tran et al. [35] proposed an effective MCDM model by integrating FAHP and TOPSIS to evaluate and select the optimal robot.

It can be observed that MCDM methods such as VIKOR, TOPSIS, PROMETHEE, and MOORA have been widely applied to risk ranking across various engineering domains. These methods enable structured comparisons under conditions of multidimensional criteria conflicts and help mitigate biases introduced by single compensatory evaluation approaches. However, TOPSIS is based on a fully compensatory assumption, whereby the disadvantage of one criterion can be offset by the advantages of others, which may weaken the influence of veto type critical risks such as regulatory compliance and engineering feasibility. PROMETHEE relies on the specification of preference functions and threshold parameters, making its results sensitive to parameter selection and thus difficult to ensure stability and reproducibility in highly subjective design evaluation contexts. Similarly, MOORA is also grounded in a fully compensatory framework and lacks explicit modeling of interactions among criteria, making it difficult to capture the decisive impact of extreme risk factors. In contrast, the VIKOR method is based on the concept of a compromise solution. By balancing group utility and individual regret, it effectively highlights the dominant role of critical risks and is particularly suitable for automotive styling design projects characterized by constraint-driven risk features.

Overall, existing studies on automotive product risk identification have primarily focused on the production and manufacturing stages of components or the procurement and supply chain phases, while relatively few have addressed risk identification specifically for vehicle styling design tasks. This stage is characterized by high uncertainty, multi-factor coupling, and strong subjectivity. When applied to styling design tasks, existing methods exhibit several limitations: traditional approaches tend to rely on qualitative analysis and lack structured and quantitative capabilities; AHP and its extensions are highly subjective and struggle to capture fuzziness; WBS-RBS can effectively identify risks but lacks ranking capability; and fully compensatory methods such as TOPSIS fail to reflect the “non-compensability of critical risks” inherent in styling design. Therefore, significant research gaps remain in terms of developing systematic risk identification frameworks tailored to styling design tasks, quantitative ranking methods under multi-criteria conflicts, and mechanisms that capture the dominant role of critical risks. Although FAHP and VIKOR have demonstrated advantages in weight determination and multi-criteria decision-making, respectively, to the best of our knowledge, their integration has not yet been explored for risk identification in automotive styling design tasks.

Therefore, to effectively and objectively quantify the risk levels of automotive styling design tasks, this study integrates an improved FAHP with the VIKOR method to construct a two-stage evaluation framework consisting of fuzzy weight determination and compromise ranking. The former captures the fuzziness and subjective preferences inherent in expert judgments, whereas the latter determines a compromise solution under multiple conflicting risk criteria. This framework establishes a closed-loop process from risk identification to risk ranking, which facilitates the identification of high-risk design task nodes that may have decisive impacts on project success. Based on the risk influencing factors of automotive styling design tasks, the concepts of the WBS-RBS are introduced. By integrating the FAHP method with the VIKOR method, a risk identification approach for automotive styling design tasks is proposed. The proposed model simultaneously considers multidimensional risk factors, including technology, schedule, cost, and collaboration, thereby enhancing its applicability in complex engineering design environments and providing clear decision support for risk control in automotive styling design management.

This study makes two main contributions: (a) A WBS–RBS risk matrix tailored to the automotive styling design process was constructed, clearly depicting the coupling between tasks and associated risks. (b) An improved FAHP–VIKOR risk quantification framework was proposed to accurately classify and rank the risk levels of automotive styling design tasks. The novelty of this study lies in the organic integration of the improved FAHP with VIKOR: the improved FAHP precisely determines the weights of risk factors, while VIKOR, leveraging its compromise decision-making principle that balances group utility and individual regret, enables a risk ranking of design tasks that better reflects engineering practice. The determination of risk occurrence probability and loss severity in the risk matrix relies on expert experience scoring. Although multi-expert averaging and iterative discussion were adopted to reduce bias, a certain degree of subjectivity remains unavoidable.

## 2 Overview

**Fig 1:**
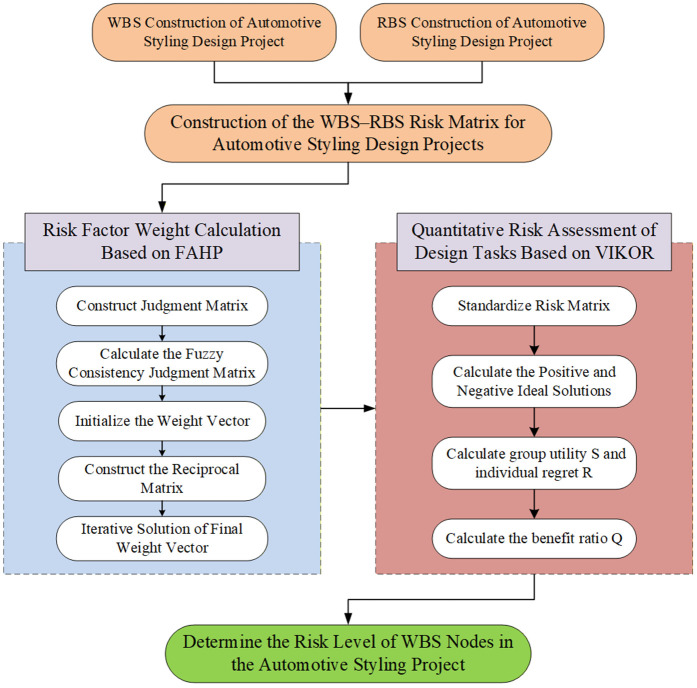
Risk Identification Process for Automotive Styling Design Tasks Based on FAHP-VIKOR.

The FAHP-VIKOR-based risk identification method for automotive styling design tasks consists of four major steps: construction of the WBS for styling design tasks, construction of the RBS for styling design tasks, construction of the WBS-RBS matrix, and quantification of design task risks based on FAHP-VIKOR, as illustrated in Fig 1.

(1) Construction of WBS for styling-design tasks: Based on automotive structural characteristics and principles of physical decomposition between units, a WBS is developed for each stage of styling design.
(2) Construction of RBS for styling-design tasks: Based on the international electrotechnical commission (IEC) risk assessment technical standards, the China automotive technology and research center (CATARC) styling process guidelines, and the enterprise level styling design process management system, adverse influencing factors of automotive styling design tasks are analyzed, and a set of general indicators for risk analysis is summarized.
(3) Construction of the WBS-RBS matrix: Work packages at the lowest WBS level and risk factors at the lowest RBS level serve as matrix rows and columns, respectively. The product of risk occurrence probability and loss severity is defined as the risk value of each matrix element.
(4) Quantification of design task risks: Improved FAHP-VIKOR is applied to calculate risk-factor weights for each WBS task and to evaluate comprehensive benefit ratios for identifying risk levels across styling-design tasks.

The structure of this paper is as follows: Sect 1 reviews existing studies on risk identification methods in engineering fields such as automotive, bridge, and geotechnical engineering. Sect 2 presents an overview of the risk identification framework for automotive styling design tasks. In Sect 3, a WBS-RBS risk matrix is constructed based on the WBS and RBS decomposition of automotive styling design tasks. Sect 4 proposes an improved FAHP-VIKOR method based on the WBS-RBS risk matrix to quantify the risk levels of automotive styling design tasks. Sect 5 presents a case study of an SUV exterior styling design project, including case analysis, comparative experiments, and corresponding management recommendations. Finally, conclusions and future research directions are provided in Sect 6.

## 3 Construction of the WBS-RBS matrix for automotive styling design

### 3.1 WBS of automotive styling design tasks

The WBS is a project-management tool that decomposes an automotive styling design project into manageable tasks. First, automotive styling design development is defined as the first level overall objective. Based on the general automotive styling design process and the project lifecycle theory [36], the automotive styling design work was divided into phases following the Initiation, Planning, Execution, Control, and Closing cycle. In combination with the CATARC styling guidelines and the phased design processes commonly adopted by major Chinese automakers such as Dongfeng, SAIC, and Geely during full-vehicle development, the overall objective was decomposed into five second level strategic stages: Project initiation and preparation, creative design and exploration, design refinement and digital model development, physical model verification and evaluation, final delivery and archiving. These stages cover the entire process from early conceptual exploration to final production support.

**Fig 2:**
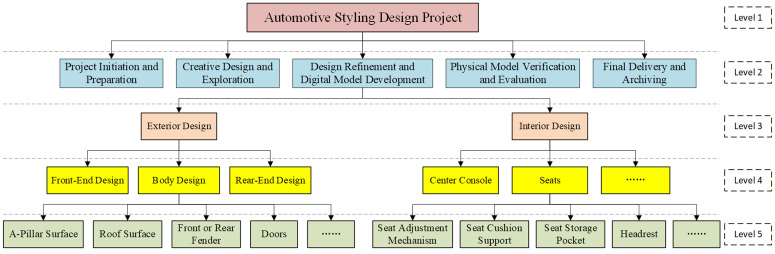
WBS Decomposition Diagram for Automotive Styling Design.

Within the creative design, digital surface development, and physical model evaluation stages, the WBS is constructed according to the physical structure of the automobile and the principle of physical modular decomposition among system units [37]. Automotive styling consists mainly of exterior and interior sections, each divided into multiple modules, and each module consists of several components. Together, they constitute the full WBS of styling design. For example, the exterior consists of three major modules-body, front fascia, and rear fascia. The body module includes the A-pillar surface, B-pillar surface, C-pillar surface, D-pillar surface, roof surface, windows, doors, front or rear fenders, wheels, etc. To ensure the rationality and industry applicability of the WBS structure, domain experts were invited to review and revise the preliminary structure after its initial construction (detailed information about the experts is proSect 3.3vided in Sect 3.3). In addition, the task hierarchy and module divisions were further verified by referencing the complete vehicle development process and relevant project experience, ultimately resulting in the WBS structure adopted in this study. The corresponding WBS structure is shown in Fig 2.

### 3.2 RBS of automotive styling-design tasks

The risks in automotive styling design mainly arise from the potential adverse impacts of styling decisions, including exterior appearance, structural layout, materials, proportions, and detailed design, on safety, functionality, human–machine interaction, manufacturability, and regulatory compliance [38]. Referring to the IEC 31010:2009 *Risk Management—Risk Assessment Techniques* standard as the overall risk management framework, and drawing on relevant international and domestic standards (including the GB/T 34590 series, ISO 21448, ISO 15005, and GB/T 43407), as well as the CATARC styling process guidelines and enterprise design processes used by major automotive manufacturers (such as Dongfeng, SAIC, and Geely), the risk factors throughout the automotive styling design process are classified into five dimensions: technical risk, schedule risk, cost risk, resource and collaboration risk, and external and compliance risk.

(1) Technical risk

During the transition from sketches to digital models, geometric distortion or inconsistency in form may occur. In addition, styling solutions may conflict with engineering structures, material processes, or regulatory requirements, leading to repeated design revisions or even complete redesign. For example, the stamping feasibility of key body surfaces (such as the hood and tailgate) must be verified, while aggressive styling features, such as a low-profile body or a large rear wing, may result in an excessively high aerodynamic drag coefficient.

(2) Schedule risk

Due to the highly parallel and iterative nature of automotive design projects, critical tasks are often affected by competition for resources or delays in decision-making, which compress the available time for design activities. For instance, repeated revisions of CAS data may delay its release, which in turn postpones clay model production, while disputes regarding Class-A surfaces may prevent design data from being delivered to engineering teams on schedule.

(3) Cost risk

Cost risk includes both explicit expenses, such as mold modifications and prototype remanufacturing caused by design changes, and implicit costs associated with material or process premiums. For example, the cost of special styling components, such as customized grilles or fastback tailgates, may exceed the planned budget, while color, material, finishing (CMF) schemes involving special paint processes or new environmentally friendly materials may also increase costs.

(4) Resource and Collaboration risk

The loss of key personnel may disrupt the continuity of design language, while cross-team collaboration may be hindered by unclear responsibilities, inefficient communication, or system failures, creating information barriers that obstruct task coordination. For example, the departure of a lead exterior designer may make it difficult to maintain the established front-end styling language, or the steeply inclined A-pillar preferred by the styling team may conflict with the field of view safety requirements of the engineering team.

(5) External and Compliance risk

Changes in the external environment are highly uncertain. Updates in domestic or international regulations may require mandatory design modifications, supply chain fluctuations may affect the availability of key components, and public opinion pressure may also lead to redesign. For instance, the hood shape may need to be modified to meet updated pedestrian protection regulations, or a large screen interior layout may require redesign if it fails to meet crash safety standards in the target market.

**Fig 3:**
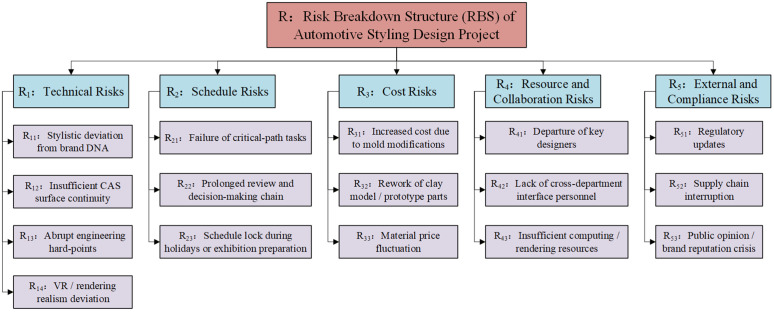
RBS Decomposition Diagram for Automotive Styling Design Project.

RBS decomposes risks into hierarchical categories and subcategories, enabling systematic identification and analysis. Based on the above analysis, a multi-level risk indicator system for automotive styling design is developed, as illustrated in Fig 3.

### 3.3 Construction of the WBS-RBS Matrix

The WBS-RBS method combines the WBS and RBS to identify potential risks through a cross analysis of project tasks and associated risk factors. The construction process consists of the following steps:

(1) Constructing the WBS-RBS matrix

All work packages in the WBS are arranged as the rows of the matrix, while all risk factors in the RBS serve as the columns. The intersections between rows and columns represent potential risk factors associated with each specific design task. The risk value of the j-th risk factor in the i-th styling design task is denoted as $x_{ij}$.

(2) Determination of the matrix cell risk value $x_{ij}$

Within automotive styling design projects, the probability that risks occur during design, production, or delivery processes, as well as the magnitude of the loss incurred, cannot be accurately quantified. Consequently, the abstract notion of risk must be transformed into specific, measurable numerical values. To this end, a cardinal fuzzy evaluation approach is adopted, whereby integers ranging from 0 to 9 are used to represent the severity level, with 0 indicating the lowest and 9 indicating the highest magnitude. According to system safety engineering theory and the Likelihood-Exposure-Consequence evaluation method [39], the magnitude of risk is related to both the probability of occurrence and the severity of potential losses. The basic formulation defines risk as the product of the probability of an accident and the magnitude of the resulting loss. Accordingly, the risk value $x_{ij}\;$is calculated as the following Eq (1).


$$\begin{array}{c} x_{ij}=P_{ij}\times L_{ij} \end{array}$$ (1)

where $x_{ij}$ denotes the degree of risk associated with risk factor *j* at WBS task node *i*; $P_{ij}\;$represents the probability of occurrence of risk factor *j* at task node *i*; and $L_{ij}\;$denotes the loss severity associated with risk factor j at task node *i*.

For each task node, the values of P and L are assigned by an expert panel in automotive styling design, based on collective discussion and professional judgement. The specific rating criteria for P and L are shown in Table 1.

**Table 1:** Rating criteria for risk occurrence probability and loss severity.

Risk Occurrence Probability	P-value	Risk Loss Severity	L-value
Completely foreseeable	9	Severe loss	9
Highly likely	7	Major loss	7
Likely and frequent	5	Moderate loss	5
Possible but infrequent	3	Minor loss	3
Rarely possible	1	Very slight loss	1
Completely impossible	0	No loss	0
Intermediate values between two adjacent judgments	2, 4, 6, 8	Intermediate values between two adjacent judgments	2, 4, 6, 8

**Fig 4:**
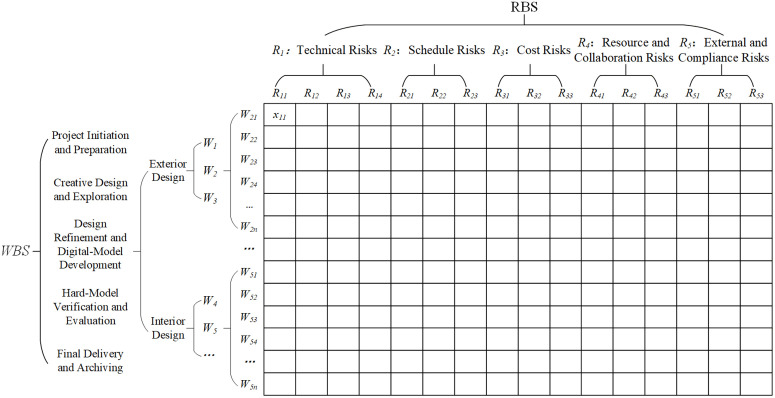
WBS-RBS Risk Matrix for Automotive Styling Design.

In combination with the WBS decomposition and risk factor analysis described in the preceding sections, a WBS-RBS matrix for the automotive styling design process is constructed, as shown in Fig 4. The cell values $x_{ij}$ in the matrix are determined based on expert scoring by an automotive styling design specialist panel from a major automobile manufacturer. To ensure the scientific validity and reliability of the scoring results for the risk occurrence probability $P_{ij}\;$and loss severity $L_{ij}\;$in the WBS-RBS matrix, and to avoid potential risk overlap and repeated measurement among different risk factors, this study adopts a collective expert evaluation approach to assign risk parameters for each design task node. In addition, a risk identification and overlap verification mechanism is introduced during the scoring process. The expert panel consists of seven members, all from well-known domestic automobile manufacturers’ styling design and related support departments. Experts were selected based on their professional experience, job relevance, and participation in vehicle development projects. The panel covers the fields of styling design, styling engineering interface, and project management. Specifically, three members are exterior/interior styling design experts with 8–15 years of experience in vehicle development, two are styling engineering interface specialists familiar with engineering feasibility and manufacturing constraints, and the remaining two are product project managers or styling management personnel.

During the evaluation process, experts independently scored the risk occurrence probability and loss severity for each WBS task node under different RBS risk factors according to unified risk definitions and scoring criteria (see Table 1), combined with their experience in automotive styling design projects. To reduce the interference of risk overlap on the evaluation results, the scoring results were cross-checked through group discussions to identify potential duplicate evaluations or logical conflicts among different risk factors. The relevant scores were then adjusted according to the underlying mechanisms of risk occurrence to mitigate the influence of overlapping risks. In addition, to reduce subjective bias, multiple rounds of discussion and result aggregation were conducted. The final scores were obtained by averaging the opinions of all experts and used as the input parameters of the matrix, thereby improving the reliability and robustness of the risk evaluation results.

## 4 Risk identification for automotive styling-design tasks

### 4.1 Determining risk-factor weights with improved FAHP

Automotive styling design projects involve numerous and complex risk factors. When the AHP is directly applied to identify risk nodes, two main problems arise. First, it is difficult to capture the inherent fuzziness of human cognition when assessing relationships among factors. Second, when the judgment matrix fails to satisfy the consistency requirement, repeated procedures of adjustment, verification, readjustment, and re-verification are required. Although FAHP introduces fuzzy numbers to better describe the fuzziness of expert judgments, it still suffers from strict consistency requirements, complex computation, and insufficient stability of results. Therefore, this study adopts an improved FAHP method to determine the risk weights [40]. This method is based on constructing a fuzzy consistency matrix and solving the weight vector using vector norms. The fuzzy consistency matrix directly calculates weights through vector norms, thereby avoiding the consistency testing and eigenvector iteration procedures required in traditional AHP methods and significantly reducing computational complexity. In addition, the infinity norm avoids complex eigenvalue iteration operations and improves computational efficiency and numerical stability while maintaining ranking consistency. As a result, the accumulation of computational errors is reduced, and the reliability of weight results in automotive styling design risk evaluation is enhanced. Through fuzzy representation, matrix consistency transformation, and norm-based weight calculation, the improved FAHP method overcomes the shortcomings of traditional FAHP methods, such as complicated consistency testing, large computational burden, and high sensitivity of results. Consequently, it is more suitable for multi-criteria evaluation problems characterized by strong subjectivity and uncertainty, such as the risk assessment of automotive styling design tasks. The construction procedure is described as follows.

**Step 1:** Construct Fuzzy Judgment Matrix

Based on the WBS-RBS matrix described in Sect 3.3, according to the Eqs (2) and (3) construct fuzzy judgment matrix A for all factors.


$$\begin{array}{c} a_{ij}=\left\{ \begin{array}{cc} \text{0}.\text{5} & s(i)=s(j)\\ \text{1}.\text{0} & s(i)>s(j)\\ \text{0} & s(i)<s(j) \end{array}\right. \end{array}$$ (2)

$$\begin{array}{c} \begin{array}{c} A=(a_{ij}{)}_{n\times n}=[\begin{array}{ccc} a_{\text{1}\text{1}} & \cdots & a_{\text{1}n}\\ \vdots & \ddots & \vdots \\ a_{m\text{1}} & \cdots & a_{mn} \end{array}] \end{array} \end{array}$$ (3)

Here, s(i) and s(j) denote the relative importance of risk factor *i* and *j*, respectively, and the relative importance is expressed by the degree of risk at a given task node associated with the corresponding risk factor. The comparison result of risk factor iii relative to factor j is denoted by $a_{ij}$.

**Step 2:** Calculate the Consistency Judgment Matrix

First, sum the elements of fuzzy judgment matrix A row by row to obtain $p_{i}=\sum_{j=\text{1}}^{n}a_{ij}$, Then The following mathematical transformation was applied: $p_{ij}=\left[\left(p_{i}-p_{j}\right)/\text{2}n\right]+\text{0}.\text{5}$, resulting in the consistency judgment matrix $P={\left[p_{ij}\right]}_{n\times n}$. The construction of a consistent matrix enables the elements of the matrix to satisfy additive or multiplicative consistency relationships. This property avoids the computational burden and instability caused by the strict consistency tests required in traditional AHP. Moreover, the proposed approach does not rely on the eigenvector iterative solution commonly used in classical FAHP methods. Instead, the weight vector is directly calculated through the norm of the matrix row vectors, thereby significantly reducing computational complexity.

**Step 3:** Initialize the weight Vector

Apply the row-sum normalization method to derive the initial weight vector${\;w}^{\left(\text{0}\right)}={\left(w_{\text{1}}{,w}_{\text{2}},\ldots ,w_{n}\right)}^{T}$,using the following Eq (4).


$$\begin{array}{c} w^{i}=\sum_{j=\text{1}}^{n}\frac{p_{ij}}{\sum_{i=\text{1}}^{n}\sum_{j=\text{1}}^{n}p_{ij}} \end{array}$$ (4)

**Step 4:** Construct the Reciprocal Matrix

Transform the fuzzy consistency judgment matrix $P={\left[p_{ij}\right]}_{n\times n}$ into reciprocal matrix $E={\left[e_{ij}\right]}_{n\times n}$ by $e_{ij}=p_{ij}/p_{ji}$.

**Step 5:** Iterative Solution of Final Weight Vector

Take the initial weight vector $w^{\left(\text{0}\right)}\;$as the initial vector *V* ^*0*^ for the power iteration method, and further compute a more accurate weight vector $V^{k}$. Given the initial vector ${\;V}^{\text{0}}={\left(V_{\text{0}\text{1}}{,V}_{\text{0}\text{2}},\ldots ,V_{\text{0}n}\right)}^{T}$, use the iterative formula *V* ^*k+1*^ *= EV* ^*k*^ to obtain *V* ^*k+1*^. Calculate the infinity norm ${\left\|V_{k+\text{1}}\right\|}_{\infty }$. If $\left|{\left\|V_{k+\text{1}}\right\|}_{\infty }-{\left\|V_{k}\right\|}_{\infty }\right|<\epsilon$, then ${\left\|V_{k+\text{1}}\right\|}_{\infty }$ is regarded as the maximum eigenvalue $\lambda_{max}$. After normalizing $V^{k+\text{1}}$ according to the Eq (5), the resulting vector $w^{\left(k\right)}=V^{\left(k+\text{1}\right)}$ is obtained as the weight vector, and the iteration process terminates. For a non-negative consistent matrix, the sum of the elements in each row vector (the infinity norm) reflects the relative importance of the corresponding criterion compared with other criteria, while maintaining directional consistency with the principal eigenvector of the matrix. According to the Perron-Frobenius theorem, the largest eigenvalue of a positive matrix corresponds to a unique positive eigenvector. Therefore, normalization based on the infinity norm can approximate this principal eigenvector without explicitly solving for the eigenvalues, thereby enabling efficient weight calculation.


$$\begin{array}{c} V^{k+\text{1}}={\left[\frac{V_{k+\text{1},\text{1}}}{\sum_{i=\text{1}}^{n}V_{k+\text{1},i}},\frac{V_{k+\text{1},\text{2}}}{\sum_{i=\text{1}}^{n}V_{k+\text{1},i}},\ldots ,\frac{V_{k+\text{1},n}}{\sum_{i=\text{1}}^{n}V_{k+\text{1},i}}\right]}^{\text{T}} \end{array}$$ (5)

If the convergence condition is not satisfied, calculate $V^{k}$ using the following formula (6) as the new initial vector for the next iteration.


$$\begin{array}{c} V^{k}=V_{k+\text{1}}/\|V_{k+\text{1}}{\|}_{\infty }={\left[V_{k+\text{1},\text{1}}/\|V_{k+\text{1}}{\|}_{\infty },V_{k+\text{1},\text{2}}/\|V_{k+\text{1}}{\|}_{\infty },\ldots ,V_{k+\text{1},n}/\|V_{k+\text{1}}{\|}_{\infty }\right]}^{T} \end{array}$$ (6)

### 4.2 Quantifying design-task risks using the VIKOR method

The risks associated with automotive styling design tasks involve multiple mutually conflicting criteria, including creative alignment, cost control, and schedule progress. Among MCDM methods, commonly used ranking approaches include Technique for Order Preference by Similarity to Ideal Solution (TOPSIS), Preference Ranking Organization Method for Enrichment Evaluation (PROMETHEE), and VIKOR, which differ in theoretical assumptions and decision preferences. The VIKOR method is based on the concept of a compromise solution. It seeks a balance between maximizing group utility and minimizing individual regret and is capable of highlighting the influence of the most unfavorable risk factors. Therefore, it is suitable for decision-making scenarios involving critical bottleneck risks [41]. By contrast, TOPSIS assumes full compensability among indicators. However, in automotive styling design, severe problems related to regulatory compliance or engineering feasibility often cannot be offset by advantages in other indicators [42]. PROMETHEE relies on the specification of preference functions and threshold parameters, and its results are sensitive to parameter selection, which may reduce the stability and consistency of the ranking results [43]. In contrast, the VIKOR method introduces the individual regret indicator, preventing advantages in certain indicators from completely offsetting severe deficiencies in others. Furthermore, it allows the decision coefficient v to be adjusted to reflect different risk preference scenarios, providing better interpretability and operability. In addition, risks in automotive styling design tasks exhibit strong stage characteristics. Different stages emphasize different aspects of overall risk control and critical risk avoidance. The VIKOR method can reflect such changes in decision preference simply by adjusting the parameter vwithout modifying the original risk matrix structure. This feature makes it suitable for capturing the dynamic nature of styling design task risks and avoiding risk misjudgment caused by single-dimensional evaluation.

The specific procedure for calculating the WBS node risk level using the VIKOR method is as follows:

**Step 1:** Standardize the WBS-RBS matrix

Each cell value in the WBS-RBS matrix is normalized using range normalization for each indicator, as expressed below.


$$\begin{array}{c} z_{ij}=\frac{x_{ij}-{min}_{j}}{{max}_{j}-{min}_{j}} \end{array}$$ (7)

where Z_ij_ denotes the normalized value of $x_{ij}$ represents the value of the j-th risk factor for the i-th WBS node; and$\;{max}_{j}$, ${min}_{j}$ represent the maximum and minimum values of column *j*, respectively.

**Step 2:** Calculate the positive and negative ideal solutions

For each column j of the normalized WBS-RBS matrix, determine the positive and negative ideal solutions, i.e., the maximum and minimum values in that column ($max\;z_{j}$, $min\;z_{j}$).

**Step 3:** Calculate group utility S and individual regret R

*S* represents the sum of the relative distances from the optimal object and reflects the overall deviation of an evaluated object from the positive ideal solution, with smaller values indicating better performance. *R* represents the maximum relative distance of any single indicator from the optimal object, where smaller values again correspond to superior performance.


$$\begin{array}{c} S_{i}=\sum_{j=\text{1}}^{n}\left(w_{j}\frac{max\;z_{j}-z_{ij}}{max\;z_{j}-min\;z_{j}}\right)\;R_{i}=\underset{\text{1}\leq j\leq n}{\text{max}}\;w_{j}\frac{max\;z_{j}-z_{ij}}{max\;z_{j}-min\;z_{j}} \end{array}$$ (8)

Where $w_{j}$ denotes the weight of the *j*-th risk factor.

**Step 4:** Calculate the benefit ratio *Q*

Based on the obtained $S_{i}$ and $R_{i}$, compute the benefit ratio $Q_{i}\;$by using the following Eq (9).


$$\begin{array}{c} Q_{i}=v\left[\frac{\left(S_{i}-min\;S_{i}\right)}{\left(max\;S_{i}-min\;S_{i}\right)}\right]+(\text{1}-v)\left[\frac{\left(R_{i}-min\;R_{i}\right)}{\left(max\;R_{i}-min\;R_{i}\right)}\right] \end{array}$$ (9)

where $Q_{i}$ denotes the benefit ratio; *v* denotes the decision-making coefficient with a value range of [0,1]. When v>0.5, the evaluation result is more inclined to group utility and therefore tends toward risk preference; when v=0.5, equal consideration is given to group utility and individual regret, representing a risk-balanced pattern; when v<0.5, emphasis is placed on individual regret, thereby reflecting a risk-averse pattern. $maxS_{i}$ and $minS_{i}$ denote the maximum and minimum values of S, while $maxR_{i}$ and $minR_{i}$ denote the maximum and minimum values of *R*.

**Step 5:** Determine the ranking of WBS nodes

Sort the calculated *Q* values in ascending order, where a higher *Q* indicates higher compatibility with design objectives and therefore lower risk, and vice versa. The classification of risk levels is established based on the characteristics of the benefit ratio Q derived from the VIKOR method. It also draws on the risk grading principles of ISO 31000 and ISO 31010, as well as the interval division concept of the LEC evaluation method, while incorporating the specific risk distribution characteristics of automotive styling design tasks. By categorizing the continuous evaluation results into three levels—high, medium, and low risk—the proposed scheme not only enhances the interpretability of the risk identification results but also facilitates hierarchical decision-making and resource allocation in engineering management. The detailed criteria are shown in Table 2.

**Table 2:** Risk level classification criteria for automotive styling design.

Benefit ratio *Q*	Risk identification result	Recommended handling
0-0.3	High risk	Significant risk likely to cause major rework, substantial cost increase, or compliance issues; immediate design adjustment required
0.3-0.7	Medium risk	Moderate risk likely to cause partial rework; additional resources or targeted mitigation plan required
0.7-1	Low risk	Minor risk; minimal conflict among styling tasks; no additional mitigation measures required

## 5 Case study: Exterior styling concept-design tasks of an SUV

**Fig 5:**
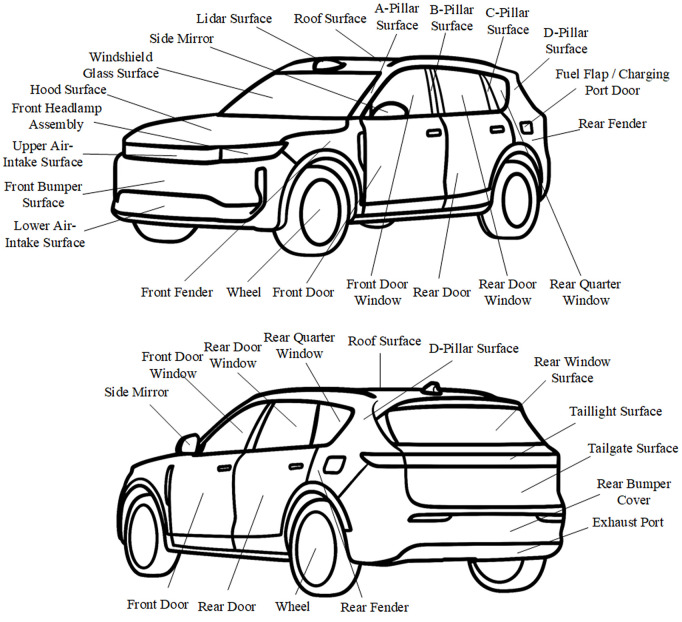
Creative Exterior Styling Design Task Classification for a Certain SUV.

To validate the proposed FAHP-VIKOR risk identification method, a case study is conducted using the exterior styling concept-design phase of a five-seat, three-box SUV. The exterior styling tasks include refinement of the front-end styling, delineation of the vehicle body contours, and sculpting of the rear-end form. Key components include the grille, headlamps, side profiles, taillamps, and other styling elements, forming a complete task system (see Fig 5).

### 5.1 Construction of the WBS-RBS matrix for exterior styling

(1) Establishing the WBS of automotive exterior styling design

Based on the WBS construction process described in Sect 3.1, the creative exterior styling design of an SUV is decomposed into three major categories, namely front-end design, rear-end design, and body design. Each category consists of multiple exterior components, resulting in a total of 27 WBS work packages, as shown in Table 3.

**Table 3:** WBS decomposition of creative exterior styling design for a certain SUV.

Exterior Component Design for Automotive Styling Design Project	Primary Structure	WBS	Secondary Structure	WBS
Front-End Design	*W*_*1*_	Front Headlamp Assembly	*W*_*11*_
		Upper Air-Intake Surface	*W*_*12*_
		Front Bumper Surface	*W*_*13*_
		Lower Air-Intake Surface	*W*_*14*_
		Hood Surface	*W*_*15*_
		Windshield Glass Surface	*W*_*16*_
Body Design	*W*_*2*_	A-Pillar Surface	*W*_*21*_
		B-Pillar Surface	*W*_*22*_
		C-Pillar Surface	*W*_*23*_
		D-Pillar Surface	*W*_*24*_
		Roof Surface	*W*_*25*_
		Lidar Surface	*W*_*26*_
		Front Door Window	*W*_*27*_
		Rear Door Window	*W*_*28*_
		Rear Quarter Window	*W*_*29*_
		Front Door	*W*_*210*_
		Rear Door	*W*_*211*_
		Front Fender	*W*_*212*_
		Rear Fender	*W*_*213*_
		Fuel Flap or Charging Port Door	*W*_*214*_
		Wheel	*W*_*215*_
		Side Mirror	*W*_*216*_
Rear-End Design	*W*_*3*_	Rear Window Surface	*W*_*31*_
		Taillight Surface	*W*_*32*_
		Tailgate Surface	*W*_*33*_
		Rear Bumper Cover	*W*_*34*_
		Exhaust Port	*W*_*35*_

(2) Building the WBS-RBS Matrix

The above 27 lowest-level WBS work packages are taken as the rows of the matrix, and the 16 lowest-level RBS risk factors described in Sect 2.2 are taken as the columns to form the WBS-RBS coupling matrix. The expert panel on automotive styling design then assigns P and L scores for each of the 16 risk factors at each of the 27 WBS task nodes, followed by calculating the risk value $x_{ij}$ for each matrix cell according to Eq (1). The final results are shown in Table 4.

**Table 4:** WBS-RBS risk matrix for creative exterior styling design of an SUV.

WBS Decomposition		RBS Decomposition															
		Technological *R*1				Schedule Risk *R*2			Cost Risk *R*3			Resource and Collaboration Risk *R*4			External and Compliance Risk *R*5		
		*R*11	*R*12	*R*13	*R*14	*R*21	*R*22	*R*23	*R*31	*R*32	*R*33	*R*41	*R*42	*R*43	*R*51	*R*52	*R*53
Front-End Design (*W*_*1*_)	*W*_*11*_	42	35	28	0	30	25	12	0	0	15	8	20	0	16	6	9
	*W*_*12*_	56	42	35	0	35	30	15	0	0	20	12	25	0	20	8	12
	*W*_*13*_	63	49	42	0	42	35	18	0	0	25	15	30	0	24	9	15
	*W*_*14*_	49	35	30	0	30	25	12	0	0	18	9	24	0	18	6	9
	*W*_*15*_	35	28	20	0	25	20	9	0	0	12	6	16	0	12	4	6
	*W*_*16*_	20	15	12	0	15	12	6	0	0	6	3	9	0	8	2	3
Rear-End Design (*W*_*2*_)	*W*_*21*_	30	25	20	0	25	20	9	0	0	15	6	18	0	15	6	9
	*W*_*22*_	20	16	12	0	16	12	6	0	0	9	4	12	0	9	3	4
	*W*_*23*_	25	20	15	0	20	16	8	0	0	12	6	15	0	12	4	6
	*W*_*24*_	18	12	9	0	12	9	4	0	0	6	3	9	0	6	2	3
	*W*_*25*_	15	9	6	0	9	6	3	0	0	4	2	6	0	4	1	2
	*W*_*26*_	49	42	35	0	42	35	18	0	0	30	15	28	0	25	12	18
	*W*_*27*_	12	9	6	0	9	6	3	0	0	4	2	6	0	4	1	2
	*W*_*28*_	15	12	9	0	12	9	4	0	0	6	3	8	0	6	2	3
	*W*_*29*_	12	8	6	0	8	6	3	0	0	4	2	6	0	4	1	2
	*W*_*210*_	25	20	15	0	20	16	8	0	0	12	6	15	0	12	4	6
	*W*_*211*_	24	18	12	0	18	12	6	0	0	9	4	12	0	9	3	4
	*W*_*212*_	28	24	16	0	24	18	9	0	0	15	8	18	0	15	6	9
	*W*_*213*_	20	16	12	0	16	12	6	0	0	9	4	12	0	9	3	4
	*W*_*214*_	9	6	4	0	6	4	2	0	0	3	1	4	0	3	1	1
	*W*_*215*_	30	25	20	0	25	20	9	0	0	18	9	20	0	18	8	12
	*W*_*216*_	18	12	9	0	12	9	4	0	0	8	4	9	0	8	3	4
Body Design (*W*_*3*_)	*W*_*31*_	12	9	6	0	9	6	3	0	0	4	2	6	0	4	1	2
	*W*_*32*_	35	28	20	0	28	24	12	0	0	20	12	24	0	20	9	15
	*W*_*33*_	25	20	15	0	20	16	8	0	0	12	6	15	0	12	4	6
	*W*_*34*_	56	49	42	0	49	42	20	0	0	35	20	35	0	30	15	24
	*W*_*35*_	15	12	9	0	12	9	4	0	0	6	3	8	0	6	2	3

### 5.2 Calculation of risk-factor weights

To illustrate the calculation process, the front headlamp assembly (*W*_*11*_) is used as an example. Because certain risk factors (e.g., *R*_*14*_, *R*_*31*_, *R*_*32*_, *R*_*43*_) do not apply during early styling concept development, they are assigned zero risk. Corresponding judgment matrices *W-R*, *W-R*_*1*_, *W-R*_*2*_*, W-R*_*4*_, and *W-R*_*5*_ are constructed (Table 5).

**Table 5:** Fuzzy judgement Matrix.

judgment matrix of *W-R*	judgment matrix of *W-R*_*1*_	judgment matrix of *W-R*_*2*_	judgment matrix of *W-R*_*4*_	judgment matrix of *W-R*_*5*_
$\left[\begin{array}{ccccc} \text{0}.\text{5} & \text{1}.\text{0} & \text{1}.\text{0} & \text{1}.\text{0} & \text{1}.\text{0}\\ \text{0}.\text{0} & \text{0}.\text{5} & \text{1}.\text{0} & \text{1}.\text{0} & \text{1}.\text{0}\\ \text{0}.\text{0} & \text{0}.\text{0} & \text{0}.\text{5} & \text{0}.\text{0} & \text{0}.\text{0}\\ \text{0}.\text{0} & \text{0}.\text{0} & \text{1}.\text{0} & \text{0}.\text{5} & \text{0}.\text{0}\\ \text{0}.\text{0} & \text{0}.\text{0} & \text{1}.\text{0} & \text{1}.\text{0} & \text{0}.\text{5} \end{array}\right]$	$\left[\begin{array}{ccc} \text{0}.\text{5} & \text{1}.\text{0} & \text{1}.\text{0}\\ \text{0}.\text{0} & \text{0}.\text{5} & \text{1}.\text{0}\\ \text{0}.\text{0} & \text{0}.\text{0} & \text{0}.\text{5} \end{array}\right]$	$\left[\begin{array}{ccc} \text{0}.\text{5} & \text{1}.\text{0} & \text{1}.\text{0}\\ \text{0}.\text{0} & \text{0}.\text{5} & \text{1}.\text{0}\\ \text{0}.\text{0} & \text{0}.\text{0} & \text{0}.\text{5} \end{array}\right]$	$\left[\begin{array}{cc} \text{0}.\text{5} & \text{0}.\text{0}\\ \text{1}.\text{0} & \text{0}.\text{5} \end{array}\right]$	$\left[\begin{array}{ccc} \text{0}.\text{5} & \text{1}.\text{0} & \text{1}.\text{0}\\ \text{0}.\text{0} & \text{0}.\text{5} & \text{0}.\text{0}\\ \text{0}.\text{0} & \text{1}.\text{0} & \text{0}.\text{5} \end{array}\right]$

Using the *W-R* judgment matrix as an example, the corresponding consistency judgment matrix *P* is obtained as follows.


$$\begin{array}{c} P=[\begin{array}{ccccc} \text{0}.\text{5} & \text{0}.\text{6} & \text{0}.\text{9} & \text{0}.\text{8} & \text{0}.\text{7}\\ \text{0}.\text{4} & \text{0}.\text{5} & \text{0}.\text{8} & \text{0}.\text{7} & \text{0}.\text{6}\\ \text{0}.\text{1} & \text{0}.\text{2} & \text{0}.\text{5} & \text{0}.\text{4} & \text{0}.\text{3}\\ \text{0}.\text{2} & \text{0}.\text{3} & \text{0}.\text{6} & \text{0}.\text{5} & \text{0}.\text{4}\\ \text{0}.\text{3} & \text{0}.\text{4} & \text{0}.\text{7} & \text{0}.\text{6} & \text{0}.\text{5} \end{array}] \end{array}$$

The initial weight vector $w^{\left(\text{0}\right)}$ obtained by the row sum normalization method is as follows.


$$\begin{array}{c} w^{\left(\text{0}\right)}=[\text{0}.\text{2}\text{8},\text{0}.\text{2}\text{4},\text{0}.\text{1}\text{2},\text{0}.\text{1}\text{6},\text{0}.\text{2}\text{0}{]}^{T} \end{array}$$

The reciprocal judgment matrix *E* is as follows.


$$\begin{array}{c} E=[\begin{array}{ccccc} \text{1}.\text{0}\text{0}\text{0}\text{0} & \text{1}.\text{5}\text{0}\text{0}\text{0} & \text{9}.\text{0}\text{0}\text{0}\text{0} & \text{4}.\text{0}\text{0}\text{0}\text{0} & \text{2}.\text{3}\text{3}\text{3}\text{3}\\ \text{0}.\text{6}\text{6}\text{6}\text{7} & \text{1}.\text{0}\text{0}\text{0}\text{0} & \text{4}.\text{0}\text{0}\text{0}\text{0} & \text{2}.\text{3}\text{3}\text{3}\text{3} & \text{1}.\text{5}\text{0}\text{0}\text{0}\\ \text{0}.\text{1}\text{1}\text{1}\text{1} & \text{0}.\text{2}\text{5}\text{0}\text{0} & \text{1}.\text{0}\text{0}\text{0}\text{0} & \text{0}.\text{6}\text{6}\text{6}\text{7} & \text{0}.\text{4}\text{2}\text{8}\text{6}\\ \text{0}.\text{2}\text{5}\text{0}\text{0} & \text{0}.\text{4}\text{2}\text{8}\text{6} & \text{1}.\text{5}\text{0}\text{0}\text{0} & \text{1}.\text{0}\text{0}\text{0}\text{0} & \text{0}.\text{6}\text{6}\text{6}\text{7}\\ \text{0}.\text{4}\text{2}\text{8}\text{6} & \text{0}.\text{6}\text{6}\text{6}\text{7} & \text{2}.\text{3}\text{3}\text{3}\text{3} & \text{1}.\text{5}\text{0}\text{0}\text{0} & \text{1}.\text{0}\text{0}\text{0}\text{0} \end{array}] \end{array}$$

With a convergence precision of 0.0001 and four iterations, the final weights obtained by iterative computation are as follows.


$$\begin{array}{c} w=[\text{0}.\text{4}\text{2}\text{5}\text{3},\text{0}.\text{2}\text{5}\text{0}\text{5},\text{0}.\text{0}\text{6}\text{1}\text{3},\text{0}.\text{1}\text{0}\text{2}\text{5},\text{0}.\text{1}\text{6}\text{0}\text{4}{]}^{T} \end{array}$$

Following this procedure, both the risk factors and risk indicators weights are obtained, as shown in Table 6. Ranking the indicators further determines which risk factors dominate at this node.

**Table 6:** Risk factor weights for creative exterior styling design of an SUV.

Risk Factors (Weight)	Risk Indicators	Weight	Risk Value	Aggregate Weight	Rank
Technological Risk *R*_*1*_ (0.4253)	*R*_*11*_	0.5954	42	0.2532	1
	*R*_*12*_	0.2763	35	0.1175	3
	*R*_*13*_	0.1283	28	0.0546	8
Schedule Risk *R*_*2*_ (0.2505)	*R*_*21*_	0.5954	30	0.1491	2
	*R*_*22*_	0.2763	25	0.0692	6
	*R*_*23*_	0.1283	12	0.0321	10
Cost Risk *R*_*3*_ (0.0613)	*R*_*33*_	1.00	15	0.0613	7
Resource & Collaboration Risk R4 (0.1025)	*R*_*41*_	0.25	8	0.0256	11
	*R*_*42*_	0.75	20	0.0769	5
External & Compliance Risk *R*_*5*_ (0.1604)	*R*_*41*_	0.5954	16	0.0955	4
	*R*_*42*_	0.1283	6	0.0206	12
	*R*_*43*_	0.2763	9	0.0443	9

For the creative design of the front headlamp assembly (*W*_*11*_), the results obtained from the improved FAHP-based weight calculation indicate that technical risk accounts for the largest proportion, followed by schedule risk, while cost risk contributes the least. This suggests that the risk at this task node is primarily dominated by the difficulty of styling realization and engineering feasibility constraints, with the design iteration cycle also exerting a significant influence on the overall risk level. Further sensitivity analysis reveals that small variations in the weight of technical risk lead to the most pronounced changes in the overall risk ranking, whereas fluctuations in cost risk weights have a relatively limited impact. This demonstrates that the proposed model exhibits high sensitivity to critical risk factors and is capable of effectively identifying the dominant drivers of task level risk, thereby validating both the rationality of the weight allocation and the robustness of the model results.

### 5.3 Quantitative risk assessment and analysis

According to Eq (7), the WBS-RBS matrix is standardized, from which the maximum and minimum values of each column (${max}_{j}$, ${min}_{j}$) are obtained. Combined with the FAHP derived risk weights at *W*_*35*_, the group utility *S*_*35*_ and individual regret *R*_*35*_ are calculated using Eq (8), the same procedure is applied to the remaining 26 WBS nodes.

In the risk evaluation of automotive styling design tasks, regulatory compliance and engineering feasibility risks often exhibit a “veto effect,” while schedule and cost risks mainly reflect overall project performance. If a large value of v(v>0.7) is adopted, the ranking results will tend to favor solutions with the optimal overall average risk level, which may mask critical risk factors. Conversely, if a small value of v(v<0.3) is used, the evaluation results may be excessively influenced by a single indicator, reducing the comprehensiveness of the assessment [44]. Therefore, this study sets v=0.5 to achieve a balanced trade-off between group utility and individual regret, which better conforms to the decision-making requirements of automotive styling design, where both overall optimization and critical risk control must be considered. Finally, based on Eq (9), the benefit ratio Q of each node is computed and subsequently ranked. According to Table 2, each WBS node is then classified into corresponding risk levels. The results are summarized in Table 7.

**Table 7:** Summary of VIKOR results (risk-balanced type).

WBS Node	S	R	Q	Rank	Risk Level
*W*_*11*_	0.4513	0.0985	0.3781	22	Medium Risk
*W*_*12*_	0.2745	0.0486	0.1778	25	High Risk
*W*_*13*_	0.1263	0.0243	0.0483	26	High Risk
*W*_*14*_	0.3880	0.0663	0.2753	23	High Risk
*W*_*15*_	0.5855	0.1315	0.5195	20	Medium Risk
*W*_*16*_	0.8156	0.2016	0.7912	10	Low Risk
*W*_*21*_	0.5894	0.1547	0.5721	18	Medium Risk
*W*_*22*_	0.7865	0.2020	0.7770	11	Low Risk
*W*_*23*_	0.6926	0.1782	0.6765	13	Medium Risk
*W*_*24*_	0.8612	0.2110	0.8352	8	Low Risk
*W*_*25*_	0.9297	0.2251	0.9013	5	Low Risk
*W*_*26*_	0.2028	0.0657	0.1780	24	High Risk
*W*_*27*_	0.9437	0.2392	0.9392	3	Low Risk
*W*_*28*_	0.8798	0.2255	0.8764	6	Low Risk
*W*_*29*_	0.9500	0.2392	0.9424	2	Low Risk
*W*_*210*_	0.6926	0.1782	0.6765	15	Medium Risk
*W*_*211*_	0.7553	0.1832	0.7199	14	Low Risk
*W*_*212*_	0.6117	0.1641	0.6040	17	Medium Risk
*W*_*213*_	0.7865	0.2020	0.7770	11	Low Risk
*W*_*214*_	1.0000	0.2537	1.0000	1	Low Risk
*W*_*215*_	0.5553	0.1547	0.5545	19	Medium Risk
*W*_*216*_	0.8483	0.2110	0.8286	9	Low Risk
*W*_*31*_	0.9437	0.2392	0.9392	3	Low Risk
*W*_*32*_	0.4685	0.1313	0.4585	21	Medium Risk
*W*_*33*_	0.6926	0.1782	0.6765	15	Medium Risk
*W*_*34*_	0.0328	0.0328	0.0186	27	High Risk
*W*_*35*_	0.8798	0.2255	0.8764	6	Low Risk

From Table 7 there are five high-risk nodes (18.5%), nine medium-risk nodes (33.3%), and thirteen low-risk nodes (48.2%). The high-risk nodes include *W*_*12*_ (upper air-intake surface), *W*_*13*_ (front bumper surface), *W*_*14*_ (lower air-intake surface), *W*_*26*_ (lidar surface), and *W*_*34*_ (rear bumper surface). These nodes show a high level of risk across most of the five major dimensions. Specifically, the front-end design tasks *W*_*12*_, *W*_*13*_, and *W*_*14*_ show relatively high technical risk (*R*_*1*_) and schedule risk (*R*_*2*_). In order to enhance brand recognition, designers often adopt relatively aggressive styling languages, which may lead to deviations from the brand DNA (*R*_*11*_: stylistic deviation from brand DNA) and generate conflicts with engineering feasibility (*R*_*13*_: abrupt engineering hard-points) and aerodynamic performance (*R*_*12*_: insufficient CAS surface continuity). As a result, these tasks frequently undergo repeated modifications during cross-departmental reviews (*R*_*22*_: Prolonged review and decision-making chain), which may ultimately lead to schedule delays and increased project costs. Node *W*_*26*_, which is associated with intelligent and connected vehicle components, presents risks not only from the technical challenges of integrating sensor layouts with styling design (*R*_*1*_), but also from potential regulatory changes (*R*_*51*_: regulatory updates). Adjustments in regulatory requirements may trigger design rework. Similar to the front-end design tasks, node *W*_*34*_ requires repeated trade-offs between compliance with crash regulations (*R*_*51*_) and maintaining desired styling proportions (*R*_*11*_). In addition, it involves strong coordination constraints with components such as taillights and exhaust systems, which increases the likelihood of design rework and introduces additional schedule and cost risks.

The medium-risk nodes include *W*_*11*_, *W*_*15*_, *W*_*21*_, *W*_*23*_, *W*_*210*_, *W*_*212*_, *W*_*215*_, *W*_*32*_ and *W*_*33*_, whose risks characteristics are relatively dispersed and are typically concentrated in specific risk factors. For instance, nodes *W*_*11*_ and *W*_*32*_ are mainly affected by the technical implementation challenges associated with complex light-and-shadow styling surfaces (*R*_*12*_) and the cost pressure of special materials (*R*_*33*_: Material price fluctuation). Structural body components such as *W*_*15*_, W_21_, and *W*_*23*_ are primarily constrained by the interactions among styling requirements, ergonomics, and safety regulations (*R*_*13*_*, R*_*51*_), which may lead to localized rework and schedule impacts. Furthermore, the risks associated with nodes *W*_*210*_*, W*_*212*_, and *W*_*33*_ are mainly reflected in resource coordination and collaboration factors (*R*_*42*_: lack of cross-departmental interface personnel). Issues such as panel gaps and surface mismatches often increase communication and iteration costs during the design process. Node *W*_*215*_, on the other hand, involves trade-offs between lightweight design and structural strength, making its technical risk (*R*_*1*_) and cost risk (*R*_*3*_) relatively prominent.

From the perspective of risk distribution, high-risk nodes are mainly concentrated in front-end design, rear-end design, and intelligent component design such as the lidar surface. Medium-risk nodes are primarily associated with body structural components and optical elements, while low-risk nodes mainly correspond to components with mature technologies and weak dependency relationships, such as the fuel filler or charging port door and vehicle windows. This distribution pattern is consistent with engineering practice in automotive styling design. The front-end and rear-end regions represent the core elements of vehicle styling identity, where design complexity is high and conflicts with engineering feasibility and regulatory requirements are more likely to occur, resulting in higher risk levels. Intelligent components such as the lidar surface are subject to both technological constraints and regulatory requirements, which also leads to elevated risks. Body structural components must balance styling requirements, ergonomics, and safety considerations, and therefore involve a moderate level of risk. In contrast, the design processes of mature components are highly standardized, leading to relatively lower risk levels.

From the perspective of risk driving mechanisms, high and medium risk nodes are mainly concentrated in three categories. First, tasks with high design complexity, represented by front and rear styling, where risks arise from the strong coupling between styling innovation and multiple engineering constraints. Second, tasks driven by engineering and manufacturing constraints, such as body structural components, where risks primarily stem from conflicts between manufacturability and regulatory compliance. Third, tasks characterized by technological uncertainty, represented by lidar related surfaces, where risks are influenced by both the integration of emerging technologies and potential regulatory changes. In addition, some medium risk nodes exhibit pronounced cross departmental coordination characteristics, further contributing to their risk profiles. The detailed classification of WBS nodes is presented in Table 8.

**Table 8:** Classification of medium-risk and high-risk nodes.

Classification Dimension	High-Risk Nodes	Medium-Risk Nodes
Design complexity	*W*_*12*_ *, W*_*13*_ *, W*_*14*_ *, W*_*34*_	*W*_*11*_ *, W*_*32*_
Manufacturing constraints	*W*_*34*_	*W*_*15*_ *, W*_*21*_ *, W*_*23*_ *, W*_*210*_ *, W*_*212*_ *, W*_*33*_
Technological uncertainty	*W*_*26*_	*W*_*215*_
Collaboration and resource driven	*–*	*W*_*210*_ *, W*_*212*_ *, W*_*33*_

### 5.4 Comparative analysis

To further verify the effectiveness and superiority of the proposed FAHP-VIKOR method in identifying risks in automotive styling design tasks, three different evaluation methods are selected for comparative analysis: (1) the single FAHP weighted evaluation method [11]; (2) the FAHP-TOPSIS method [45], This method ranks risks by calculating the relative closeness coefficient (CC) of each alternative to the positive and negative ideal solutions, where a larger CC value indicates a higher level of risk; (3) the traditional FAHP–VIKOR method [44]; (4) the improved FAHP-VIKOR method proposed in this study. All four methods are based on the same WBS-RBS risk matrix and the same FAHP-derived risk factor weights to ensure that differences in the results arise solely from the ranking mechanisms. The code for implementing each method can be found in the supplementary S1 File. The initial WBS nodes classification used in the study and the original WBS-RBS risk matrix are presented in the supplementary S2 File. The matrices, weight values, return rates, and risk levels of each WBS task node involved in the calculation process of each method in the study can be found in the supplementary S3 File. The specific risk identification results are shown in Table 9.

**Table 9:** Risk identification results of automotive styling task nodes under different methods.

WBS node	FAHP			FAHP-TOPSIS			FAHP-VIKOR			Improved FAHP-VIKOR		
	Risk Value	Rank	Risk Level	CC	Rank	Risk Level	Q	Rank	Risk Level	Q	Rank	Risk Level
*W*_*11*_	29.37	22	High	0.5675	22	Medium	0.3064	22	Medium	0.3781	22	Medium
*W*_*12*_	38.49	25	High	0.7446	24	High	0.1407	24	High	0.1778	25	High
*W*_*13*_	43.37	27	High	0.8613	26	High	0.0327	26	High	0.0483	26	High
*W*_*14*_	33.59	23	High	0.6334	23	Medium	0.2695	23	High	0.2753	23	High
*W*_*15*_	24.20	20	Medium	0.4383	20	Medium	0.4379	19	Medium	0.5195	20	Medium
*W*_*16*_	14.09	10	Medium	0.1951	10	Low	0.7297	8	Low	0.7912	10	Low
*W*_*21*_	22.18	18	Medium	0.4100	18	Medium	0.4584	18	Medium	0.5721	18	Medium
*W*_*22*_	14.56	12	Medium	0.2145	11	Low	0.6447	12	Medium	0.7770	11	Low
*W*_*23*_	18.18	16	Medium	0.3077	14	Medium	0.5579	14	Medium	0.6765	14	Medium
*W*_*24*_	12.10	9	Medium	0.1519	8	Low	0.7984	6	Low	0.8352	8	Low
*W*_*25*_	9.85	5	Medium	0.0884	5	Low	0.9599	2	Low	0.9013	5	Low
*W*_*26*_	37.08	24	High	0.7816	25	High	0.1391	25	High	0.1780	24	High
*W*_*27*_	8.40	4	Low	0.0619	3	Low	0.8854	4	Low	0.9392	3	Low
*W*_*28*_	10.86	7	Medium	0.1209	6	Low	0.7294	9	Low	0.8764	7	Low
*W*_*29*_	8.06	2	Low	0.0559	2	Low	0.8996	3	Low	0.9424	2	Low
*W*_*210*_	18.18	16	Medium	0.3077	14	Medium	0.5579	14	Medium	0.6765	14	Medium
*W*_*211*_	16.84	13	Medium	0.2604	13	Low	0.6789	11	Medium	0.7199	13	Low
*W*_*212*_	21.07	17	Medium	0.3838	17	Medium	0.4906	17	Medium	0.6040	17	Medium
*W*_*213*_	14.56	12	Medium	0.2145	11	Low	0.6447	12	Medium	0.7770	11	Low
*W*_*214*_	6.01	1	Low	0.0034	1	Low	0.9753	1	Low	1.0000	1	Low
*W*_*215*_	22.19	19	Medium	0.4324	19	Medium	0.4359	20	Medium	0.5545	19	Medium
*W*_*216*_	12.09	8	Medium	0.1573	9	Low	0.7799	7	Low	0.8286	9	Low
*W*_*31*_	8.40	4	Low	0.0619	3	Low	0.8854	4	Low	0.9392	3	Low
*W*_*32*_	25.12	21	High	0.5173	21	Medium	0.3422	21	Medium	0.4585	21	Medium
*W*_*33*_	18.18	16	Medium	0.3077	14	Medium	0.5579	14	Medium	0.6765	14	Medium
*W*_*34*_	43.07	26	High	0.9164	27	High	0.0072	27	High	0.0186	27	High
*W*_*35*_	10.86	7	Medium	0.1209	6	Low	0.7294	9	Low	0.8764	6	Low

From the overall results, the three methods show a high degree of consistency in identifying extreme risk nodes. For example, node *W*_*13*_ (front bumper surface) and node *W*_*34*_ (rear bumper surface) are identified as high-risk nodes by all three methods, while node *W*_*214*_ (Fuel Flap or Charging Port Door) consistently falls within the low-risk nodes. This indicates that the constructed WBS-RBS risk matrix is structurally reasonable and that the improved FAHP weighting system exhibits good stability and consistency.

From the results of the single FAHP method, the risk level of each WBS node is calculated through linear weighted summation, which represents a typical fully compensatory model. For example, node *W*_*32*_ is classified as high-risk under FAHP, whereas it is identified as medium-risk under FAHP-TOPSIS, FAHP-VIKOR and the improved FAHP-VIKOR. Similarly, node *W*_*16*_ is classified as medium-risk under FAHP but adjusted to low-risk under the other two methods. These differences indicate that the single weighted model is sensitive to locally high-risk values and may elevate the overall risk level when a particular indicator has a relatively large value.

From the perspective of the FAHP-TOPSIS method, the ranking discrimination is improved compared with the single FAHP method. For example, nodes *W*_*16*_, *W*_*22*_, *W*_*24*_, and *W*_*25*_ are all classified as low-risk under TOPSIS, which better corresponds to the engineering reality that these mature structural modules usually involve relatively low risk. However, compared with the improved FAHP-VIKOR method proposed in this study, differences appear in the high-risk boundary interval (rank positions 22–26). For instance, node *W*_*14*_ is identified as medium-risk under TOPSIS. The underlying reason is that TOPSIS evaluates alternatives based on the Euclidean distance from positive and negative ideal solutions. As long as the overall distance is relatively favorable, an alternative may obtain a better ranking even if it performs poorly on a critical risk factor. While this distance-based compensation mechanism is mathematically reasonable, it may weaken the influence of “critical bottleneck” risks in the context of automotive styling design.

From the results of the FAHP combined with the VIKOR method, it can be observed that although the compromise solution concept of VIKOR is introduced compared with FAHP–TOPSIS, the weight determination process still relies on the traditional FAHP approach. Conventional FAHP requires consistency testing and eigenvector iteration, which become computationally complex when the dimension of the judgment matrix increases. This may lead to convergence issues or distortion in weight allocation. In terms of ranking results, the traditional FAHP–VIKOR and the improved FAHP–VIKOR methods show a generally consistent trend, but differences can be observed for certain nodes. For example, node W22 is classified as medium risk under the traditional FAHP–VIKOR method, whereas it is identified as low risk using the improved FAHP–VIKOR approach. Considering that this node involves fewer styling constraints and a high level of engineering maturity, it is more reasonably classified as low risk. The discrepancy arises because traditional FAHP relies on consistency testing and eigenvector solutions, without incorporating consistency transformation and norm-based optimization, resulting in limited weight stability and the amplification of local errors during the VIKOR ranking process.

Overall, based on the data presented in the table, it can be concluded that the single FAHP method is sensitive to local extreme values, while the FAHP–TOPSIS method suffers from the limitation of full compensability. Although the FAHP–VIKOR method alleviates this issue to some extent, its results remain affected by the instability of weight calculation. In contrast, the improved FAHP–VIKOR method proposed in this study enhances both the weight determination process and the ranking mechanism, producing results that better reflect the engineering reality that critical risks are non-compensatory in automotive styling design tasks. Therefore, in terms of rationality, stability, and interpretability of risk identification, the improved FAHP–VIKOR method demonstrates stronger applicability and decision-support capability in the context of this study.

### 5.5 Management recommendations

**Fig 6:**
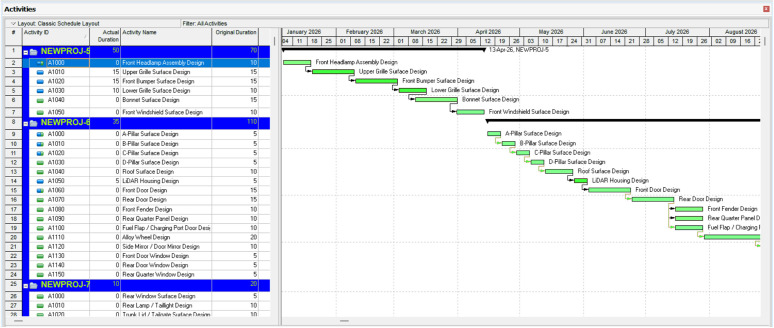
Gantt chart of WBS tasks for the automotive styling design project before risk identification.

In the management of automotive styling design projects, traditional project scheduling is mainly based on experience to determine task durations, which makes it difficult to fully reflect the influence of potential risks on task execution. Therefore, integrating risk identification results into project schedule management is of great significance for improving the efficiency of design management. To visually demonstrate the supporting role of risk identification results in project management decision-making, this study first constructs a WBS based task Gantt chart of the automotive styling design project prior to risk identification in the Oracle Primavera P6 Enterprise Project Portfolio Management (Oracle P6 EPPM) system, based on the Work Breakdown Structure (WBS) of the automotive styling design project (as shown in Fig 6).

**Fig 7:**
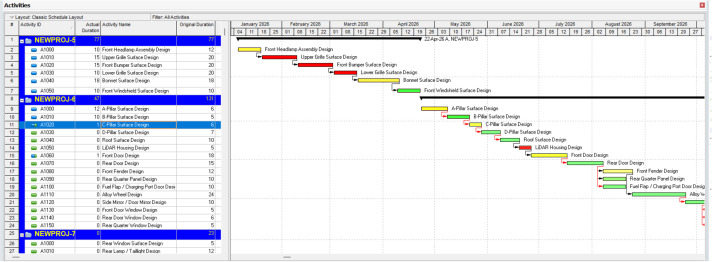
Optimized Gantt chart of WBS tasks for the automotive styling design project after risk identification.

On this basis, the improved FAHP-VIKOR method is applied to conduct risk identification for the WBS tasks of the styling design project. In the Oracle P6 EPPM environment, tasks with different risk levels are marked using different colors. Compared with Fig 6, the optimized Gantt chart (Fig 7) can clearly distinguish the risk levels associated with various styling design tasks.

For high-risk nodes *W*_*12*_*, W*_*13,*_ *W*_*14*_*, W*_*26*_*,* and *W*_*34*_, proactive and strengthened management measures should be adopted. These tasks are typically located at key decision-making stages of the styling design process and are characterized by broad influence and high rework costs. Therefore, greater schedule buffers and design resources should be allocated to these tasks at the early stage of the project. For example, during the front-end styling design stage, the scheme review period can be appropriately extended, and joint verification of engineering feasibility and aerodynamics can be conducted in advance to reduce potential rework caused by engineering constraints or performance issues in later stages. For nodes significantly influenced by regulatory requirements, such as the *W*_*26*_ lidar surface design task, a regulatory monitoring mechanism should be established and sufficient design adjustment space should be reserved to cope with uncertainties arising from potential policy changes.

The management focus for medium-risk nodes lies in targeted control and local optimization. Although these tasks do not directly determine the success or failure of the project, local problems may affect the overall schedule through cumulative effects. For instance, for optical component design nodes such as *W*_*11*_ and *W*_*32*_, coordination between optical performance and styling design should be strengthened. For components involving structural and ergonomic constraints, including nodes *W*_*15*_*, W*_*21*_*,* and *W*_*23*_, early-stage virtual validation can be implemented to reduce potential design conflicts. For nodes with strong cross departmental dependencies, including nodes *W*_*210*_*, W*_*212*_*,* and *W*_*33*_, interface responsibilities should be clearly defined and communication processes optimized to reduce coordination costs.

For low-risk nodes, standardized process management can be adopted. These tasks generally involve mature technologies and relatively weak dependency relationships. Their implementation can be promoted through modular design and experience reuse, thereby allowing more project resources to be concentrated on critical risk nodes and improving overall resource allocation efficiency.

Compared with the original schedule, the optimized Gantt chart allocates time more reasonably for key high-risk tasks and introduces moderate risk-buffer mechanisms, making the overall project schedule more consistent with the risk distribution characteristics of the design tasks.

## 6 Conclusion

After identifying five major risk factors for automotive styling design tasks based on enterprise level design processes and relevant standards, this study moves beyond the traditional focus of risk identification on manufacturing and supply chain stages. An improved FAHP–VIKOR based method is proposed to identify risks in automotive styling design tasks. By shifting risk analysis to the early stage of vehicle styling design, which is a critical decision phase, a systematic risk identification framework at the task level is established. This approach not only enriches the theory of risk management in automotive product development, but also provides practical support for early stage risk control in engineering applications. The proposed method is validated through a case study of an SUV exterior styling design project. The results show that: 1) The WBS decomposition based on design processes and physical structure, combined with the RBS classification derived from international standards and enterprise practices, can comprehensively capture the main sources of risk throughout the styling design process. The WBS–RBS matrix clearly represents the coupling relationship between tasks and risks. 2) The improved FAHP determines weights through a fuzzy consistency matrix and vector norm calculation, which reduces the limitations of traditional AHP and FAHP in terms of consistency testing, iterative computation, and result stability. It is therefore more suitable for multi-criteria evaluation problems with strong subjectivity and uncertainty, such as styling design tasks. 3) The VIKOR method balances group utility and individual regret, highlighting the impact of critical risk factors. It overcomes the limitation of fully compensatory methods such as TOPSIS, which may weaken the influence of veto type risks. As a result, the risk ranking and classification are more consistent with engineering practice in styling design. 4) The case study identifies the front air intake, front and rear bumpers, and lidar related surfaces as high-risk nodes, while medium risk nodes are mainly associated with components such as headlamps, body pillar surfaces, and wheels. Based on these results, the proposed hierarchical risk control strategy can be directly applied to project scheduling, resource allocation, and design review optimization.

However, several limitations remain. On the one hand, the quantification of risk occurrence probability and loss severity still relies on expert judgment. Although multiple rounds of evaluation and consistency adjustments are used to reduce subjectivity, there is still room for improvement in terms of data objectivity. On the other hand, the current model is based on a static risk assessment framework and does not fully capture the dynamic evolution of risks or their transmission across different design stages. In addition, differences in design processes, organizational structures, and risk preferences among enterprises may affect the general applicability of the model, and further validation is needed.

Future research can focus on integrating risk identification results with project scheduling. After identifying the risks of styling design tasks, the next step is to incorporate quantified risk results into schedule optimization. Specifically, risk weights and risk levels can be introduced into traditional methods such as the critical path method or resource constrained scheduling models. Time buffers and resource redundancy can be assigned to high-risk tasks, while medium risk tasks can be managed through dynamic monitoring and flexible adjustment. This would enable multi-objective optimization across time, resources, and risk. In addition, future work may explore dynamic scheduling mechanisms for multi stage iterative processes, linking risk evolution with design iteration cycles to support real time adjustment of task priorities.

## Supporting information

S1 File Code. This file contains the code used to implement the four methods applied in this study.
(DOCX)

S2 File Raw data. This file contains the initial WBS node classification and the original WBS-RBS risk matrix used in the study.
(DOCX)

S3 File Intermediate calculation data. This file contains the matrices generated during the calculation process, the final weight values, benefit ratios, and the risk levels of each WBS task node.
(DOCX)

## References

[1] Wu GJ. Rhythm and harmony in automotive styling design. Zhuangshi (Art & Design). 2005;(11):110–1.

[2] Ranscombe C, Kinsella P, Blijlevens J. Data-driven styling: augmenting intuition in the product design process using holistic styling analysis. Journal of Mechanical Design. 2017;139(11). doi: 10.1115/1.4037249

[3] Luos M, Yi P, Liu Y, Tang H, Zhang J, Shao J, et al. Automobile styling design process reengineering method driven by intelligent technology. Computer Integrated Manufacturing Systems. 2025;31(9):3113–22. doi: 10.13196/j.cims.2025.0330

[4] Ribeiro AS, deCastro M, Costoya X, Rusu L, Dias JM, Gomez-Gesteira M. A Delphi method to classify wave energy resource for the 21st century: Application to the NW Iberian Peninsula. Energy. 2021;235. doi: 10.1016/j.energy.2021.121396

[5] Larkin P, Leiss W, Krewski D. Risk assessment and management frameworks for carbon capture and geological storage: a global perspective. IJRAM. 2019;22(3/4):254. doi: 10.1504/ijram.2019.103332

[6] Yang R, Deng Y. Analysis on security risks in tunnel construction based on the fault tree analysis. IOP Conference Series: Earth and Environmental Science. 2021;638(1):012089. doi: 10.1088/1755-1315/638/1/012089

[7] Yousefi S, Alizadeh A, Hayati J, Baghery M. HSE risk prioritization using robust DEA-FMEA approach with undesirable outputs: a study of automotive parts industry in Iran. Safety Science. 2018;102:144–58. doi: 10.1016/j.ssci.2017.10.015

[8] Li BW, Lü K, Ji ZY. Estimation and optimization of project management risk factors based on the AHP-taking car parts project as an example. Science and Technology Management Research. 2016;36(2):218–23. doi: 10.3969/j.issn.1000-7695.2016.02.039

[9] Mzougui I, Carpitella S, Certa A, El Felsoufi Z, Izquierdo J. Assessing supply chain risks in the automotive industry through a modified MCDM-based FMECA. Processes. 2020;8(5):579. doi: 10.3390/pr8050579

[10] Hu MT. Risk identification and evaluation of automobile supply chain based on Bayesian network. Logistics Technology. 2023;42(8):137-142,148. doi: 10.3969/j.issn.1005-152X.2023.08.023

[11] Li YL, Pan CX, Cai Q. A risk evaluation study of car design development based on fuzzy analytic hierarchy process. Journal of Tongji University (Natural Science). 2023;51(11):1771–4.

[12] "Ju Q, Liu W, Ruan J. Evaluation of vehicle body styling based on AHP and EWM method. In: Proceedings-2021 3rd International Conference on Artificial Intelligence and Advanced Manufacture, AIAM 2021, 2021. p. 461–7. 10.1109/AIAM54119.2021.00098

[13] Xu H, Qiu Z, Xu S, Mao L, Cui Z. Research on Risk Assessment and Prevention-Control Measures for Immersed Tunnel Construction in 100 m-Deep Water Environments. Journal of Marine Science and Engineering. 2025;14(1):53. doi: 10.3390/jmse14010053

[14] Wang T, Chen H, Hamat B, Zhao Y. Research on cultural and creative design method of 2022 World Cup lamps based on AHP-FCE. PLoS One. 2023;18(11):e0286682. doi: 10.1371/journal.pone.0286682 37988342 PMC10662743

[15] Moktadir MdA, Paul SK, Bai C, Santibanez Gonzalez EDR. The current and future states of MCDM methods in sustainable supply chain risk assessment. Environ Dev Sustain. 2024;27(3):7435–80. doi: 10.1007/s10668-023-04200-1

[16] Zhang GF, Wang R. Risk Evaluation Model of Automotive Supplier Based on WBS-RBS. Journal of Wuhan University of Technology (Information & Management Engineering). 2015;37(6):803–7. doi: 10.3963/j.issn.2095-3852.2015.06.031

[17] Wu P, Lin F, Huang J, Xu Y. Multiscale evaluation of tunnel construction safety risk: a case study of an offshore tunnel construction in Ningbo. Journal of Engineering and Applied Science. 2024;71(1):108. doi: 10.1186/s44147-024-00441-7

[18] Zhou HB, Gao WJ, Cai LB, Zhang H. Risk identification and analysis of subway foundation pit by using fault tree analysis method based on WBS-RBS. Rock and Soil Mechanics. 2009;30(9):2703–7. doi: 10.16285/j.rsm.2009.09.012

[19] Xiang Y, Wu Q, Zhang T. The risk assessment of bridge design based on the AHP-FCE model. China Civil Engineering Journal. 2010;43:275–80. doi: 10.15951/j.tmgcxb.2010.s2.017

[20] Lei M, Wang YQ, Wu L. Research on the risk assessment of highway survey and design based on integrating AHP and cloud model. Highway. 2025;70(2):256–62.

[21] Hyun K-C, Min S, Choi H, Park J, Lee I-M. Risk analysis using fault-tree analysis (FTA) and analytic hierarchy process (AHP) applicable to shield TBM tunnels. Tunnelling and Underground Space Technology. 2015;49:121–9. doi: 10.1016/j.tust.2015.04.007

[22] "Gheorghe A. Risk assessment of oil and natural gas drilling process by employing fuzzy sets and analytical hierarchy process (AHP). In: 2017 International Annual Conference of the American Society for Engineering Management, ASEM 2017, 2017.

[23] Chen GH, Wu WS, Xu SY, Liu K. Assessment HSE risk of during sea-crossing bridges project construction based on WBS-RBS and AHP. China Safety Science Journal. 2013;23(9):51–7. doi: 10.16265/j.cnki.issn1003-3033.2013.09.003

[24] Dong S, Li S, Yu F, Wang K. Risk Assessment of Immersed Tube Tunnel Construction. Processes. 2023;11(4):980. doi: 10.3390/pr11040980

[25] Chen HJ, Liu B, Ren X. Key risks identification of management informatization projects construction for coal enterprises based on improved WBS-RBS and FHAP method. China Coal. 2015;41(6):30–7. doi: 10.19880/j.cnki.ccm.2015.06.007

[26] Ji T, Liu JW, Li QF. Safety risk evaluation of large and complex bridges during construction based on the Delphi-improved FAHP-factor analysis method. Advances in Civil Engineering. 2022;2022:5397032. doi: 10.1155/2022/5397032

[27] Han M, Liu W, Zhao J, Du Y, Zhang L. Risk assessment of floor water inrush using random forest, VIKOR, and GIS: a case study. Geomechanics & Engineering. 2025;40(1):59–68. doi: 10.12989/gae.2025.40.1.059

[28] Jianxing Y, Shibo W, Haicheng C, Yang Y, Haizhao F, Jiahao L. Risk assessment of submarine pipelines using modified FMEA approach based on cloud model and extended VIKOR method. Process Safety and Environmental Protection. 2021;155:555–74. doi: 10.1016/j.psep.2021.09.047

[29] Wang H-L, Liu Y, Jiang Y, Wang Z-T. A novel approach for risk assessment of building damage via metro tunnel construction. Advances in Civil Engineering. 2022;2022(1). doi: 10.1155/2022/8448223

[30] Liu X, Lyu HM, Shen SL. Assessment of geo-disaster risk levels induced by extreme rainfall using integrated FCM-VIKOR approach. Georisk: Assessment and Management of Risk for Engineered Systems and Geohazards. 2025;19(4):755–74. doi: 10.1080/17499518.2025.2503254

[31] Yadav S, Pathak VK, Gangwar S. A novel hybrid TOPSIS-PSI approach for material selection in marine applications. Sādhanā. 2019;44(3). doi: 10.1007/s12046-018-1020-x

[32] Gangwar S, Yadav S, Pathak VK. Optimized selection of nanohydroxyapatite‐filled dental restorative composites formulation for best physico‐mechanical, chemical, and thermal properties using hybrid analytical hierarchy process‐multi‐objective optimization on the basis of ratio analysis approach. Polymer Composites. 2021;42(8):3841–56. doi: 10.1002/pc.26097

[33] Lyu H-M, Yin Z-Y, Zhou A, Shen S-L. MCDM-based flood risk assessment of metro systems in smart city development: a review. Environmental Impact Assessment Review. 2023;101:107154. doi: 10.1016/j.eiar.2023.107154

[34] Mir S, Shah SA, Bhat MS, Akhter S, Ahad F, Rashid H, et al. Disaster risk assessment of educational infrastructure in mountain geographies using PROMETHEE-II. International Journal of Disaster Risk Reduction. 2024;107:104489. doi: 10.1016/j.ijdrr.2024.104489

[35] Tran NT, Trinh VL, Chung CK. An integrated approach of fuzzy ahp-topsis for multi-criteria decision-making in industrial robot selection. Processes. 2024;12(8):1723. doi: 10.3390/pr12081723

[36] "Singh VK, Kumar R, Arya MK. Automotive product development lifecycle optimization through value engineering and value analysis (VAVE) techniques. In: 2017 International Conference on Advances in Mechanical, Industrial, Automation and Management Systems (AMIAMS), 2017. p. 349–54. 10.1109/amiams.2017.8069236

[37] Kubota FI, Cauchick-Miguel PA, Hsuan J, Lacerda DP. Modularity in design and production relationships: a field study in two automakers. International Journal of Advanced Manufacturing Technology. 2022;123(5–6):1589–606. doi: 10.1007/s00170-022-10262-8

[38] Qin Y. Automobile headlamp modeling design analysis based on brand design. Packaging Engineering. 2015;36(18):87–91. doi: 10.19554/j.cnki.1001-3563.2015.18.020

[39] Fu T, Li X, Xu W, Wang J, Zhang L, Ye L, et al. Risk analysis of bridge maintenance accidents: a two-stage LEC method and Bayesian network approach. International Journal of Transportation Science and Technology. 2024;15:51–64. doi: 10.1016/j.ijtst.2023.07.002

[40] Zhao Y, Liu Y. Research on subjective evaluation indexes for vehicle handling stability based on improved analytic hierarchy process and fuzzy comprehensive evaluation. China Mechanical Engineering. 2013;24(18):2519. doi: 10.3969/j.issn.1004-132X.2013.18.021

[41] Taherdoost H, Madanchian M. VIKOR method—an effective compromising ranking technique for decision making. 2023;:27–33. doi: 10.30564/mmpp.v5i2.5578

[42] Opricovic S, Tzeng G-H. Compromise solution by MCDM methods: A comparative analysis of VIKOR and TOPSIS. European Journal of Operational Research. 2004;156(2):445–55. doi: 10.1016/s0377-2217(03)00020-1

[43] Taherdoost H. Using PROMETHEE method for multi-criteria decision making: applications and procedures. IJEBM. 2023;1(1). doi: 10.33552/ijebm.2023.01.000502

[44] Mehrparvar M, Majak J, Karjust K. A comparative analysis of Fuzzy AHP and Fuzzy VIKOR methods for prioritization of the risk criteria of an autonomous vehicle system. PEAS. 2024;73(2):116–23. doi: 10.3176/proc.2024.2.04

[45] Li HM, Qu JJ, Zhang QQ. Geological suitability evaluation of underground space based on FAHP-TOPSIS method. Science Technology and Engineering. 2025;25(30):13187–96. doi: 10.12404/j.issn.1671-1815.2408407

