# Supplementary material for: Risk identification method for automotive styling design tasks based on an improved FAHP-VIKOR approach
Source: PLoS One. 2026 Jul 6;21(7):e0352278. doi: 10.1371/journal.pone.0352278 (PMC13336177; doi:10.1371/journal.pone.0352278)
Supplement: S1 File — This file contains the code used to implement the four methods applied in this study. (DOCX) [file pone.0352278.s001.docx]

**the improved FAHP-VIKOR method:**

import numpy as np

import pandas as pd

from typing import List, Dict, Union, Callable, Optional, Tuple

# ============================================================

# Improved FAHP: compute weights from pairwise matrix (power method)

# optionally return intermediate matrices

# ============================================================

def fahp_from_pairwise(A: np.ndarray, eps: float = 1e-4, max_iter: int = 100,

verbose: bool = False,

return_matrices: bool = False) -> Union[np.ndarray, Tuple[np.ndarray, dict]]:

"""

Input reciprocal matrix A, return normalized weights.

If return_matrices=True, returns (weights, matrices_dict) where matrices_dict contains A, F, E, etc.

"""

A = np.array(A, dtype=float)

n = A.shape[0]

if verbose:

print("\n====== Fuzzy pairwise matrix A ======")

print(pd.DataFrame(A))

# Construct consistent matrix F

r = np.sum(A, axis=1)

F = np.zeros((n, n))

for i in range(n):

for j in range(n):

F[i, j] = (r[i] - r[j]) / (2 * n) + 0.5

if verbose:

print("\n====== Consistency matrix F ======")

print(pd.DataFrame(F))

# Row sum normalization for initial weights

w0 = np.sum(F, axis=1)

w0 = w0 / np.sum(w0)

if verbose:

print("\n====== Initial weights w(0) ======")

print(w0)

# Construct reciprocal matrix E

E = F / F.T

if verbose:

print("\n====== Reciprocal matrix E ======")

print(pd.DataFrame(E))

# Power iteration

v = w0.copy()

for k in range(max_iter):

v_next = E @ v

v_next = v_next / np.linalg.norm(v_next, ord=np.inf)

if verbose:

print(f"Iteration {k+1}: {v_next}")

if np.linalg.norm(v_next - v) < eps:

if verbose:

print("Converged")

break

v = v_next

weights = v_next / np.sum(v_next)

if verbose:

print("\n====== Final weights ======")

print(weights)

if return_matrices:

matrices = {

'A': A,

'F': F,

'E': E,

'w0': w0,

'weights': weights

}

return weights, matrices

else:

return weights

# ============================================================

# Build pairwise matrix from risk values (0-1 scale)

# ============================================================

def build_pairwise_from_values(values: List[float]) -> np.ndarray:

m = len(values)

A = np.zeros((m, m))

for i in range(m):

for j in range(m):

if values[i] > values[j]:

A[i, j] = 1.0

elif values[i] < values[j]:

A[i, j] = 0.0

else:

A[i, j] = 0.5

return A

# ============================================================

# Global weight calculation (optionally output intermediate matrices to Excel)

# ============================================================

def compute_global_weights(

risk_matrix: Union[np.ndarray, pd.DataFrame],

group_structure: List[Dict],

exclude_zero: bool = True,

agg_func: Callable = np.sum,

verbose: bool = False,

verbose_nodes: Optional[List[int]] = None,

output_intermediate_file: Optional[str] = None,

task_names: Optional[List[str]] = None,

factor_names: Optional[List[str]] = None

) -> np.ndarray:

"""

Compute global weights from risk matrix and group structure.

If output_intermediate_file is provided, detailed intermediate matrices for

nodes in verbose_nodes will be saved to an Excel file.

task_names: list of task node names, length n_tasks

factor_names: list of risk factor names, length n_factors

"""

if isinstance(risk_matrix, pd.DataFrame):

risk_matrix = risk_matrix.values

X = np.array(risk_matrix, dtype=float)

n_tasks, n_factors = X.shape

all_indices = []

for g in group_structure:

all_indices.extend(g['indices'])

if len(set(all_indices)) != n_factors:

raise ValueError("Group indices must cover all columns exactly once")

if task_names is None:

task_names = [f"Task{i}" for i in range(n_tasks)]

if factor_names is None:

factor_names = [f"Factor{i}" for i in range(n_factors)]

global_weights = np.zeros((n_tasks, n_factors))

writer = None

if output_intermediate_file and verbose and verbose_nodes:

writer = pd.ExcelWriter(output_intermediate_file, engine='openpyxl')

for t in range(n_tasks):

row = X[t, :]

node_verbose = verbose and (verbose_nodes is None or t in verbose_nodes)

if verbose:

print(f"\n========== Task node {task_names[t]} ==========")

# ----- 1. Compute category representative values and level-1 pairwise matrix -----

cat_vals = []

for g in group_structure:

indices = g['indices']

vals = row[indices]

if exclude_zero:

vals = vals[vals > 0]

cat_val = agg_func(vals) if len(vals) > 0 else 0.0

cat_vals.append(cat_val)

valid_cats = [i for i, v in enumerate(cat_vals) if v > 0]

if len(valid_cats) == 0:

continue

valid_cat_vals = [cat_vals[i] for i in valid_cats]

A_cat = build_pairwise_from_values(valid_cat_vals)

if node_verbose and writer is not None:

w_cat_valid, matrices_cat = fahp_from_pairwise(A_cat, verbose=node_verbose, return_matrices=True)

cat_names = [group_structure[i]['name'] for i in valid_cats]

sheet_prefix = f"{task_names[t]}_R"

pd.DataFrame(matrices_cat['A'], index=cat_names, columns=cat_names).round(4).to_excel(writer, sheet_name=f"{sheet_prefix}_A")

pd.DataFrame(matrices_cat['F'], index=cat_names, columns=cat_names).round(4).to_excel(writer, sheet_name=f"{sheet_prefix}_F")

pd.DataFrame(matrices_cat['E'], index=cat_names, columns=cat_names).round(4).to_excel(writer, sheet_name=f"{sheet_prefix}_E")

pd.Series(matrices_cat['weights'], index=cat_names, name='weight').round(4).to_excel(writer, sheet_name=f"{sheet_prefix}_weight")

else:

w_cat_valid = fahp_from_pairwise(A_cat, verbose=node_verbose)

w_cat_full = np.zeros(len(group_structure))

for idx, w in zip(valid_cats, w_cat_valid):

w_cat_full[idx] = w

if node_verbose:

print("Level-1 pairwise matrix (valid categories):")

print(pd.DataFrame(A_cat, index=valid_cats, columns=valid_cats))

print("Level-1 weights (by category order):", w_cat_full)

# ----- 2. For each category, compute local weights of sub-factors -----

for i_cat, g in enumerate(group_structure):

cat_weight = w_cat_full[i_cat]

if cat_weight == 0:

continue

indices = g['indices']

sub_vals = row[indices]

if exclude_zero:

non_zero_mask = sub_vals > 0

sub_vals_nonzero = sub_vals[non_zero_mask]

nonzero_pos = np.where(non_zero_mask)[0]

else:

sub_vals_nonzero = sub_vals

nonzero_pos = np.arange(len(indices))

if len(sub_vals_nonzero) == 0:

continue

elif len(sub_vals_nonzero) == 1:

sub_w_nonzero = np.array([1.0])

if node_verbose and writer is not None:

factor_name = factor_names[indices[nonzero_pos[0]]]

df_weight = pd.DataFrame({factor_name: [1.0]}, index=['weight']).T

df_weight.round(4).to_excel(writer, sheet_name=f"{task_names[t]}_R{i_cat+1}_weight")

else:

A_sub = build_pairwise_from_values(sub_vals_nonzero.tolist())

if node_verbose and writer is not None:

sub_w_nonzero, matrices_sub = fahp_from_pairwise(A_sub, verbose=node_verbose, return_matrices=True)

factor_names_sub = [factor_names[indices[pos]] for pos in nonzero_pos]

sheet_prefix = f"{task_names[t]}_R{i_cat+1}"

pd.DataFrame(matrices_sub['A'], index=factor_names_sub, columns=factor_names_sub).round(4).to_excel(writer, sheet_name=f"{sheet_prefix}_A")

pd.DataFrame(matrices_sub['F'], index=factor_names_sub, columns=factor_names_sub).round(4).to_excel(writer, sheet_name=f"{sheet_prefix}_F")

pd.DataFrame(matrices_sub['E'], index=factor_names_sub, columns=factor_names_sub).round(4).to_excel(writer, sheet_name=f"{sheet_prefix}_E")

pd.Series(matrices_sub['weights'], index=factor_names_sub, name='weight').round(4).to_excel(writer, sheet_name=f"{sheet_prefix}_weight")

else:

sub_w_nonzero = fahp_from_pairwise(A_sub, verbose=node_verbose)

if node_verbose:

print(f"\nCategory {i_cat+1} internal pairwise matrix:")

print(pd.DataFrame(A_sub, index=nonzero_pos, columns=nonzero_pos))

for pos, w_sub in zip(nonzero_pos, sub_w_nonzero):

global_idx = indices[pos]

global_weights[t, global_idx] = cat_weight * w_sub

if node_verbose:

print("Global weight vector (all factors):", global_weights[t, :])

if writer is not None:

writer.close()

print(f"\nIntermediate matrices saved to {output_intermediate_file}")

return global_weights

# ============================================================

# VIKOR risk ranking (smaller Q → larger rank, ties share rank and skip)

# ============================================================

def vikor_with_task_weights(risk_matrix, weights_matrix, v=0.5):

X = np.array(risk_matrix)

W = np.array(weights_matrix)

m, n = X.shape

min_vals = X.min(axis=0)

max_vals = X.max(axis=0)

ranges = max_vals - min_vals

Z = np.zeros_like(X, dtype=float)

for j in range(n):

if ranges[j] != 0:

Z[:, j] = (X[:, j] - min_vals[j]) / ranges[j]

max_z = Z.max(axis=0)

min_z = Z.min(axis=0)

diff_z = max_z - min_z

S = np.zeros(m)

R = np.zeros(m)

for i in range(m):

ratio_i = np.zeros(n)

for j in range(n):

if diff_z[j] != 0:

ratio_i[j] = (max_z[j] - Z[i, j]) / diff_z[j]

weighted_ratio_i = ratio_i * W[i, :]

S[i] = weighted_ratio_i.sum()

R[i] = weighted_ratio_i.max()

min_S, max_S = S.min(), S.max()

min_R, max_R = R.min(), R.max()

S_norm = (S - min_S) / (max_S - min_S) if max_S > min_S else np.zeros_like(S)

R_norm = (R - min_R) / (max_R - min_R) if max_R > min_R else np.zeros_like(R)

Q = v * S_norm + (1 - v) * R_norm

# Risk level classification

conditions = [Q < 0.3, (Q >= 0.3) & (Q < 0.7), Q >= 0.7]

choices = ['low risk', 'medium risk', 'high risk']

risk_level = np.select(conditions, choices, default='unknown')

# Ranking: smaller Q → larger rank, ties share rank and skip

rank_asc_dense = pd.Series(Q).rank(method='dense', ascending=True).astype(int)

rank = m - rank_asc_dense + 1

result = pd.DataFrame({

'S': S,

'R': R,

'Q': Q,

'risk level': risk_level,

'rank': rank

})

return result

# ============================================================

# Main program

# ============================================================

if __name__ == "__main__":

# 1. Read Excel, first column as row index

df = pd.read_excel('WBS-RBS risk matrix.xlsx', index_col=0)

# 2. Convert to numeric, fill NaN with 0

df_numeric = df.apply(pd.to_numeric, errors='coerce').fillna(0)

# 3. Remove all-zero columns

non_zero_cols = (df_numeric != 0).any(axis=0)

df_clean = df_numeric.loc[:, non_zero_cols]

# 4. Convert to matrix

risk_matrix = df_clean.values

task_names = df_clean.index.tolist()

factor_names = df_clean.columns.tolist()

# 5. Define group structure (adjust indices according to remaining columns)

group_structure = [

{'name': 'technical risk', 'indices': [0, 1, 2]},

{'name': 'schedule risk', 'indices': [3, 4, 5]},

{'name': 'cost risk', 'indices': [6]},

{'name': 'resource coordination risk', 'indices': [7, 8]},

{'name': 'external compliance risk', 'indices': [9, 10, 11]}

]

# 6. Compute global weights, output intermediate matrices for selected nodes (0,5,10)

global_weights = compute_global_weights(

risk_matrix,

group_structure,

exclude_zero=True,

verbose=True,

verbose_nodes=[0, 5, 10],

output_intermediate_file='intermediate_matrices.xlsx',

task_names=task_names,

factor_names=factor_names

)

# 7. VIKOR ranking

result = vikor_with_task_weights(risk_matrix, global_weights, v=0.5)

result.insert(0, 'WBS node', df_clean.index)

result[['S', 'R', 'Q']] = result[['S', 'R', 'Q']].round(4)

print("\nVIKOR results:")

print(result)

# 8. Save main result

result.to_excel('FAHP+VIKOR_ranking.xlsx', index=False)

# 9. Save global weight matrix (with WBS node, 4 decimals)

weight_df = pd.DataFrame(global_weights, index=df_clean.index, columns=df_clean.columns)

weight_df_rounded = weight_df.round(4)

weight_df_reset = weight_df_rounded.reset_index().rename(columns={'index': 'WBS node'})

weight_df_reset.to_excel('nodes_weights.xlsx', index=False)

print("\nAll results saved.")

**the single FAHP weighted evaluation method:**

import numpy as np

import pandas as pd

from typing import List, Dict, Union, Callable, Optional

# ============================================================

# Helper function: map ratio to 1-9 scale

# ============================================================

def _ratio_to_scale(ratio: float) -> float:

"""Map ratio to 1-9 scale value (ensuring reciprocity)"""

if np.isclose(ratio, 1.0):

return 1.0

if ratio > 1:

if ratio < 1.3:

return 1

elif ratio < 1.6:

return 2

elif ratio < 1.9:

return 3

elif ratio < 2.2:

return 4

elif ratio < 2.5:

return 5

elif ratio < 2.8:

return 6

elif ratio < 3.1:

return 7

elif ratio < 3.4:

return 8

else:

return 9

else:

# ratio < 1, take reciprocal, map, then invert again

inv = 1.0 / ratio

if inv < 1.3:

return 1.0 / 1

elif inv < 1.6:

return 1.0 / 2

elif inv < 1.9:

return 1.0 / 3

elif inv < 2.2:

return 1.0 / 4

elif inv < 2.5:

return 1.0 / 5

elif inv < 2.8:

return 1.0 / 6

elif inv < 3.1:

return 1.0 / 7

elif inv < 3.4:

return 1.0 / 8

else:

return 1.0 / 9

# ============================================================

# Pairwise matrix constructor (1-9 scale)

# ============================================================

def build_pairwise_from_values(values: List[float]) -> np.ndarray:

"""

Construct reciprocal pairwise matrix using 1-9 scale.

If values[i] > values[j], then a_ij > 1, otherwise its reciprocal.

"""

m = len(values)

A = np.zeros((m, m))

for i in range(m):

for j in range(m):

if i == j:

A[i, j] = 1.0

else:

ratio = values[i] / values[j]

A[i, j] = _ratio_to_scale(ratio)

return A

# ============================================================

# Traditional AHP weight calculation and consistency check

# ============================================================

def ahp_compute_weights(A: np.ndarray, verbose: bool = True) -> np.ndarray:

"""

Input reciprocal matrix A, return normalized eigenvector weights.

If verbose, print consistency check results.

"""

n = A.shape[0]

# Compute eigenvalues and eigenvectors

eig_vals, eig_vecs = np.linalg.eig(A)

# Take the eigenvector corresponding to the largest real eigenvalue

max_idx = np.argmax(eig_vals.real)

lambda_max = eig_vals[max_idx].real

w = eig_vecs[:, max_idx].real

w = w / np.sum(w) # normalize

# Consistency check

if n > 2:

CI = (lambda_max - n) / (n - 1)

# RI table (consistent with paper)

RI_dict = {1: 0.12, 2: 0.35, 3: 0.56, 4: 0.82, 5: 1.21,

6: 1.36, 7: 1.43, 8: 1.45, 9: 1.49, 10: 1.50}

RI = RI_dict.get(n, 1.50) # for >10 use 1.50 approx

CR = CI / RI

if verbose:

print(f"λ_max = {lambda_max:.4f}, CI = {CI:.4f}, RI = {RI}, CR = {CR:.4f}")

if CR < 0.1:

print("Consistency check passed (CR < 0.1)")

else:

print("Consistency check failed (CR >= 0.1), consider adjusting the matrix")

else:

CR = 0.0

if verbose:

print("n <= 2, no consistency check needed")

return w

# ============================================================

# Global weight calculation (using traditional AHP)

# ============================================================

def compute_global_weights(

risk_matrix: Union[np.ndarray, pd.DataFrame],

group_structure: List[Dict],

exclude_zero: bool = True,

agg_func: Callable = np.sum,

verbose: bool = True

) -> np.ndarray:

"""

Compute global weights from risk matrix and group structure using traditional AHP.

group_structure: each element is a dict {'indices': list of column indices}

"""

if isinstance(risk_matrix, pd.DataFrame):

risk_matrix = risk_matrix.values

X = np.array(risk_matrix, dtype=float)

n_tasks, n_factors = X.shape

all_indices = []

for g in group_structure:

all_indices.extend(g['indices'])

if len(set(all_indices)) != n_factors:

raise ValueError("Group indices must cover all columns exactly once")

global_weights = np.zeros((n_tasks, n_factors))

for t in range(n_tasks):

row = X[t, :]

if verbose:

print(f"\n========== Task node {t} ==========")

# ----- 1. Compute category representative values and level-1 pairwise matrix -----

cat_vals = []

for g in group_structure:

indices = g['indices']

vals = row[indices]

if exclude_zero:

vals = vals[vals > 0]

cat_val = agg_func(vals) if len(vals) > 0 else 0.0

cat_vals.append(cat_val)

valid_cats = [i for i, v in enumerate(cat_vals) if v > 0]

if len(valid_cats) == 0:

continue

valid_cat_vals = [cat_vals[i] for i in valid_cats]

A_cat = build_pairwise_from_values(valid_cat_vals)

if verbose:

print("Level-1 pairwise matrix (valid categories):")

print(pd.DataFrame(A_cat, index=valid_cats, columns=valid_cats))

w_cat_valid = ahp_compute_weights(A_cat, verbose=verbose)

w_cat_full = np.zeros(len(group_structure))

for idx, w in zip(valid_cats, w_cat_valid):

w_cat_full[idx] = w

if verbose:

print("Level-1 weights (by category order):", w_cat_full)

# ----- 2. For each category, compute local weights of sub-factors -----

for i_cat, g in enumerate(group_structure):

cat_weight = w_cat_full[i_cat]

if cat_weight == 0:

continue

indices = g['indices']

sub_vals = row[indices]

if exclude_zero:

non_zero_mask = sub_vals > 0

sub_vals_nonzero = sub_vals[non_zero_mask]

nonzero_pos = np.where(non_zero_mask)[0]

else:

sub_vals_nonzero = sub_vals

nonzero_pos = np.arange(len(indices))

if len(sub_vals_nonzero) == 0:

continue

elif len(sub_vals_nonzero) == 1:

sub_w_nonzero = np.array([1.0])

else:

A_sub = build_pairwise_from_values(sub_vals_nonzero.tolist())

if verbose:

print(f"\nCategory {i_cat} internal pairwise matrix:")

print(pd.DataFrame(A_sub, index=nonzero_pos, columns=nonzero_pos))

sub_w_nonzero = ahp_compute_weights(A_sub, verbose=verbose)

for pos, w_sub in zip(nonzero_pos, sub_w_nonzero):

global_idx = indices[pos]

global_weights[t, global_idx] = cat_weight * w_sub

if verbose:

print("Global weight vector (all factors):", global_weights[t, :])

return global_weights

def compute_task_risk(risk_matrix, global_weights):

# Weighted sum

risk_values = np.sum(risk_matrix * global_weights, axis=1)

return risk_values

def classify_risk(value):

if value > 25:

return "high risk"

elif value >= 9:

return "medium risk"

else:

return "low risk"

# ============================================================

# Example usage

# ============================================================

if __name__ == "__main__":

# 1. Read Excel, first column as row index

df = pd.read_excel('WBS-RBS risk matrix.xlsx', index_col=0)

# 2. Convert to numeric, fill NaN with 0

df_numeric = df.apply(pd.to_numeric, errors='coerce').fillna(0)

# 3. Remove all-zero columns

non_zero_cols = (df_numeric != 0).any(axis=0)

df_clean = df_numeric.loc[:, non_zero_cols]

# 4. Convert to matrix

risk_matrix = df_clean.values

group_structure = [

{'name': 'technical risk', 'indices': [0, 1, 2]},

{'name': 'schedule risk', 'indices': [3, 4, 5]},

{'name': 'cost risk', 'indices': [6]},

{'name': 'resource coordination risk', 'indices': [7, 8]},

{'name': 'external compliance risk', 'indices': [9, 10, 11]}

]

# Compute global weights for each task node

global_weights = compute_global_weights(risk_matrix, group_structure,

exclude_zero=True, verbose=False)

risk_values = compute_task_risk(risk_matrix, global_weights)

risk_levels = [classify_risk(v) for v in risk_values]

# Compute rank (ascending: smaller risk value gets smaller rank number)

risk_series = pd.Series(risk_values)

ranks = risk_series.rank(method='min', ascending=True).astype(int)

# Build result table

result_df = pd.DataFrame({

'WBS node': df_clean.index,

'risk value': risk_values,

'rank': ranks,

'risk level': risk_levels

})

# Round risk values to 2 decimals

result_df[['risk value']] = result_df[['risk value']].round(2)

print(result_df)

# Save to Excel

result_df.to_excel('AHP_method.xlsx', index=False)

**the FAHP-TOPSIS method:**

import numpy as np

import pandas as pd

from typing import List, Dict, Union, Callable

# ============================================================

# Improved FAHP: compute weights from fuzzy pairwise matrix (power method)

# ============================================================

def fahp_from_pairwise(A: np.ndarray, eps: float = 1e-4, max_iter: int = 100,

verbose: bool = False) -> np.ndarray:

A = np.array(A, dtype=float)

n = A.shape[0]

if verbose:

print("\n====== Fuzzy pairwise matrix A ======")

print(pd.DataFrame(A))

# Step 2: Construct consistency matrix F

r = np.sum(A, axis=1)

F = np.zeros((n, n))

for i in range(n):

for j in range(n):

F[i, j] = (r[i] - r[j]) / (2 * n) + 0.5

if verbose:

print("\n====== Consistency matrix F ======")

print(pd.DataFrame(F))

# Step 3: Row sum normalization for initial weights

w0 = np.sum(F, axis=1)

w0 = w0 / np.sum(w0)

if verbose:

print("\n====== Initial weights w(0) ======")

print(w0)

# Step 4: Construct reciprocal matrix E

E = F / F.T

if verbose:

print("\n====== Reciprocal matrix E ======")

print(pd.DataFrame(E))

# Step 5: Power iteration for final weights

v = w0.copy()

for k in range(max_iter):

v_next = E @ v

v_next = v_next / np.linalg.norm(v_next, ord=np.inf)

if verbose:

print(f"Iteration {k+1}: {v_next}")

if np.linalg.norm(v_next - v) < eps:

if verbose:

print("Converged")

break

v = v_next

weights = v_next / np.sum(v_next)

if verbose:

print("\n====== Final weights ======")

print(weights)

return weights

# ============================================================

# Automatically build fuzzy pairwise matrix and compute global weights

# ============================================================

def build_pairwise_from_values(values: List[float]) -> np.ndarray:

m = len(values)

A = np.zeros((m, m))

for i in range(m):

for j in range(m):

if values[i] > values[j]:

A[i, j] = 1.0

elif values[i] < values[j]:

A[i, j] = 0.0

else:

A[i, j] = 0.5

return A

def compute_global_weights(

risk_matrix: Union[np.ndarray, pd.DataFrame],

group_structure: List[Dict],

exclude_zero: bool = True,

agg_func: Callable = np.sum,

verbose: bool = False

) -> np.ndarray:

# Convert to numpy matrix

if isinstance(risk_matrix, pd.DataFrame):

risk_matrix = risk_matrix.values

X = np.array(risk_matrix, dtype=float)

n_tasks, n_factors = X.shape

# Check grouping validity

all_indices = []

for g in group_structure:

all_indices.extend(g['indices'])

if len(set(all_indices)) != n_factors:

raise ValueError("Group indices must cover all columns exactly once")

# Result container

global_weights = np.zeros((n_tasks, n_factors))

# Iterate over each task node

for t in range(n_tasks):

row = X[t, :]

if verbose:

print(f"\n========== Task node {t} ==========")

# ----- 1. Compute category representative values and level-1 pairwise matrix -----

cat_vals = []

for g in group_structure:

indices = g['indices']

vals = row[indices]

if exclude_zero:

vals = vals[vals > 0]

cat_val = agg_func(vals) if len(vals) > 0 else 0.0

cat_vals.append(cat_val)

# Build level-1 pairwise matrix (only categories with representative value > 0)

valid_cats = [i for i, v in enumerate(cat_vals) if v > 0]

if len(valid_cats) == 0:

# All category representative values are 0, node has no risk, weights all zero

continue

valid_cat_vals = [cat_vals[i] for i in valid_cats]

A_cat = build_pairwise_from_values(valid_cat_vals)

w_cat_valid = fahp_from_pairwise(A_cat, verbose=verbose)

# Map valid category weights back to full category list (non-participating categories get weight 0)

w_cat_full = np.zeros(len(group_structure))

for idx, w in zip(valid_cats, w_cat_valid):

w_cat_full[idx] = w

if verbose:

print("Level-1 weights (by category order):", w_cat_full)

# ----- 2. For each category, compute local weights of sub-factors -----

for i_cat, g in enumerate(group_structure):

cat_weight = w_cat_full[i_cat]

if cat_weight == 0:

# Category weight is zero, its internal factors also get zero weight

continue

indices = g['indices']

sub_vals = row[indices]

if exclude_zero:

non_zero_mask = sub_vals > 0

sub_vals_nonzero = sub_vals[non_zero_mask]

nonzero_pos = np.where(non_zero_mask)[0] # positions within the group

else:

sub_vals_nonzero = sub_vals

nonzero_pos = np.arange(len(indices))

if len(sub_vals_nonzero) == 0:

# All factors in this category are zero, no weight

continue

elif len(sub_vals_nonzero) == 1:

# Only one factor, local weight = 1

sub_w_nonzero = np.array([1.0])

else:

# Build intra-category pairwise matrix

A_sub = build_pairwise_from_values(sub_vals_nonzero.tolist())

sub_w_nonzero = fahp_from_pairwise(A_sub, verbose=verbose)

# Map local weights back to original factor positions

for pos, w_sub in zip(nonzero_pos, sub_w_nonzero):

global_idx = indices[pos] # factor index in total columns

global_weights[t, global_idx] = cat_weight * w_sub

if verbose:

print("Global weight vector (all factors):", global_weights[t, :])

return global_weights

def topsis(risk_matrix: np.ndarray, weights: np.ndarray, task_names=None) -> pd.DataFrame:

# 1. Normalize risk matrix (column normalization)

col_norms = np.sqrt(np.sum(risk_matrix ** 2, axis=0))

col_norms[col_norms == 0] = 1

normalized = risk_matrix / col_norms

# 2. Build weighted normalized matrix

weighted = normalized * weights

# 3. Positive and negative ideal solutions

ideal_best = np.max(weighted, axis=0)

ideal_worst = np.min(weighted, axis=0)

# 4. Distance calculation

dist_best = np.sqrt(np.sum((weighted - ideal_best) ** 2, axis=1))

dist_worst = np.sqrt(np.sum((weighted - ideal_worst) ** 2, axis=1))

# 5. Relative closeness

with np.errstate(divide='ignore', invalid='ignore'):

closeness = dist_worst / (dist_best + dist_worst)

closeness[np.isnan(closeness)] = 0

# 6. Ranking: smaller closeness → smaller rank number (ascending)

rank = pd.Series(closeness).rank(method='min', ascending=True).astype(int).values

# 7. Risk level classification

def risk_level(c):

if c <= 0.3:

return "low risk"

elif c <= 0.7:

return "medium risk"

else:

return "high risk"

risk_levels = [risk_level(c) for c in closeness]

# Build result DataFrame

result = pd.DataFrame({

'WBS node': task_names,

'closeness': closeness,

'rank': rank,

'risk level': risk_levels

})

return result

# ============================================================

# Example usage

# ============================================================

if __name__ == "__main__":

# 1. Read Excel, first column as row index

df = pd.read_excel('WBS-RBS风险矩阵.xlsx', index_col=0)

# 2. Convert to numeric, fill NaN with 0

df_numeric = df.apply(pd.to_numeric, errors='coerce').fillna(0)

# 3. Remove all-zero columns

non_zero_cols = (df_numeric != 0).any(axis=0)

df_clean = df_numeric.loc[:, non_zero_cols]

# 4. Convert to matrix

risk_matrix = df_clean.values

group_structure = [

{'name': 'technical risk', 'indices': [0, 1, 2]},

{'name': 'schedule risk', 'indices': [3, 4, 5]},

{'name': 'cost risk', 'indices': [6]},

{'name': 'resource coordination risk', 'indices': [7, 8]},

{'name': 'external compliance risk', 'indices': [9, 10, 11]}

]

# Compute global weights for each task node

global_weights = compute_global_weights(risk_matrix, group_structure,

exclude_zero=True, verbose=False)

result = topsis(risk_matrix, global_weights, task_names=df_clean.index.tolist())

result[['closeness']] = result[['closeness']].round(4)

print("\nTOPSIS ranking results:")

print(result)

result.to_excel('FAHP+TOPSIS_ranking.xlsx', index=False

**the FAHP-VIKOR method:**

import numpy as np

import pandas as pd

from typing import List, Dict, Union, Callable, Optional

# ============================================================

# Helper function: map ratio to 1-9 scale

# ============================================================

def _ratio_to_scale(ratio: float) -> float:

"""Map ratio to 1-9 scale value (ensuring reciprocity)"""

if np.isclose(ratio, 1.0):

return 1.0

if ratio > 1:

if ratio < 1.3:

return 1

elif ratio < 1.6:

return 2

elif ratio < 1.9:

return 3

elif ratio < 2.2:

return 4

elif ratio < 2.5:

return 5

elif ratio < 2.8:

return 6

elif ratio < 3.1:

return 7

elif ratio < 3.4:

return 8

else:

return 9

else:

inv = 1.0 / ratio

if inv < 1.3:

return 1.0 / 1

elif inv < 1.6:

return 1.0 / 2

elif inv < 1.9:

return 1.0 / 3

elif inv < 2.2:

return 1.0 / 4

elif inv < 2.5:

return 1.0 / 5

elif inv < 2.8:

return 1.0 / 6

elif inv < 3.1:

return 1.0 / 7

elif inv < 3.4:

return 1.0 / 8

else:

return 1.0 / 9

# ============================================================

# Pairwise matrix constructor (1-9 scale)

# ============================================================

def build_pairwise_from_values(values: List[float]) -> np.ndarray:

m = len(values)

A = np.zeros((m, m))

for i in range(m):

for j in range(m):

if i == j:

A[i, j] = 1.0

else:

ratio = values[i] / values[j]

A[i, j] = _ratio_to_scale(ratio)

return A

# ============================================================

# Traditional AHP weight calculation and consistency check

# ============================================================

def ahp_compute_weights(A: np.ndarray, verbose: bool = True) -> np.ndarray:

n = A.shape[0]

eig_vals, eig_vecs = np.linalg.eig(A)

max_idx = np.argmax(eig_vals.real)

lambda_max = eig_vals[max_idx].real

w = eig_vecs[:, max_idx].real

w = w / np.sum(w)

if n > 2:

CI = (lambda_max - n) / (n - 1)

RI_dict = {1: 0.12, 2: 0.35, 3: 0.56, 4: 0.82, 5: 1.21,

6: 1.36, 7: 1.43, 8: 1.45, 9: 1.49, 10: 1.50}

RI = RI_dict.get(n, 1.50)

CR = CI / RI

if verbose:

print(f"λ_max = {lambda_max:.4f}, CI = {CI:.4f}, RI = {RI}, CR = {CR:.4f}")

if CR < 0.1:

print("Consistency check passed (CR < 0.1)")

else:

print("Consistency check failed (CR >= 0.1), consider adjusting the matrix")

else:

if verbose:

print("n <= 2, no consistency check needed")

return w

# ============================================================

# Global weight calculation (using traditional AHP)

# ============================================================

def compute_global_weights(

risk_matrix: Union[np.ndarray, pd.DataFrame],

group_structure: List[Dict],

exclude_zero: bool = True,

agg_func: Callable = np.sum,

verbose: bool = True

) -> np.ndarray:

if isinstance(risk_matrix, pd.DataFrame):

risk_matrix = risk_matrix.values

X = np.array(risk_matrix, dtype=float)

n_tasks, n_factors = X.shape

all_indices = []

for g in group_structure:

all_indices.extend(g['indices'])

if len(set(all_indices)) != n_factors:

raise ValueError("Group indices must cover all columns exactly once")

global_weights = np.zeros((n_tasks, n_factors))

for t in range(n_tasks):

row = X[t, :]

if verbose:

print(f"\n========== Task node {t} ==========")

cat_vals = []

for g in group_structure:

indices = g['indices']

vals = row[indices]

if exclude_zero:

vals = vals[vals > 0]

cat_val = agg_func(vals) if len(vals) > 0 else 0.0

cat_vals.append(cat_val)

valid_cats = [i for i, v in enumerate(cat_vals) if v > 0]

if len(valid_cats) == 0:

continue

valid_cat_vals = [cat_vals[i] for i in valid_cats]

A_cat = build_pairwise_from_values(valid_cat_vals)

if verbose:

print("Level-1 pairwise matrix (valid categories):")

print(pd.DataFrame(A_cat, index=valid_cats, columns=valid_cats))

w_cat_valid = ahp_compute_weights(A_cat, verbose=verbose)

w_cat_full = np.zeros(len(group_structure))

for idx, w in zip(valid_cats, w_cat_valid):

w_cat_full[idx] = w

if verbose:

print("Level-1 weights (by category order):", w_cat_full)

for i_cat, g in enumerate(group_structure):

cat_weight = w_cat_full[i_cat]

if cat_weight == 0:

continue

indices = g['indices']

sub_vals = row[indices]

if exclude_zero:

non_zero_mask = sub_vals > 0

sub_vals_nonzero = sub_vals[non_zero_mask]

nonzero_pos = np.where(non_zero_mask)[0]

else:

sub_vals_nonzero = sub_vals

nonzero_pos = np.arange(len(indices))

if len(sub_vals_nonzero) == 0:

continue

elif len(sub_vals_nonzero) == 1:

sub_w_nonzero = np.array([1.0])

else:

A_sub = build_pairwise_from_values(sub_vals_nonzero.tolist())

if verbose:

print(f"\nCategory {i_cat} internal pairwise matrix:")

print(pd.DataFrame(A_sub, index=nonzero_pos, columns=nonzero_pos))

sub_w_nonzero = ahp_compute_weights(A_sub, verbose=verbose)

for pos, w_sub in zip(nonzero_pos, sub_w_nonzero):

global_idx = indices[pos]

global_weights[t, global_idx] = cat_weight * w_sub

if verbose:

print("Global weight vector (all factors):", global_weights[t, :])

return global_weights

# ============================================================

# VIKOR risk ranking

# ============================================================

def vikor_with_task_weights(risk_matrix, weights_matrix, v=0.5):

X = np.array(risk_matrix)

W = np.array(weights_matrix)

m, n = X.shape

min_vals = X.min(axis=0)

max_vals = X.max(axis=0)

ranges = max_vals - min_vals

Z = np.zeros_like(X, dtype=float)

for j in range(n):

if ranges[j] != 0:

Z[:, j] = (X[:, j] - min_vals[j]) / ranges[j]

max_z = Z.max(axis=0)

min_z = Z.min(axis=0)

diff_z = max_z - min_z

S = np.zeros(m)

R = np.zeros(m)

for i in range(m):

ratio_i = np.zeros(n)

for j in range(n):

if diff_z[j] != 0:

ratio_i[j] = (max_z[j] - Z[i, j]) / diff_z[j]

weighted_ratio_i = ratio_i * W[i, :]

S[i] = weighted_ratio_i.sum()

R[i] = weighted_ratio_i.max()

min_S, max_S = S.min(), S.max()

min_R, max_R = R.min(), R.max()

S_norm = (S - min_S) / (max_S - min_S) if max_S > min_S else np.zeros_like(S)

R_norm = (R - min_R) / (max_R - min_R) if max_R > min_R else np.zeros_like(R)

Q = v * S_norm + (1 - v) * R_norm

# Risk level classification (smaller Q means lower risk)

conditions = [Q < 0.3, (Q >= 0.3) & (Q < 0.7), Q >= 0.7]

choices = ['high risk', 'medium risk', 'low risk']

risk_level = np.select(conditions, choices, default='unknown')

# Ranking: larger Q gets smaller rank number (i.e., higher risk gets rank 1)

# Use ascending=False so that the largest Q gets rank 1

rank = pd.Series(Q).rank(method='min', ascending=False).astype(int)

result = pd.DataFrame({

'S': S,

'R': R,

'Q': Q,

'risk level': risk_level,

'rank': rank

})

return result

# ============================================================

# Main program

# ============================================================

if __name__ == "__main__":

# 1. Read Excel, first column as row index

df = pd.read_excel('WBS-RBS risk matrix.xlsx', index_col=0)

# 2. Convert to numeric, fill NaN with 0

df_numeric = df.apply(pd.to_numeric, errors='coerce').fillna(0)

# 3. Remove all-zero columns

non_zero_cols = (df_numeric != 0).any(axis=0)

df_clean = df_numeric.loc[:, non_zero_cols]

# 4. Convert to matrix

risk_matrix = df_clean.values

# 5. Define group structure (adjust indices according to remaining columns)

group_structure = [

{'name': 'technical risk', 'indices': [0, 1, 2]},

{'name': 'schedule risk', 'indices': [3, 4, 5]},

{'name': 'cost risk', 'indices': [6]},

{'name': 'resource coordination risk', 'indices': [7, 8]},

{'name': 'external compliance risk', 'indices': [9, 10, 11]}

]

# 6. Compute global weights for each task node (FAHP)

global_weights = compute_global_weights(risk_matrix, group_structure,

exclude_zero=True, verbose=False)

print(global_weights)

# 7. Perform VIKOR risk ranking

result = vikor_with_task_weights(risk_matrix, global_weights, v=0.5)

# 8. Add WBS node column and keep four decimal places

result.insert(0, 'WBS node', df_clean.index)

result[['S', 'R', 'Q']] = result[['S', 'R', 'Q']].round(4)

print("\nVIKOR risk ranking results:")

print(result)

# 9. Save results

result.to_excel('FAHP+VIKOR_ranking.xlsx', index=False)

print("\nResults saved to FAHP+VIKOR_ranking.xlsx")
