# Supplementary material for: Risk identification method for automotive styling design tasks based on an improved FAHP-VIKOR approach
Source: PLoS One. 2026 Jul 6;21(7):e0352278. doi: 10.1371/journal.pone.0352278 (PMC13336177; doi:10.1371/journal.pone.0352278)
Supplement: S3 File — This file contains the matrices generated during the calculation process, the final weight values, benefit ratios, and the risk levels of each WBS task node. (DOCX) [file pone.0352278.s003.docx]

After completing the calculation steps of the improved FAHP–VIKOR method, the risk levels of the WBS nodes in the automotive styling design project can be obtained. The following presents the intermediate process matrices and weight data for nodes *W_11_, W_22_*, and *W_31_*, as well as the final Q values and the corresponding risk levels.

For node *W_11_*, the intermediate process matrices, weight data as follows.

Table 1. Fuzzy Judgement Matrix

| judgment matrix of W-R | judgment matrix of W-R_1_ | judgment matrix of W-R_2_ | judgment matrix of W-R_4_ | judgment matrix of W-R_5_ |
| --- | --- | --- | --- | --- |
| $\left[ \begin{matrix} \text{0.5} & \text{1.0} & \text{1.0} & \text{1.0} & \text{1.0} \\ \text{0.0} & \text{0.5} & \text{1.0} & \text{1.0} & \text{1.0} \\ \text{0.0} & \text{0.0} & \text{0.5} & \text{0.0} & \text{0.0} \\ \text{0.0} & \text{0.0} & \text{1.0} & \text{0.5} & \text{0.0} \\ \text{0.0} & \text{0.0} & \text{1.0} & \text{1.0} & \text{0.5} \end{matrix} \right]$ | $\left[ \begin{matrix} \text{0.5} & \text{1.0} & \text{1.0} \\ \text{0.0} & \text{0.5} & \text{1.0} \\ \text{0.0} & \text{0.0} & \text{0.5} \end{matrix} \right]$ | $\left[ \begin{matrix} \text{0.5} & \text{1.0} & \text{1.0} \\ \text{0.0} & \text{0.5} & \text{1.0} \\ \text{0.0} & \text{0.0} & \text{0.5} \end{matrix} \right]$ | $\left[ \begin{matrix} 0.5 & 0.0 \\ 1.0 & 0.5 \end{matrix} \right]$ | $\left[ \begin{matrix} \text{0.5} & \text{1.0} & \text{1.0} \\ \text{0.0} & \text{0.5} & \text{0.0} \\ \text{0.0} & \text{1.0} & \text{0.5} \end{matrix} \right]$ |

For node W-R, the corresponding consistency judgment matrix:

$$P=\left[ \begin{matrix} 0.5 & 0.6 & 0.9 & 0.8 & 0.7 \\ 0.4 & 0.5 & 0.8 & 0.7 & 0.6 \\ 0.1 & 0.2 & 0.5 & 0.4 & 0.3 \\ 0.2 & 0.3 & 0.6 & 0.5 & 0.4 \\ 0.3 & 0.4 & 0.7 & 0.6 & 0.5 \end{matrix} \right]$$

The initial weight vector is:

$$w^{（0）}=\left[ 0.28，0.24，0.12，0.16，0.20 \right]^{T}$$

The reciprocal judgment matrix is:

$$E=\left[ \begin{matrix} 1.0000 & 1.5000 & 9.0000 & 4.0000 & 2.3333 \\ 0.6667 & 1.0000 & 4.0000 & 2.3333 & 1.5000 \\ 0.1111 & 0.2500 & 1.0000 & 0.6667 & 0.4286 \\ 0.2500 & 0.4286 & 1.5000 & 1.0000 & 0.6667 \\ 0.4286 & 0.6667 & 2.3333 & 1.5000 & 1.0000 \end{matrix} \right]$$

With a convergence precision of 0.0001 and four iterations, the final weights obtained by iterative computation are:

$$w=\left[ 0.4253，0.2505，0.0613，0.1025，0.1604 \right]^{T}$$

For node *W_22_*, the intermediate process matrices, weight data as follows.

Table 2. Fuzzy Judgement Matrix

| judgment matrix of W-R | judgment matrix of W-R_1_ | judgment matrix of W-R_2_ | judgment matrix of W-R_4_ | judgment matrix of W-R_5_ |
| --- | --- | --- | --- | --- |
| $\left[ \begin{matrix} \text{0.5} & \text{1.0} & \text{1.0} & \text{1.0} & \text{1.0} \\ \text{0.0} & \text{0.5} & \text{1.0} & \text{1.0} & \text{1.0} \\ \text{0.0} & \text{0.0} & \text{0.5} & \text{0.0} & \text{0.0} \\ \text{0.0} & \text{0.0} & \text{1.0} & \text{0.5} & 0.5 \\ \text{0.0} & \text{0.0} & \text{1.0} & \text{0.5} & \text{0.5} \end{matrix} \right]$ | $\left[ \begin{matrix} \text{0.5} & \text{1.0} & \text{1.0} \\ \text{0.0} & \text{0.5} & \text{1.0} \\ \text{0.0} & \text{0.0} & \text{0.5} \end{matrix} \right]$ | $\left[ \begin{matrix} \text{0.5} & \text{1.0} & \text{1.0} \\ \text{0.0} & \text{0.5} & \text{1.0} \\ \text{0.0} & \text{0.0} & \text{0.5} \end{matrix} \right]$ | $\left[ \begin{matrix} 0.5 & 0.0 \\ 1.0 & 0.5 \end{matrix} \right]$ | $\left[ \begin{matrix} \text{0.5} & \text{1.0} & \text{1.0} \\ \text{0.0} & \text{0.5} & \text{0.0} \\ \text{0.0} & \text{1.0} & \text{0.5} \end{matrix} \right]$ |

For node W-R, the corresponding consistency judgment matrix:

$$P=\left[ \begin{matrix} 0.5 & 0.6 & 0.9 & 0.75 & 0.75 \\ 0.4 & 0.5 & 0.8 & 0.65 & 0.65 \\ 0.1 & 0.2 & 0.5 & 0.35 & 0.35 \\ 0.25 & 0.35 & 0.65 & 0.5 & 0.5 \\ 0.25 & 0.35 & 0.65 & 0.5 & 0.5 \end{matrix} \right]$$

The initial weight vector is:

$$w^{（0）}=\left[ 0.28，0.24，0.12，0.18，0.18 \right]^{T}$$

The reciprocal judgment matrix is:

$$E=\left[ \begin{matrix} 1.0000 & 1.5000 & 9.0000 & 3.0000 & 3.0000 \\ 0.6667 & 0.2500 & 4.0000 & 1.8571 & 1.8571 \\ 0.1111 & 0.2500 & 1.0000 & 0.5385 & 0.5385 \\ 0.3333 & 0.5385 & 1.8571 & 1.0000 & 1.0000 \\ 0.3333 & 0.5385 & 1.8571 & 1.0000 & 1.0000 \end{matrix} \right]$$

With a convergence precision of 0.0001 and four iterations, the final weights obtained by iterative computation are:

$$w=\left[ 0.4260，0.2520，0.0621，0.1299，0.1299 \right]^{T}$$

For node *W_31_*, the intermediate process matrices, weight data as follows.

Table 3. Fuzzy Judgement Matrix

| judgment matrix of W-R | judgment matrix of W-R_1_ | judgment matrix of W-R_2_ | judgment matrix of W-R_4_ | judgment matrix of W-R_5_ |
| --- | --- | --- | --- | --- |
| $\left[ \begin{matrix} \text{0.5} & \text{1.0} & \text{1.0} & \text{1.0} & \text{1.0} \\ \text{0.0} & \text{0.5} & \text{1.0} & \text{1.0} & \text{1.0} \\ \text{0.0} & \text{0.0} & \text{0.5} & \text{0.0} & \text{0.0} \\ \text{0.0} & \text{0.0} & \text{1.0} & \text{0.5} & 1\text{.0} \\ \text{0.0} & \text{0.0} & \text{1.0} & \text{0.0} & \text{0.5} \end{matrix} \right]$ | $\left[ \begin{matrix} \text{0.5} & \text{1.0} & \text{1.0} \\ \text{0.0} & \text{0.5} & \text{1.0} \\ \text{0.0} & \text{0.0} & \text{0.5} \end{matrix} \right]$ | $\left[ \begin{matrix} \text{0.5} & \text{1.0} & \text{1.0} \\ \text{0.0} & \text{0.5} & \text{1.0} \\ \text{0.0} & \text{0.0} & \text{0.5} \end{matrix} \right]$ | $\left[ \begin{matrix} 0.5 & 0.0 \\ 1.0 & 0.5 \end{matrix} \right]$ | $\left[ \begin{matrix} \text{0.5} & \text{1.0} & \text{1.0} \\ \text{0.0} & \text{0.5} & \text{0.0} \\ \text{0.0} & \text{1.0} & \text{0.5} \end{matrix} \right]$ |

For node W-R, the corresponding consistency judgment matrix:

$$P=\left[ \begin{matrix} 0.5 & 0.6 & 0.9 & 0.7 & 0.8 \\ 0.4 & 0.5 & 0.8 & 0.6 & 0.7 \\ 0.1 & 0.2 & 0.5 & 0.3 & 0.4 \\ 0.2 & 0.4 & 0.7 & 0.5 & 0.6 \\ 0.3 & 0.3 & 0.6 & 0.4 & 0.5 \end{matrix} \right]$$

The initial weight vector is:

$$w^{（0）}=\left[ 0.28，0.24，0.12，0.20，0.16 \right]^{T}$$

The reciprocal judgment matrix is:

$$E=\left[ \begin{matrix} 1.0000 & 1.5000 & 9.0000 & 2.3333 & 4.0000 \\ 0.6667 & 1.0000 & 4.0000 & 1.5000 & 3.3333 \\ 0.1111 & 0.2500 & 1.0000 & 0.4286 & 0.6667 \\ 0.4286 & 0.6667 & 2.3333 & 1.0000 & 1.5000 \\ 0.2500 & 0.4286 & 1.5000 & 0.6667 & 1.0000 \end{matrix} \right]$$

With a convergence precision of 0.0001 and four iterations, the final weights obtained by iterative computation are:

$$w=\left[ 0.4253，0.2505，0.0613，0.1604，0.1025 \right]^{T}$$

The following table presents the aggregated weights of the five major risk factors for each WBS node, calculated using the method described above. The final weights of the five risk factors for each WBS node are shown below.

Table 4. Weight Distribution of the Five Major Risk Factors

| *WBS node* | *R_1_* | *R_2_* | *R_3_* | *R_4_* | *R_5_* |
| --- | --- | --- | --- | --- | --- |
| *W_11_* | 0.4253 | 0.2505 | 0.0613 | 0.1025 | 0.0604 |
| *W_12_* | 0.4253 | 0.2505 | 0.0613 | 0.1025 | 0.0604 |
| *W_13_* | 0.4253 | 0.2505 | 0.0613 | 0.1025 | 0.0604 |
| *W_14_* | 0.4260 | 0.2520 | 0.0621 | 0.1299 | 0.1299 |
| *W_15_* | 0.4260 | 0.2520 | 0.0621 | 0.1299 | 0.1299 |
| *W_16_* | 0.4253 | 0.2505 | 0.0613 | 0.1025 | 0.0604 |
| *W_21_* | 0.4253 | 0.2505 | 0.0613 | 0.1025 | 0.0604 |
| *W_22_* | 0.4260 | 0.2520 | 0.0621 | 0.1299 | 0.1299 |
| *W_23_* | 0.4253 | 0.2505 | 0.0613 | 0.1025 | 0.0604 |
| *W_24_* | 0.4253 | 0.2505 | 0.0613 | 0.1604 | 0.1025 |
| *W_25_* | 0.4253 | 0.2505 | 0.0613 | 0.1604 | 0.1025 |
| *W_26_* | 0.4253 | 0.2505 | 0.0613 | 0.1025 | 0.0604 |
| *W_27_* | 0.4253 | 0.2505 | 0.0613 | 0.1604 | 0.1025 |
| *W_28_* | 0.4260 | 0.2520 | 0.0621 | 0.1299 | 0.1299 |
| *W_29_* | 0.4253 | 0.2505 | 0.0613 | 0.1604 | 0.1025 |
| *W_210_* | 0.4253 | 0.2505 | 0.0613 | 0.1025 | 0.0604 |
| *W_211_* | 0.4260 | 0.2520 | 0.0621 | 0.1299 | 0.1299 |
| *W_212_* | 0.4253 | 0.2505 | 0.0613 | 0.1025 | 0.0604 |
| *W_213_* | 0.4260 | 0.2520 | 0.0621 | 0.1299 | 0.1299 |
| *W_214_* | 0.4260 | 0.2520 | 0.0621 | 0.1299 | 0.1299 |
| *W_215_* | 0.4253 | 0.2505 | 0.0613 | 0.1025 | 0.0604 |
| *W_216_* | 0.4253 | 0.2505 | 0.0613 | 0.1025 | 0.0604 |
| *W_31_* | 0.4253 | 0.2505 | 0.0613 | 0.1604 | 0.1025 |
| *W_32_* | 0.4253 | 0.2505 | 0.0613 | 0.1025 | 0.0604 |
| *W_33_* | 0.4253 | 0.2505 | 0.0613 | 0.1025 | 0.0604 |
| *W_34_* | 0.4253 | 0.2505 | 0.0613 | 0.1025 | 0.0604 |
| *W_35_* | 0.4260 | 0.2520 | 0.0621 | 0.1299 | 0.1299 |

A sensitivity analysis was conducted to examine the influence of different risk factor weights on the ranking results. The results show that technical risk has the most significant impact, as small changes in its weight lead to noticeable variations in the ranking of several nodes, indicating its dominant role in the evaluation. Schedule risk exhibits a moderate influence, mainly affecting nodes with similar technical characteristics. In contrast, cost risk has minimal impact on the overall results, and most rankings remain stable under its variation. Resource and collaboration risk, as well as external and compliance risk, primarily affect specific nodes but do not significantly change the overall ranking structure. Overall, the model demonstrates good stability while remaining sensitive to critical risk factors, confirming the rationality of the weight distribution.

The weights of the remaining WBS nodes are calculated following the same procedures applied to the above nodes. The results are then summarized to form the WBS task weight node matrix, as shown below (see table 5).

Table 5. WBS task weight node matrix

| *WBS node* | *R_11_* | *R_12_* | *R_13_* | *R_21_* | *R_22_* | *R_23_* | *R_33_* | *R_41_* | *R_42_* | *R_51_* | *R_52_* | *R_53_* |
| --- | --- | --- | --- | --- | --- | --- | --- | --- | --- | --- | --- | --- |
| W_11_ | 0.2532 | 0.1175 | 0.0545 | 0.1491 | 0.0692 | 0.0321 | 0.0613 | 0.0256 | 0.0769 | 0.0955 | 0.0206 | 0.0443 |
| W_12_ | 0.2532 | 0.1175 | 0.0545 | 0.1491 | 0.0692 | 0.0321 | 0.0613 | 0.0256 | 0.0769 | 0.0955 | 0.0206 | 0.0443 |
| W_13_ | 0.2532 | 0.1175 | 0.0545 | 0.1491 | 0.0692 | 0.0321 | 0.0613 | 0.0256 | 0.0769 | 0.0955 | 0.0206 | 0.0443 |
| W_14_ | 0.2537 | 0.1178 | 0.0547 | 0.1500 | 0.0696 | 0.0323 | 0.0621 | 0.0325 | 0.0974 | 0.0773 | 0.0167 | 0.0359 |
| W_15_ | 0.2537 | 0.1178 | 0.0547 | 0.1500 | 0.0696 | 0.0323 | 0.0621 | 0.0325 | 0.0974 | 0.0773 | 0.0167 | 0.0359 |
| W_16_ | 0.2532 | 0.1175 | 0.0545 | 0.1491 | 0.0692 | 0.0321 | 0.0613 | 0.0256 | 0.0769 | 0.0955 | 0.0206 | 0.0443 |
| W_21_ | 0.2532 | 0.1175 | 0.0545 | 0.1491 | 0.0692 | 0.0321 | 0.0613 | 0.0256 | 0.0769 | 0.0955 | 0.0206 | 0.0443 |
| W_22_ | 0.2537 | 0.1178 | 0.0547 | 0.1500 | 0.0696 | 0.0323 | 0.0621 | 0.0325 | 0.0974 | 0.0773 | 0.0167 | 0.0359 |
| W_23_ | 0.2532 | 0.1175 | 0.0545 | 0.1491 | 0.0692 | 0.0321 | 0.0613 | 0.0256 | 0.0769 | 0.0955 | 0.0206 | 0.0443 |
| W_24_ | 0.2532 | 0.1175 | 0.0545 | 0.1491 | 0.0692 | 0.0321 | 0.0613 | 0.0401 | 0.1203 | 0.0610 | 0.0131 | 0.0283 |
| W_25_ | 0.2532 | 0.1175 | 0.0545 | 0.1491 | 0.0692 | 0.0321 | 0.0613 | 0.0401 | 0.1203 | 0.0610 | 0.0131 | 0.0283 |
| W_26_ | 0.2532 | 0.1175 | 0.0545 | 0.1491 | 0.0692 | 0.0321 | 0.0613 | 0.0256 | 0.0769 | 0.0955 | 0.0206 | 0.0443 |
| W_27_ | 0.2532 | 0.1175 | 0.0545 | 0.1491 | 0.0692 | 0.0321 | 0.0613 | 0.0401 | 0.1203 | 0.0610 | 0.0131 | 0.0283 |
| W_28_ | 0.2537 | 0.1178 | 0.0547 | 0.1500 | 0.0696 | 0.0323 | 0.0621 | 0.0325 | 0.0974 | 0.0773 | 0.0167 | 0.0359 |
| W_29_ | 0.2532 | 0.1175 | 0.0545 | 0.1491 | 0.0692 | 0.0321 | 0.0613 | 0.0401 | 0.1203 | 0.0610 | 0.0131 | 0.0283 |
| W_210_ | 0.2532 | 0.1175 | 0.0545 | 0.1491 | 0.0692 | 0.0321 | 0.0613 | 0.0256 | 0.0769 | 0.0955 | 0.0206 | 0.0443 |
| W_211_ | 0.2537 | 0.1178 | 0.0547 | 0.1500 | 0.0696 | 0.0323 | 0.0621 | 0.0325 | 0.0974 | 0.0773 | 0.0167 | 0.0359 |
| W_212_ | 0.2532 | 0.1175 | 0.0545 | 0.1491 | 0.0692 | 0.0321 | 0.0613 | 0.0256 | 0.0769 | 0.0955 | 0.0206 | 0.0443 |
| W_213_ | 0.2537 | 0.1178 | 0.0547 | 0.1500 | 0.0696 | 0.0323 | 0.0621 | 0.0325 | 0.0974 | 0.0773 | 0.0167 | 0.0359 |
| W_214_ | 0.2537 | 0.1178 | 0.0547 | 0.1500 | 0.0696 | 0.0323 | 0.0621 | 0.0325 | 0.0974 | 0.0779 | 0.0260 | 0.0260 |
| W_215_ | 0.2532 | 0.1175 | 0.0545 | 0.1491 | 0.0692 | 0.0321 | 0.0613 | 0.0256 | 0.0769 | 0.0955 | 0.0206 | 0.0443 |
| W_216_ | 0.2532 | 0.1175 | 0.0545 | 0.1491 | 0.0692 | 0.0321 | 0.0613 | 0.0256 | 0.0769 | 0.0955 | 0.0206 | 0.0443 |
| W_31_ | 0.2532 | 0.1175 | 0.0545 | 0.1491 | 0.0692 | 0.0321 | 0.0613 | 0.0401 | 0.1203 | 0.0610 | 0.0131 | 0.0283 |
| W_32_ | 0.2532 | 0.1175 | 0.0545 | 0.1491 | 0.0692 | 0.0321 | 0.0613 | 0.0256 | 0.0769 | 0.0955 | 0.0206 | 0.0443 |
| W_33_ | 0.2532 | 0.1175 | 0.0545 | 0.1491 | 0.0692 | 0.0321 | 0.0613 | 0.0256 | 0.0769 | 0.0955 | 0.0206 | 0.0443 |
| W_34_ | 0.2532 | 0.1175 | 0.0545 | 0.1491 | 0.0692 | 0.0321 | 0.0613 | 0.0256 | 0.0769 | 0.0955 | 0.0206 | 0.0443 |
| W_35_ | 0.2537 | 0.1178 | 0.0547 | 0.1500 | 0.0696 | 0.0323 | 0.0621 | 0.0325 | 0.0974 | 0.0773 | 0.0167 | 0.0359 |

Finally, based on the WBS task weight node matrix and the calculation procedure of the VIKOR method, the Q values of the WBS nodes are obtained to determine their corresponding risk levels, The results are summarized in Table 6.

Table 6. Summary of VIKOR results (risk-balanced type)

| WBS Node | S | R | Q | Rank | Risk Level |
| --- | --- | --- | --- | --- | --- |
| *W_11_* | 0.4513 | 0.0985 | 0.3781 | 22 | Medium Risk |
| *W_12_* | 0.2745 | 0.0486 | 0.1778 | 25 | High Risk |
| *W_13_* | 0.1263 | 0.0243 | 0.0483 | 26 | High Risk |
| *W_14_* | 0.3880 | 0.0663 | 0.2753 | 23 | High Risk |
| *W_15_* | 0.5855 | 0.1315 | 0.5195 | 20 | Medium Risk |
| *W_16_* | 0.8156 | 0.2016 | 0.7912 | 10 | Low Risk |
| *W_21_* | 0.5894 | 0.1547 | 0.5721 | 18 | Medium Risk |
| *W_22_* | 0.7865 | 0.2020 | 0.7770 | 11 | Low Risk |
| *W_23_* | 0.6926 | 0.1782 | 0.6765 | 13 | Medium Risk |
| *W_24_* | 0.8612 | 0.2110 | 0.8352 | 8 | Low Risk |
| *W_25_* | 0.9297 | 0.2251 | 0.9013 | 5 | Low Risk |
| *W_26_* | 0.2028 | 0.0657 | 0.1780 | 24 | High Risk |
| *W_27_* | 0.9437 | 0.2392 | 0.9392 | 3 | Low Risk |
| *W_28_* | 0.8798 | 0.2255 | 0.8764 | 6 | Low Risk |
| *W_29_* | 0.9500 | 0.2392 | 0.9424 | 2 | Low Risk |
| *W_210_* | 0.6926 | 0.1782 | 0.6765 | 15 | Medium Risk |
| *W_211_* | 0.7553 | 0.1832 | 0.7199 | 14 | Low Risk |
| *W_212_* | 0.6117 | 0.1641 | 0.6040 | 17 | Medium Risk |
| *W_213_* | 0.7865 | 0.2020 | 0.7770 | 11 | Low Risk |
| *W_214_* | 1.0000 | 0.2537 | 1.0000 | 1 | Low Risk |
| *W_215_* | 0.5553 | 0.1547 | 0.5545 | 19 | Medium Risk |
| *W_216_* | 0.8483 | 0.2110 | 0.8286 | 9 | Low Risk |
| *W_31_* | 0.9437 | 0.2392 | 0.9392 | 3 | Low Risk |
| *W_32_* | 0.4685 | 0.1313 | 0.4585 | 21 | Medium Risk |
| *W_33_* | 0.6926 | 0.1782 | 0.6765 | 15 | Medium Risk |
| *W_34_* | 0.0328 | 0.0328 | 0.0186 | 27 | High Risk |
| *W_35_* | 0.8798 | 0.2255 | 0.8764 | 6 | Low Risk |
