# Supplementary material for: Risk identification method for automotive styling design tasks based on an improved FAHP-VIKOR approach
Source: PLoS One. 2026 Jul 6;21(7):e0352278. doi: 10.1371/journal.pone.0352278 (PMC13336177; doi:10.1371/journal.pone.0352278)
Supplement: Attachment — Submitted filename: PONE-D-25-67607.pdf [file pone.0352278.s004.pdf]

PLOS One

Risk Identification Method for Automotive Styling Design Tasks Based on an Improved FAHP–VIKOR Approach

--Manuscript Draft--

**Manuscript Number:**	PONE-D-25-67607
**Article Type:**	Research Article
**Full Title:**	Risk Identification Method for Automotive Styling Design Tasks Based on an Improved FAHP–VIKOR Approach
**Short Title:**	Risk Identification Method for Automotive Styling Design Tasks
**Corresponding Author:**	Ganshu Sheng<br>Hubei University of Technology<br>wuhan, CHINA
**Keywords:**	automotive styling; design tasks; WBS-RBS; risk identification; FAHP-VIKOR
**Abstract:**	To address the difficulty of quantifying risks in current automotive styling-design task management, this study proposes a risk identification method for automotive styling design tasks based on an improved FAHP–VIKOR approach. First, the work breakdown structure (WBS) is constructed based on the principle of task modularity, and the risk breakdown structure (RBS) is established through the analysis of risk-influencing factors, thereby forming the WBS-RBS coupling matrix for automotive styling design. Then, the improved fuzzy analytic hierarchy process (FAHP) is employed to determine the weights of risk factors such as technical, schedule, and cost risks, while the VlseKriterijumska Optimizacija I Kompromisno Resenje (VIKOR) method is used to perform comprehensive ranking that adapts to the multidimensional and dynamic nature of styling-design risks, thereby achieving quantitative risk assessment and identification. Finally, a case study using the exterior styling concept-design tasks of a sport utility vehicle (SUV) verifies the feasibility of the proposed method.
**Order of Authors:**	Jiefeng Lyu
	Ganshu Sheng
	Zihao Wang
**Additional Information:**	
**Question**	**Response**
**Financial Disclosure**<br><br>Enter a financial disclosure statement that describes the sources of funding for the work included in this submission. Review the submission guidelines for detailed requirements. View published research articles from *PLOS ONE* for specific examples.<br><br>This statement is required for submission and **will appear in the published article** if the submission is accepted. Please make sure it is accurate.	The author(s) received no specific funding for this work.

Powered by Editorial Manager® and ProduXion Manager® from Aries Systems Corporation

**Funded studies**<br>Enter a statement with the following details:<br>• Initials of the authors who received each award<br>• Grant numbers awarded to each author<br>• The full name of each funder<br>• URL of each funder website<br>• Did the sponsors or funders play any role in the study design, data collection and analysis, decision to publish, or preparation of the manuscript?<br><br>Did you receive funding for this work?	
**Competing Interests**<br><br>Use the instructions below to enter a competing interest statement for this submission. On behalf of all authors, disclose any competing interests that could be perceived to bias this work—acknowledging all financial support and any other relevant financial or non-financial competing interests.<br><br>This statement is required for submission and **will appear in the published article** if the submission is accepted. Please make sure it is accurate and that any funding sources listed in your Funding Information later in the submission form are also declared in your Financial Disclosure statement.<br><br>View published research articles from *PLOS ONE* for specific examples.	NO authors have competing interests

**NO authors have competing interests**<br><br>Enter: *The authors have declared that no competing interests exist.*<br><br>**Authors with competing interests**<br><br>Enter competing interest details beginning with this statement:<br><br>*I have read the journal's policy and the authors of this manuscript have the following competing interests: [insert competing interests here]*<br><br>* typeset	
**Ethics Statement**<br><br>Enter an ethics statement for this submission. This statement is required if the study involved:<br><br>• Human participants<br>• Human specimens or tissue<br>• Vertebrate animals or cephalopods<br>• Vertebrate embryos or tissues<br>• Field research<br><br>Write "N/A" if the submission does not require an ethics statement.<br><br>General guidance is provided below. Consult the submission guidelines for detailed instructions. **Make sure that all information entered here is included in the Methods section of the manuscript.**	N/A

Powered by Editorial Manager® and ProduXion Manager® from Aries Systems Corporation

**Format for specific study types**<br><br>**Human Subject Research (involving human participants and/or tissue)**<br>• Give the name of the institutional review board or ethics committee that approved the study<br>• Include the approval number and/or a statement indicating approval of this research<br>• Indicate the form of consent obtained (written/oral) or the reason that consent was not obtained (e.g. the data were analyzed anonymously)<br><br>**Animal Research (involving vertebrate animals, embryos or tissues)**<br>• Provide the name of the Institutional Animal Care and Use Committee (IACUC) or other relevant ethics board that reviewed the study protocol, and indicate whether they approved this research or granted a formal waiver of ethical approval<br>• Include an approval number if one was obtained<br>• If the study involved *non-human primates*, add *additional details* about animal welfare and steps taken to ameliorate suffering<br>• If anesthesia, euthanasia, or any kind of animal sacrifice is part of the study, include briefly which substances and/or methods were applied<br><br>**Field Research**<br><br>Include the following details if this study involves the collection of plant, animal, or other materials from a natural setting:<br>• Field permit number<br>• Name of the institution or relevant body that granted permission	
**Data Availability**<br><br>Authors are required to make all data underlying the findings described fully available, without restriction, and from the time of publication. PLOS allows rare exceptions to address legal and ethical concerns. See the PLOS Data Policy and FAQ for detailed information.	Yes - all data are fully available without restriction

A Data Availability Statement describing where the data can be found is required at submission. Your answers to this question constitute the Data Availability Statement and **will be published in the article**, if accepted.<br><br>**Important:** Stating 'data available on request from the author' is not sufficient. If your data are only available upon request, select 'No' for the first question and explain your exceptional situation in the text box.<br><br>Do the authors confirm that all data underlying the findings described in their manuscript are fully available without restriction?	
**Describe where the data may be found in full sentences. If you are copying our sample text, replace any instances of XXX with the appropriate details.**<br><br>• If the data are **held or will be held in a public repository**, include URLs, accession numbers or DOIs. If this information will only be available after acceptance, indicate this by ticking the box below. For example: *All XXX files are available from the XXX database (accession number(s) XXX, XXX.).*<br>• If the data are all contained **within the manuscript and/or Supporting Information files**, enter the following: *All relevant data are within the manuscript and its Supporting Information files.*<br>• If neither of these applies but you are able to provide **details of access elsewhere**, with or without limitations, please do so. For example:<br><br>*Data cannot be shared publicly because of [XXX]. Data are available from the XXX Institutional Data Access / Ethics Committee (contact via XXX) for researchers who meet the criteria for access to confidential data.*<br><br>*The data underlying the results presented in the study are available from (include the name of the third party*	All relevant data are within the manuscript and its Supporting Information files.

and contact information or URL).<br>• This text is appropriate if the data are owned by a third party and authors do not have permission to share the data.<br><br>* typeset	
Additional data availability information:	

Powered by Editorial Manager® and ProduXion Manager® from Aries Systems Corporation

Manuscript

Click here to access/download;Manuscript;Munuscript - Risk Identification Method.docx

Risk Identification Method for Automotive Styling Design Tasks Based on an Improved FAHP–VIKOR Approach

Jiefeng Lyu [1] · **Ganshu Sheng** [1,2,*] · **Zihao Wang** [1]

1 School of Art and Design, Wuhan University of Technology, Hubei, Wuhan, China
2 School of Industrial Design, Hubei University of Technology, Hubei, Wuhan, China

*Corresponding author: **Ganshu Sheng**
E-mail: shenggs@hbut.edu.cn ORCID: 0009-0001-1581-9030

Received ******* Accepted *******

Abstract

To address the difficulty of quantifying risks in current automotive styling-design task management, this study proposes a risk identification method for automotive styling design tasks based on an improved FAHP–VIKOR approach. First, the work breakdown structure (WBS) is constructed based on the principle of task modularity, and the risk breakdown structure (RBS) is established through the analysis of risk-influencing factors, thereby forming the WBS-RBS coupling matrix for automotive styling design. Then, the improved fuzzy analytic hierarchy process (FAHP) is employed to determine the weights of risk factors such as technical, schedule, and cost risks, while the VlseKriterijumska Optimizacija I Kompromisno Resenje (VIKOR) method is used to perform comprehensive ranking that adapts to the multidimensional and dynamic nature of styling-design risks, thereby achieving quantitative risk assessment and identification. Finally, a case study using the exterior styling concept-design tasks of a sport utility vehicle (SUV) verifies the feasibility of the proposed method.
Keywords: automotive styling, design tasks, WBS–RBS, risk identification, FAHP–VIKOR

1 Introduction

The global automotive industry is experiencing a transformation characterized by the “new four modernizations,” through which automobiles are shifting from mechanical tools to intelligent terminals. As the key interface connecting brand, users, and technology, automotive styling design has gained unprecedented strategic significance. Successful styling design can shape brand recognition, lead aesthetic trends, and directly influence commercial performance [1]. However, automotive styling design projects are complex undertakings that combine artistic creativity with engineering rigor. Their processes span a wide range of critical tasks—market-aesthetic research, styling-concept generation, digital/ clay model development, engineering feasibility verification, cost control, and mass-production process alignment—with long cycles, high investment, and strong interdependencies [2-3]. Numerous risks emerge throughout the design process, such as conflicts between creative styling and engineering feasibility, mismatches between design schemes and user preferences, and failures in new-technology integration. If these risks are not identified or managed in time, they may cause repeated revisions, schedule delays, or even unfavorable market performance after vehicle launch, potentially resulting in economic loss and wasted research and development resources. Therefore, systematically and scientifically identifying potential risks in automotive styling design tasks is crucial for enhancing design-management quality and project success rates.

Traditional risk-management methods—such as the Delphi method [4], brainstorming [5], and fault-tree analysis (FTA) [6]—mainly rely on expert knowledge and qualitative judgment. They lack systematic frameworks and quantitative tools, making them insufficient for addressing the highly complex and interconnected risks in modern styling-design projects.

In recent years, with the increasing digitalization and parallelization of vehicle development processes, researchers have begun to focus on risk identification within automotive product development projects. Yousefi et al. [7] applied a robust data envelopment qnalysis–failure mode and effects analysis (DEA–FMEA) method to prioritize health, safety, and environment (HSE) risks in automotive component manufacturing. Li et al. [8] conducted risk assessment for automotive component projects using the analytic hierarchy process (AHP). Mzougui et al. [9] conducted risk assessment on the automotive supply chain using an improved multi-criteria decision making (MCDM)-based failure mode, effects and criticality analysis (FMECA) approach. Hu et al. [10] used Bayesian networks to assess automotive supply chain risks. Li et al. [11] applied fuzzy analytic hierarchy process (FAHP) to construct a hierarchical outcome model and evaluated risks in automotive styling design projects from a project management perspective. Ju et al. [12] integrated AHP and the entropy weight method (EWM) to quantify evaluation results from both subjective and objective dimensions and thereby select the optimal styling scheme, offering an alternative perspective for risk identification in automotive styling.

In the broader fields of design and engineering, researchers have attempted to systematize risk analysis through methods such as risk matrix analysis, fuzzy comprehensive evaluation (FCE), and AHP. Zhang et al. [13] applied the work breakdown structure–risk breakdown structure (WBS–RBS) method to identify risks among automotive component suppliers. Wu et al. [14] used the WBS–RBS method to identify risks in tunnel and foundation pit construction and applied FCE for risk evaluation. Zhou et al. [15] proposed a risk identification method for metro foundation pits based on fault tree analysis combined with WBS–RBS. Xiang et al. [16] calculated the risk level of bridge design using an AHP–FCE model. Lei et al. [17] integrated AHP with a cloud model to assess risks in highway survey and design. Hyun et al. [18] combined AHP with FTA to identify risks in shield tunnel construction. Pour et al. [19] adopted FAHP to identify risks in natural gas and petroleum drilling processes. Chen et al. [20] constructed an evaluation model using WBS–RBS and AHP to identify risks in cross-sea bridge engineering. Dong et al. [21] analyzed risk factors associated with immersed tunnel construction through WBS–RBS and used an improved genetic algorithm–enhanced AHP to calculate risk weights for evaluation. Chen et al. [22] applied WBS–RBS and an improved FAHP method to identify risks in information system construction projects within coal enterprises. Ji et al. [23] developed evaluation indicators for bridge construction using WBS–RBS and conducted risk identification through Delphi-modified FAHP.

It is evident that current risk identification research for automotive products predominantly focuses on the production and manufacturing stages of automotive components or the procurement and supply chain phases, while studies specifically dedicated to risk identification within vehicle styling design tasks themselves remain scarce. Methods such as WBS–RBS, AHP, and their combinations have been applied in tunneling, bridge construction, and large-scale engineering projects. Yet single-method approaches such as WBS–RBS or AHP cannot fully describe the complex relationships between risks and tasks in styling-design projects: WBS–RBS emphasizes risk identification but not risk evaluation, while AHP suffers from subjectivity in indicator design and judgment-matrix construction, together with challenges in consistency testing.

To effectively and objectively quantify styling-design task risks, this study draws on risk-influencing factors in automotive styling design and introduces the concept of WBS–RBS. Combining FAHP and the vlsekriterijumska optimizacija I kompromisno resenje (VIKOR) MCDM method, we propose a risk-identification method tailored to automotive styling design that provides clear risk-control focus and decision-making support in styling-design management.

2 Overview

The FAHP–VIKOR-based risk identification method for automotive styling design tasks consists of four major steps: construction of work breakdown structure (WBS) for styling-design tasks, construction of risk breakdown structure (RBS) for styling-design tasks, construction of the WBS–RBS matrix, and quantification of design task risks based on FAHP-VIKOR, as illustrated in Figure 1.

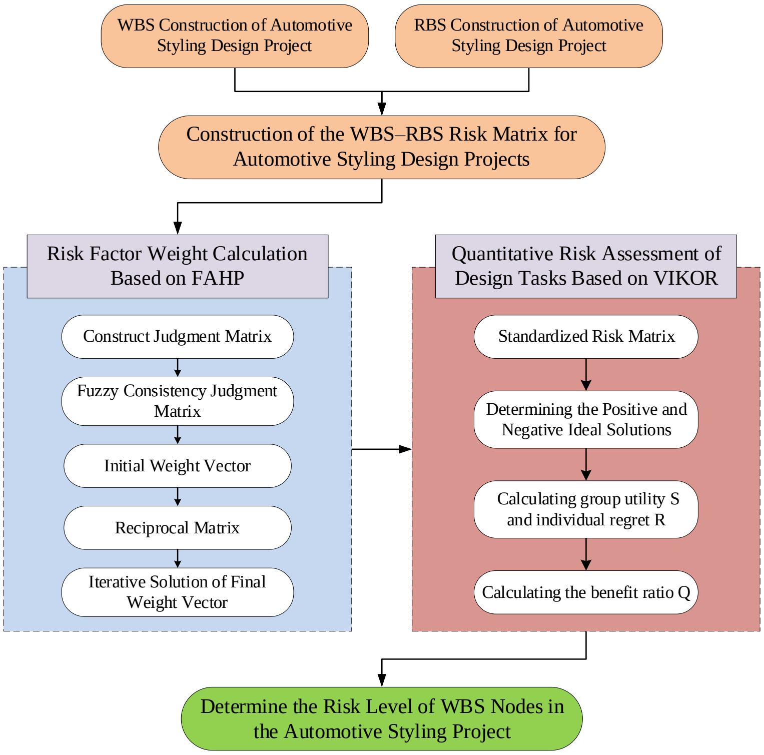

Fig. 1 Risk Identification Process for Automotive Styling Design Tasks Based on FAHP-VIKOR

(1) Construction of WBS for styling-design tasks: Based on automotive structural characteristics and principles of physical decomposition between units, a WBS is developed for each stage of styling design.

(2) Construction of RBS for styling-design tasks: Based on international electrotechnical commission (IEC) risk-assessment standards, China automotive technology and research center (CATARC) styling-process guidelines, and enterprise-level styling-process management systems, risk-influencing factors are identified and organized into a general risk-indicator system.

(3) Construction of the WBS–RBS matrix: Work packages at the lowest WBS level and risk factors at the lowest RBS level serve as matrix rows and columns, respectively. The product of risk occurrence probability and loss severity is defined as the risk value of each matrix element.

(4) Quantification of design-task risks: Improved FAHP–VIKOR is applied to calculate risk-factor weights for each WBS task and to evaluate comprehensive benefit ratios for identifying risk levels across styling-design tasks.

The structure of this paper is as follows: Section 3 constructs the WBS–RBS matrix for automotive styling design. Section 4 introduces the improved FAHP–VIKOR method for risk quantification. Section 5 presents a case study using a sport utility vehicle (SUV) exterior styling-design project. Section 6 concludes the work and discusses future directions. This paper's contributions include: (a) constructing a WBS–RBS matrix for automotive styling-design stages;(b) quantifying styling-design task risks using an improved FAHP–VIKOR algorithm.

3 Construction of the WBS–RBS Matrix for Automotive Styling Design

3.1 WBS of Automotive Styling Design Tasks

The WBS is a project-management tool that decomposes an automotive styling-design project into manageable tasks. First, "automotive styling-design development" is defined as the first-level overall objective. Based on standard styling-design workflows, this objective is decomposed into five second-level strategic stages: Project initiation and preparation, creative design and exploration, design refinement and digital-model development, hard-model verification and evaluation, final delivery and archiving. These stages cover the entire process from early conceptual exploration to final production support.

Within the creative design, digital-surface development, and hard-model evaluation stages, the WBS is constructed according to the physical structure of the automobile and the principle of physical modular decomposition among system units [24]. Automotive styling consists mainly of exterior and interior sections, each divided into multiple modules, and each module consists of several components. Together, they constitute the full WBS of styling design. For example, the exterior consists of three major modules-body, front fascia, and rear fascia. The body module includes the A-pillar surface, B-pillar surface, C-pillar surface, D-pillar surface, roof surface, windows, doors, front/rear fenders, wheels, etc. The corresponding WBS structure is shown in Figure 2.

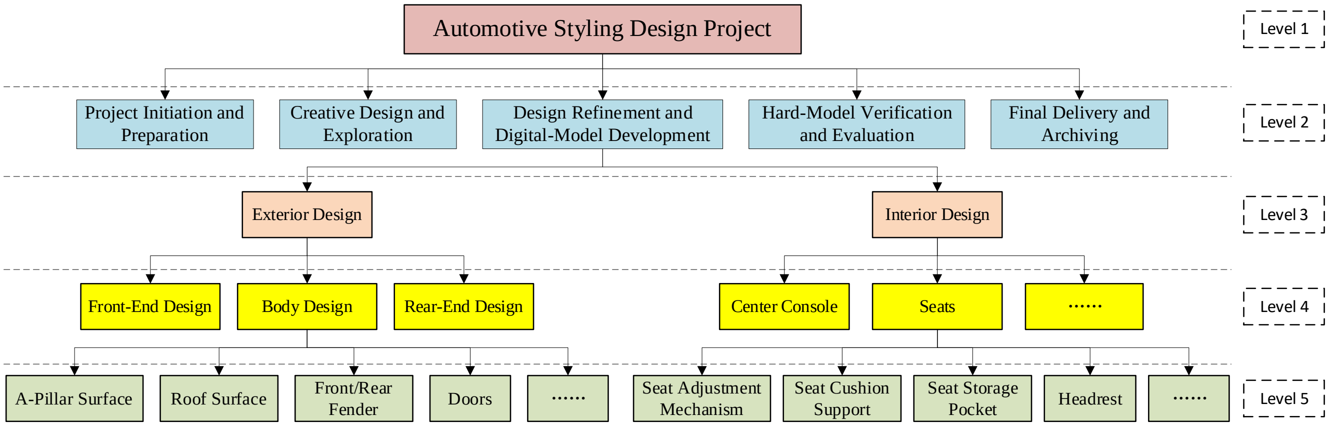

Fig. 2 WBS Decomposition Diagram for Automotive Styling Design

3.2 RBS of Automotive Styling-Design Tasks

Risks in automotive styling design primarily arise from the potential adverse impacts of styling (appearance, structural layout, materials, proportions, detail resolution, etc.) on safety, functionality, human–machine interaction, manufacturability, and regulatory compliance [25]. Based on the risk-management framework outlined in IEC 31010:2009, supplemented by international and domestic standards (GB/T 34590 series, ISO 21448, ISO 15005, GB/T 43407), CATARC styling-process guidelines, and enterprise-level styling-review processes (e.g., Dongfeng, SAIC, Geely), risk factors throughout the styling-design lifecycle are grouped into five major dimensions.

(1) Technical Risks

During the transition from sketches to digital models, distortions in surface form frequently occur, and unforeseen conflicts may arise with engineering structures, material/manufacturing constraints, or regulatory requirements. These issues often lead to repeated revisions or complete redesigns. Examples include: Feasibility of stamping key body surfaces (e.g., hood, tailgate),

Aerodynamic issues for aggressive styling geometries (e.g., low-profile body, oversized spoiler).

(2) Schedule Risks

Due to high concurrency and frequent iteration in styling-development processes, key tasks are easily affected by resource conflicts or slow decision-making, compressing available design windows. For instance: computer-aided styling (CAS) model release delays caused by multiple styling revisions, A-surface disputes leading to postponed data release for engineering teams.

(3) Cost Risks

Cost risk encompasses not only the explicit expenditures arising from design adjustments—such as mold modification or prototype re-manufacturing—but also the implicit premiums associated with procuring special materials and processes. Examples include whether the costs of uniquely shaped components (e.g., customized grilles or fastback tailgates) exceed the budget by 10%, and whether the adoption of special paint finishes or new eco-friendly color, material, finishing (CMF) materials surpasses the allocated budget by 10%.

(4) Resource and Collaboration Risks

Key personnel turnover may disrupt styling language continuity; cross-team collaboration may be hindered by unclear responsibilities, low communication efficiency, or system faults, obstructing task flow. Examples: Departure of lead exterior designer disrupts consistency in front-fascia design, Conflicts between styling goals (e.g., aggressive A-pillar angle) and engineering demands for driver visibility.

(5) External and Compliance Risks

These arise from uncertainties in the macro environment. Regulatory changes may force redesigns; supply-chain fluctuations may affect key component availability; public opinion may create pressure for design modifications. Examples: Hood redesign to comply with updated pedestrian-protection regulations, Large-screen interior layout conflicting with market-specific crash standards.

RBS decomposes risks into hierarchical categories and subcategories, enabling systematic identification and analysis. Based on the above analysis, a multi-level risk indicator system for automotive styling design is developed, as illustrated in Figure 3.

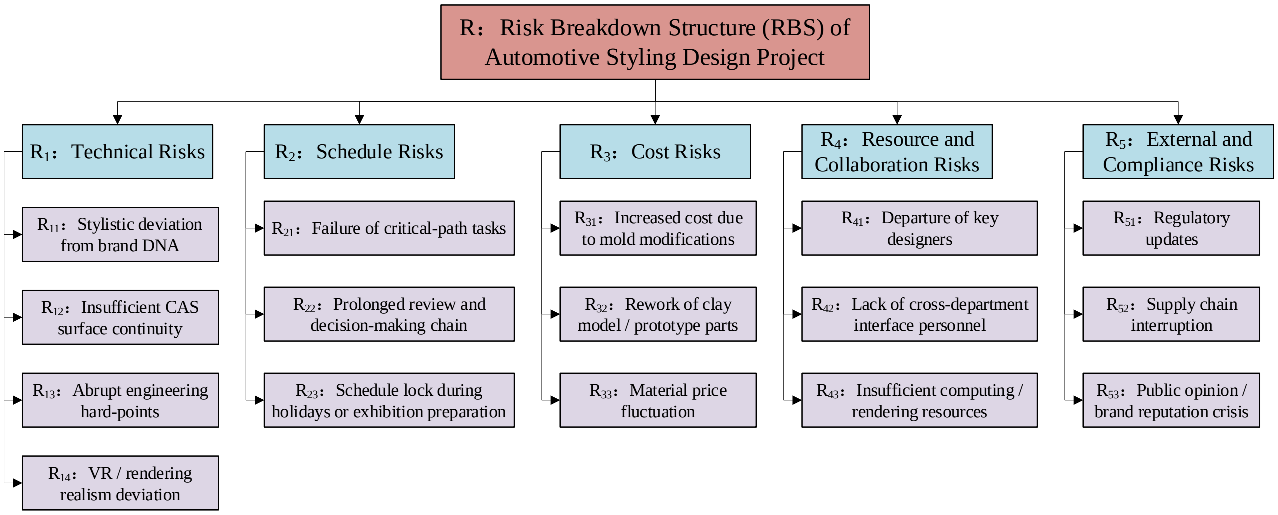

Fig. 3 RBS Decomposition Diagram for Automotive Styling Design Project

3.3 Construction of the WBS–RBS Matrix

The WBS–RBS method integrates WBS and RBS to identify risks by cross-analyzing project tasks and risk factors. The construction process includes the following steps:

(1) Constructing the WBS–RBS matrix

All work packages in the WBS are arranged as the rows of the matrix, while all risk factors in the RBS serve as the columns. The intersections between rows and columns represent potential risk factors associated with each specific design task. The risk value of the j-th risk factor in the i-th styling design task is denoted as x_{ij}.

(2) Determination of the matrix cell risk value x_{ij}

Within automotive styling design projects, the probability that risks occur during design, production, or delivery processes, as well as the magnitude of the loss incurred, cannot be accurately quantified. Consequently, the abstract notion of risk must be transformed into specific, measurable numerical values. To this end, a cardinal fuzzy evaluation approach is adopted, whereby integers ranging from 0 to 9 are used to represent the severity level, with 0 indicating the lowest and 9 indicating the highest magnitude. As discussed in [26], the magnitude of risk is jointly influenced by the probability of occurrence and the extent of the associated loss. Accordingly, the risk value x_{ij} is calculated as follows:

$$x_{ij} = P_{ij} \times L_{ij} \tag{1}$$

where x_{ij} denotes the degree of risk associated with risk factor j at WBS task node i; P_{ij} represents the probability of occurrence of risk factor j at task node i; and L_{ij} denotes the loss severity associated with risk factor j at task node i.

For each task node, the values of P and L are assigned by an expert panel in automotive styling design, based on collective discussion and professional judgement. The specific rating criteria for P and L are shown in Table 1.

Table 1 Rating criteria for risk occurrence probability and loss severity

Risk Occurrence Probability	P-value	Risk Loss Severity	L-value
Completely foreseeable	9	Severe loss	9
Highly likely	7	Major loss	7
Likely and frequent	5	Moderate loss	5
Possible but infrequent	3	Minor loss	3
Rarely possible	1	Very slight loss	1
Completely impossible	0	No loss	0
Intermediate values between two adjacent judgments	2, 4, 6, 8	Intermediate values between two adjacent judgments	2, 4, 6, 8

In combination with the WBS decomposition and risk factor analysis described in the preceding sections, a WBS–RBS matrix for the automotive styling design process is constructed, as shown in Fig. 4. The cell values x_{ij} in the matrix are determined based on expert scoring by an automotive styling design specialist panel from a major automobile manufacturer.

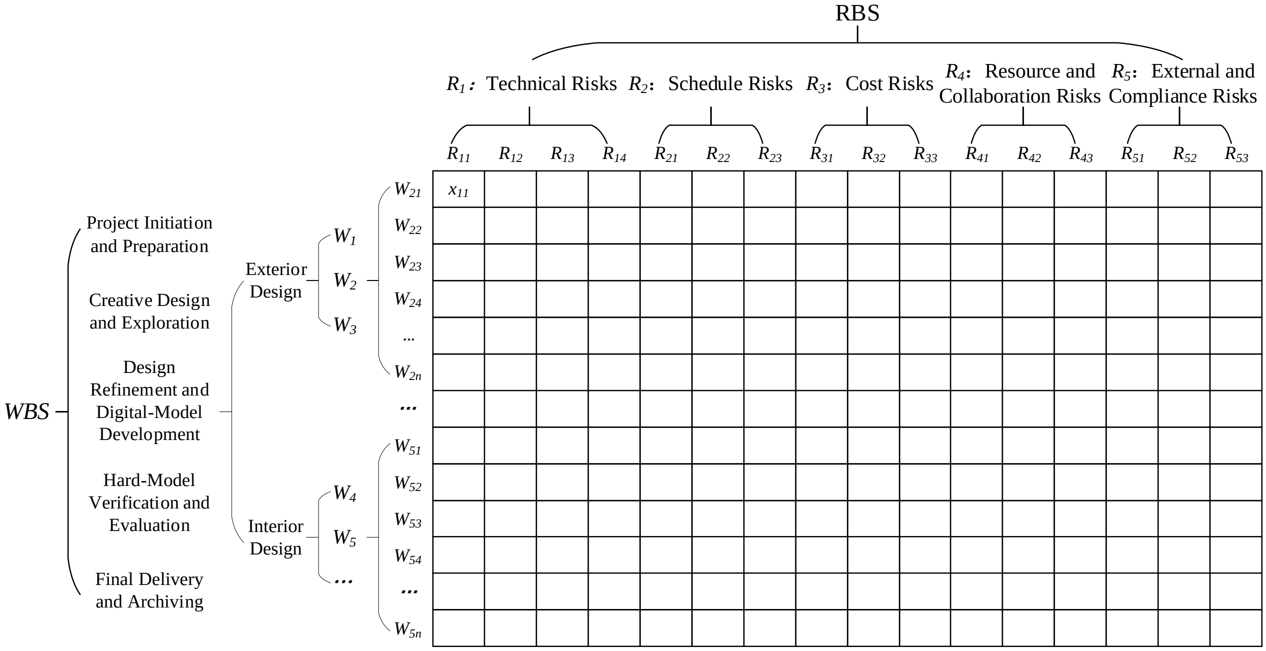

Fig. 4 WBS-RBS Risk Matrix for Automotive Styling Design

4 Risk Identification for Automotive Styling-Design Tasks

4.1 Determining Risk-Factor Weights with FAHP

The automotive styling design process involves numerous and highly complex risk factors. If the AHP were directly applied to identify risk nodes, two challenges would arise: first, it would be difficult to reflect the inherent fuzziness embedded within human judgment when assessing relationships among factors; second, when the pairwise comparison matrix does not satisfy the consistency requirement, repeated procedures of adjustment, validation, re-adjustment, and re-validation would be necessary. For these reasons, an improved FAHP [27] is employed to determine risk weights. By transforming the reciprocal matrix into a fuzzy consistency judgment matrix, this method not only accommodates the fuzziness of human subjective cognition, but also resolves the consistency issue of the judgment matrix. The construction procedure is as follows.

Step 1: Based on the WBS–RBS matrix described in Section 3.3, construct a pairwise comparison matrix A for all factors.

$$a_{ij} = \begin{cases} 0.5 & s(i) = s(j) \\ 1.0 & s(i) > s(j) \\ 0 & s(i) < s(j) \end{cases} \tag{2}$$

$$A = (a_{ij})_{n\times n} = \begin{bmatrix} a_{11} & \cdots & a_{1n} \\ \vdots & \ddots & \vdots \\ a_{m1} & \cdots & a_{mn} \end{bmatrix} \tag{3}$$

Here, $s(i)$ and $s(j)$ denote the relative importance of risk factor i and j, respectively, and the relative importance is expressed by the degree of risk at a given task node associated with the corresponding risk factor. The comparison result of risk factor iii relative to factor j is denoted by a_{ij}.

Step 2: Using the pairwise comparison matrix obtained above, compute the fuzzy consistency judgment matrix. First, sum the elements of matrix A row by row to obtain $p_i = \sum_{j=1}^{n} a_{ij}$, Then the element of the fuzzy consistency judgment matrix is $p_{ij} = [(p_i - p_j) / 2n] + 0.5$, and the fuzzy consistency judgment matrix can be represented as $P = [p_{ij}]_{n\times n}$.

Step 3: Apply the row-sum normalization method to derive the initial weight vector $w^{(0)} = (w_1, w_2, \ldots, w_n)^T$,using the following expression:

$$w^i = \sum_{j=1}^{n} p_{ij} / \sum_{i=1}^{n}\sum_{j=1}^{n} p_{ij} \tag{4}$$

Step 4: Transform the fuzzy consistency judgment matrix $P = [p_{ij}]_{n\times n}$ into reciprocal matrix $E = [e_{ij}]_{n\times n}$ by $e_{ij} = p_{ij} / p_{ji}$.

Step 5: Take the initial weight vector $w^{(0)}$ as the initial vector V^0 for the power iteration method, and further compute a more accurate weight vector V^k. Given the initial vector $V^0 = (V_{01}, V_{02}, \ldots, V_{0n})^T$, use the iterative formula $V^{k+1}=EV^k$ to obtain V^{k+1}. Calculate the infinity norm $\|V_{k+1}\|_\infty$. If $|\|V_{k+1}\|_\infty - \|V_k\|_\infty| < \varepsilon$, then $\|V_{k+1}\|_\infty$ is regarded as the maximum eigenvalue λ_{max}. Normalize V^{k+1} as follows:

$$V^{k+1} = \left[\frac{V_{k+1,1}}{\sum_{i=1}^{n} V_{k+1,i}}, \frac{V_{k+1,2}}{\sum_{i=1}^{n} V_{k+1,i}}, \ldots, \frac{V_{k+1,n}}{\sum_{i=1}^{n} V_{k+1,i}}\right]^{\mathrm{T}} \tag{5}$$

The resultant vector $w^{(k)} = V^{(k+1)}$ is the final weight vector, i.e., the iteration stops. If the convergence condition is not satisfied, compute and use it as a new initial vector for the next iteration.

$$V^k = V_{k+1} / \|V_{k+1}\|_\infty = [V_{k+1,1} / \|V_{k+1}\|_\infty, V_{k+1,2} / \|V_{k+1}\|_\infty, \ldots, V_{k+1,n} / \|V_{k+1}\|_\infty]^T \tag{6}$$

4.2 Quantifying Design-Task Risks Using the VIKOR Method

Automotive styling design tasks involve multiple mutually conflicting criteria, including creativity alignment, cost control, and schedule progress. The VIKOR method, based on multiple decision attributes, provides a quantitative ranking that accommodates both group utility and individual regret, and thus better matches the dynamic risk characteristics of styling design tasks while avoiding misjudgment caused by single-dimensional evaluation [28]. The specific procedure for calculating the WBS node risk level using the VIKOR method is as follows:

Step 1: Standardizing the WBS–RBS matrix

Each cell value in the WBS–RBS matrix is normalized using range normalization for each indicator, as expressed below:

$$z_{ij} = \frac{x_{ij} - min_j}{max_j - min_j} \tag{7}$$

where Zij denotes the normalized value of x_{ij} represents the value of the j-th risk factor for the i-th WBS node; and max_j、min_j represent the maximum and minimum values of column j, respectively.

Step 2: Determining the positive and negative ideal solutions

For each column j of the normalized WBS–RBS matrix, determine the positive and negative ideal solutions, i.e., the maximum and minimum values in that column ($max\, z_j$、$min\, z_j$).

Step 3: Calculating group utility S and individual regret R

S represents the sum of the relative distances from the optimal object, denoting the comprehensive deviation degree of an evaluated object relative to the positive ideal solution—smaller values indicate better performance. R represents the maximum distance ratio of any single indicator relative to the optimal object—again, smaller values indicate superior performance.

$$S_i = \sum_{j=1}^{n}\left(w_j \frac{max\, z_j - z_{ij}}{max\, z_j - min\, z_j}\right) \qquad R_i = \max_{1\le j\le n} \; w_j \frac{max\, z_j - z_{ij}}{max\, z_j - min\, z_j} \tag{8}$$

Where w_j denotes the weight of the j-th risk factor.

Step 4: Calculating the benefit ratio Q

Based on the obtained S_i and R_i, compute the benefit ratio Q_i using:

$$Q_i = v\left[\frac{(S_i - min\,S_i)}{(max\,S_i - min\,S_i)}\right] + (1-v)\left[\frac{(R_i - min\,R_i)}{(max\,R_i - min\,R_i)}\right] \tag{9}$$

where Q_i denotes the benefit ratio; v denotes the decision-making coefficient with a value range of [0,1]. When v>0.5, the evaluation result is more inclined to group utility and therefore tends toward risk preference; when v=0.5, equal consideration is given to group utility and individual regret, representing a risk-balanced pattern; when v<0.5, emphasis is placed on individual regret, thereby reflecting a risk-averse pattern. $max\,S_i$ and $min\,S_i$ denote the maximum and minimum values of S, while $max\,R_i$ and$min\,R_i$ denote the maximum and minimum values of R.

Step 5: Determining the ranking of WBS nodes

Sort the calculated Q values in ascending order, where a higher Q indicates higher compatibility with design objectives and therefore lower risk, and vice versa. The detailed criteria are shown in Table 2.

Table 2 Risk level classification criteria for automotive styling design

Benefit ratio Q	Risk identification result	Recommended handling
0–0.3	High risk	Significant risk likely to cause major rework, substantial cost increase, or compliance issues; immediate design adjustment required
0.3–0.7	Medium risk	Moderate risk likely to cause partial rework; additional resources or targeted mitigation plan required
0.7–1	Low risk	Minor risk; minimal conflict among styling tasks; no additional mitigation measures required

5 Case Study: Exterior Styling Concept-Design Tasks of an SUV

To validate the proposed FAHP–VIKOR risk identification method, a case study is conducted using the exterior styling concept-design phase of a five-seat sedan-type SUV. The exterior styling tasks include front-fascia refinement, body-line development, and rear-fascia sculpting. Key components include the grille, headlamps, side profiles, taillamps, and other styling elements, forming a complete task system (see Figure 5).

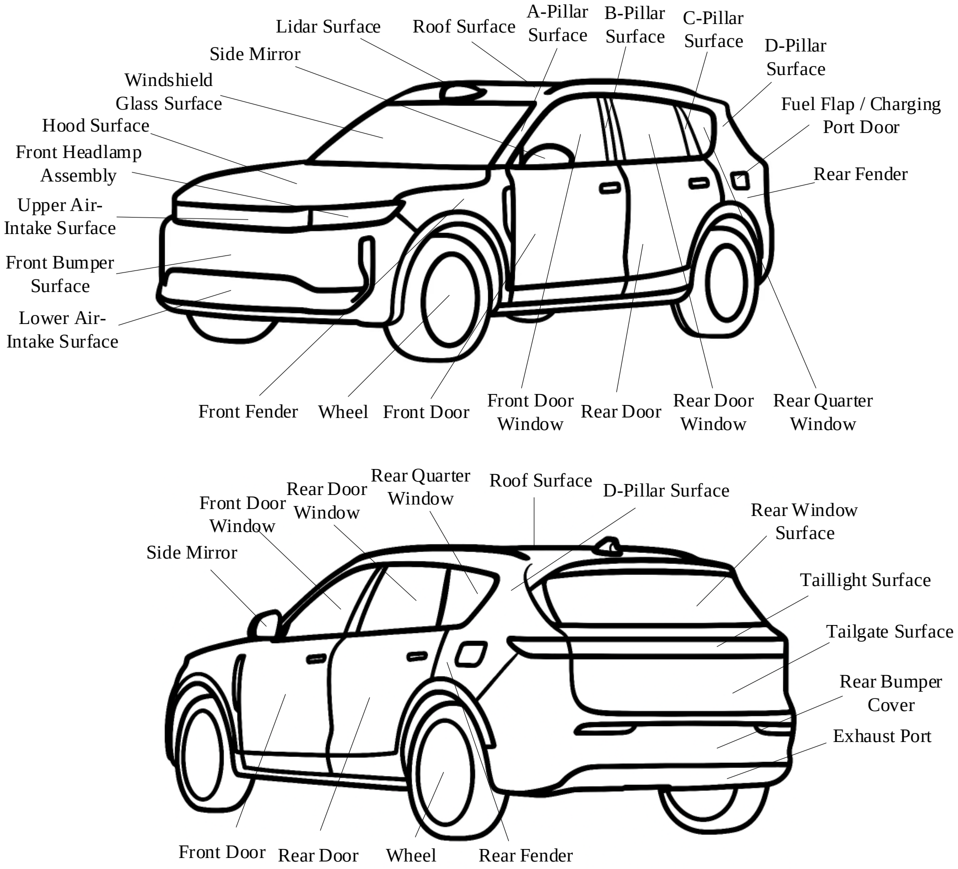

Fig. 5 Creative Exterior Styling Design Task Classification for a Certain SUV

5.1 Construction of the WBS–RBS Matrix for Exterior Styling

(1) Establishing the WBS of automotive exterior styling design

Based on the WBS construction process described in Section 3.1, the creative exterior styling design of an SUV is decomposed into three major categories, namely front-end design, rear-end design, and body design. Each category consists of multiple exterior components, resulting in a total of 27 WBS work packages, as shown in Table 3.

Table 3 WBS decomposition of creative exterior styling design for a certain SUV

	Primary Structure	WBS	Secondary Structure	WBS
Exterior Component Design for Automotive Styling Design Project	Front-End Design	W_1	Front Headlamp Assembly	W_{11}
			Upper Air-Intake Surface	W_{12}
			Front Bumper Surface	W_{13}
			Lower Air-Intake Surface	W_{14}
			Hood Surface	W_{15}
			Windshield Glass Surface	W_{16}
	Body Design	W_2	A-Pillar Surface	W_{21}
			B-Pillar Surface	W_{22}
			C-Pillar Surface	W_{23}
			D-Pillar Surface	W_{24}
			Roof Surface	W_{25}
			Lidar Surface	W_{26}
			Front Door Window	W_{27}
			Rear Door Window	W_{28}
			Rear Quarter Window	W_{29}
			Front Door	W_{210}
			Rear Door	W_{211}
			Front Fender	W_{212}
			Rear Fender	W_{213}
			Fuel Flap / Charging Port Door	W_{214}
			Wheel	W_{215}
			Side Mirror	W_{216}
	Rear-End Design	W_3	Rear Window Surface	W_{31}
			Taillight Surface	W_{32}
			Tailgate Surface	W_{33}
			Rear Bumper Cover	W_{34}
			Exhaust Port	W_{35}

(2) Building the WBS–RBS Matrix

The above 27 lowest-level WBS work packages are taken as the rows of the matrix, and the 16 lowest-level RBS risk factors described in Section 2.2 are taken as the columns to form the WBS–RBS coupling matrix. The expert panel on automotive styling design then assigns P and L scores for each of the 16 risk factors at each of the 27 WBS task nodes, followed by calculating the risk value x_{ij} for each matrix cell according to Equation (1). The final results are shown in Table 4.

Table 4 WBS–RBS risk matrix for creative exterior styling design of an SUV

**WBS Decomposition**		**RBS Decomposition**															
		Technological R_1				*Schedule Risk R_2*			*Cost Risk R_3*			*Resource and Collaboration Risk R_4*			*External and Compliance Risk R5*		
		R_{11}	R_{12}	R_{13}	R_{14}	R_{21}	R_{22}	R_{23}	R_{31}	R_{32}	R_{33}	R_{41}	R_{42}	R_{43}	R_{51}	R_{52}	R_{53}
Front-End Design (W_1)	W_{11}	42	35	28	0	30	25	12	0	0	15	8	20	0	16	6	9
	W_{12}	56	42	35	0	35	30	15	0	0	20	12	25	0	20	8	12
	W_{13}	63	49	42	0	42	35	18	0	0	25	15	30	0	24	9	15
	W_{14}	49	35	30	0	30	25	12	0	0	18	9	24	0	18	6	9
	W_{15}	35	28	20	0	25	20	9	0	0	12	6	16	0	12	4	6
	W_{16}	20	15	12	0	15	12	6	0	0	6	3	9	0	8	2	3
Rear-End Design (W_2)	W_{21}	30	25	20	0	25	20	9	0	0	15	6	18	0	15	6	9
	W_{22}	20	16	12	0	16	12	6	0	0	9	4	12	0	9	3	4
	W_{23}	25	20	15	0	20	16	8	0	0	12	6	15	0	12	4	6
	W_{24}	18	12	9	0	12	9	4	0	0	6	3	9	0	6	2	3

	W_{25}	15	9	6	0	9	6	3	0	0	4	2	6	0	4	1	2
	W_{26}	49	42	35	0	42	35	18	0	0	30	15	28	0	25	12	18
	W_{27}	12	9	6	0	9	6	3	0	0	4	2	6	0	4	1	2
	W_{28}	15	12	9	0	12	9	4	0	0	6	3	8	0	6	2	3
	W_{29}	12	8	6	0	8	6	3	0	0	4	2	6	0	4	1	2
	W_{210}	25	20	15	0	20	16	8	0	0	12	6	15	0	12	4	6
	W_{211}	24	18	12	0	18	12	6	0	0	9	4	12	0	9	3	4
	W_{212}	28	24	16	0	24	18	9	0	0	15	8	18	0	15	6	9
	W_{213}	20	16	12	0	16	12	6	0	0	9	4	12	0	9	3	4
	W_{214}	9	6	4	0	6	4	2	0	0	3	1	4	0	3	1	1
	W_{215}	30	25	20	0	25	20	9	0	0	18	9	20	0	18	8	12
	W_{216}	18	12	9	0	12	9	4	0	0	8	4	9	0	8	3	4
Body Design (W_3)	W_{31}	12	9	6	0	9	6	3	0	0	4	2	6	0	4	1	2
	W_{32}	35	28	20	0	28	24	12	0	0	20	12	24	0	20	9	15
	W_{33}	25	20	15	0	20	16	8	0	0	12	6	15	0	12	4	6
	W_{34}	56	49	42	0	49	42	20	0	0	35	20	35	0	30	15	24
	W_{35}	15	12	9	0	12	9	4	0	0	6	3	8	0	6	2	3

5.2 Calculation of Risk-Factor Weights

To illustrate the calculation process, the front headlamp assembly (W_{11}) is used as an example. Because certain risk factors (e.g., R_{14}, R_{31}, R_{32}, R_{43}) do not apply during early styling-concept development, they are assigned zero risk. Corresponding judgment matrices $W\text{–}R$, $W\text{–}R_1$, $W\text{–}R_2$, $W\text{–}R_4$, and $W\text{–}R_5$ are constructed (Table 5).

Table 5 Judgement Matrix

judgment matrix of *W-R*	judgment matrix of *W-R₁*	judgment matrix of *W-R₂*	judgment matrix of *W-R₄*	judgment matrix of *W-R₅*
$\begin{bmatrix} 0.5 & 1.0 & 1.0 & 1.0 & 1.0 \\ 0.0 & 0.5 & 1.0 & 1.0 & 1.0 \\ 0.0 & 0.0 & 0.5 & 0.0 & 0.0 \\ 0.0 & 0.0 & 1.0 & 0.5 & 0.0 \\ 0.0 & 0.0 & 1.0 & 1.0 & 0.5 \end{bmatrix}$	$\begin{bmatrix} 0.5 & 1.0 & 1.0 \\ 0.0 & 0.5 & 1.0 \\ 0.0 & 0.0 & 0.5 \end{bmatrix}$	$\begin{bmatrix} 0.5 & 1.0 & 1.0 \\ 0.0 & 0.5 & 1.0 \\ 0.0 & 0.0 & 0.5 \end{bmatrix}$	$\begin{bmatrix} 0.5 & 0.0 \\ 1.0 & 0.5 \end{bmatrix}$	$\begin{bmatrix} 0.5 & 1.0 & 1.0 \\ 0.0 & 0.5 & 0.0 \\ 0.0 & 1.0 & 0.5 \end{bmatrix}$

Using the W-R judgment matrix as an example, the corresponding fuzzy consistency judgment matrix is obtained as:

$$P = \begin{bmatrix} 0.5 & 0.6 & 0.9 & 0.8 & 0.7 \\ 0.4 & 0.5 & 0.8 & 0.7 & 0.6 \\ 0.1 & 0.2 & 0.5 & 0.4 & 0.3 \\ 0.2 & 0.3 & 0.6 & 0.5 & 0.4 \\ 0.3 & 0.4 & 0.7 & 0.6 & 0.5 \end{bmatrix}$$

The initial weight vector obtained by the row-sum normalization method is:

$$w^{(0)} = [0.28,\ 0.24,\ 0.12,\ 0.16,\ 0.20]^T$$

The reciprocal judgment matrix is:

$$E = \begin{bmatrix} 1.0000 & 1.5000 & 9.0000 & 4.0000 & 2.3333 \\ 0.6667 & 1.0000 & 4.0000 & 2.3333 & 1.5000 \\ 0.1111 & 0.2500 & 1.0000 & 0.6667 & 0.4286 \\ 0.2500 & 0.4286 & 1.5000 & 1.0000 & 0.6667 \\ 0.4286 & 0.6667 & 2.3333 & 1.5000 & 1.0000 \end{bmatrix}$$

With a convergence precision of 0.0001 and four iterations, the final weights obtained by iterative computation are:

$$w = [0.4253,\ 0.2505,\ 0.0613,\ 0.1025,\ 0.1604]^T$$

Following this procedure, both the criterion-layer and indicator-layer weights are obtained, as shown in Table 6. Ranking the indicators further determines which risk factors dominate at this node.

Table 6 Risk factor weights for creative exterior styling design of an SUV

Risk Factors (Weight)	Risk Factor	Weight	Risk Value	Aggregate Weight	Rank
Technological Risk R_1（0.4253）	R_{11}	0.5954	42	0.2532	1
	R_{12}	0.2763	35	0.1175	3
	R_{13}	0.1283	28	0.0546	8
Schedule Risk R_2（0.2505）	R_{21}	0.5954	30	0.1491	2
	R_{22}	0.2763	25	0.0692	6
	R_{23}	0.1283	12	0.0321	10
Cost Risk R_3（0.0613）	R_{33}	1	15	0.0613	7
Resource & Collaboration Risk R4（0.1025）	R_{41}	0.25	8	0.0256	11
	R_{42}	0.75	20	0.0769	5
External & Compliance Risk R_5（0.1604）	R_{41}	0.5954	16	0.0955	4
	R_{42}	0.1283	6	0.0206	12
	R_{43}	0.2763	9	0.0443	9

5.3 Quantitative Risk Assessment and Analysis

According to Equation (7), the WBS–RBS matrix is standardized, from which the maximum and minimum values of each column (max_j, min_j) are obtained. Combined with the FAHP-derived risk weights at W_{35}, the group utility S_{35} and individual regret R35 are calculated using Equation (8).

The same procedure is applied to the remaining 26 WBS nodes. Based on Equation (9), the benefit ratio Q of each node is computed and subsequently ranked. According to Table 2, each WBS node is then classified into corresponding risk levels. The results are summarized in Table 7.

Table 7 Summary of VIKOR results (risk-balanced type)

WBS Node	S	R	Q	Rank	Risk Level
W_{11}	0.4513	0.0985	0.3781	22	Medium Risk
W_{12}	0.2745	0.0486	0.1778	25	High Risk
W_{13}	0.1263	0.0243	0.0483	26	High Risk
W_{14}	0.3880	0.0663	0.2753	23	High Risk
W_{15}	0.5855	0.1315	0.5195	20	Medium Risk
W_{16}	0.8156	0.2016	0.7912	10	Low Risk
W_{21}	0.5894	0.1547	0.5721	18	Medium Risk
W_{22}	0.7865	0.2020	0.7770	11	Low Risk
W_{23}	0.6926	0.1782	0.6765	13	Medium Risk
W_{24}	0.8612	0.2110	0.8352	8	Low Risk
W_{25}	0.9297	0.2251	0.9013	5	Low Risk
W_{26}	0.2028	0.0657	0.1780	24	High Risk
W_{27}	0.9437	0.2392	0.9392	3	Low Risk
W_{28}	0.8798	0.2255	0.8764	6	Low Risk
W_{29}	0.9500	0.2392	0.9424	2	Low Risk
W_{210}	0.6926	0.1782	0.6765	15	Medium Risk
W_{211}	0.7553	0.1832	0.7199	14	Low Risk
W_{212}	0.6117	0.1641	0.6040	17	Medium Risk
W_{213}	0.7865	0.2020	0.7770	11	Low Risk
W_{214}	1.0000	0.2537	1.0000	1	Low Risk
W_{215}	0.5553	0.1547	0.5545	19	Medium Risk
W_{216}	0.8483	0.2110	0.8286	9	Low Risk
W_{31}	0.9437	0.2392	0.9392	3	Low Risk
W_{32}	0.4685	0.1313	0.4585	21	Medium Risk
W_{33}	0.6926	0.1782	0.6765	15	Medium Risk
W_{34}	0.0328	0.0328	0.0186	27	High Risk
W_{35}	0.8798	0.2255	0.8764	6	Low Risk

After identifying the high-risk WBS nodes of the automotive styling design project, different colors are assigned to different risk levels in Oracle P6 EPPM, enabling intuitive identification of high-risk styling design tasks. The resulting interactive Gantt chart is shown in Fig. 6.

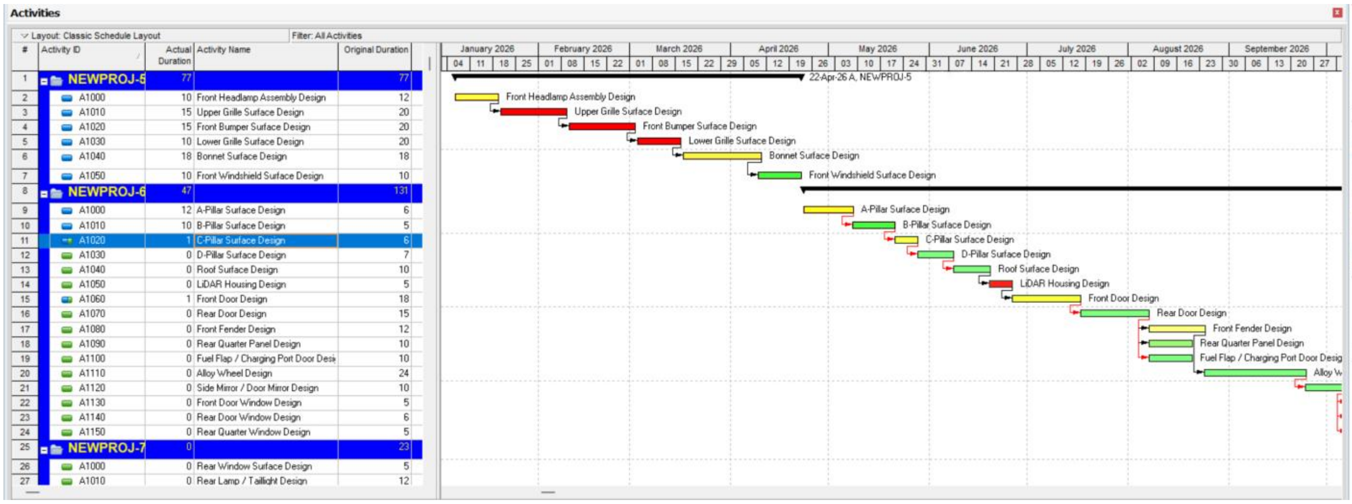

Fig. 6 Gantt Chart for WBS Tasks of Automotive Styling Design Project

From Table 7 and Fig. 6, there are five high-risk nodes (18.5%), eight medium-risk nodes (29.6%), and fourteen low-risk nodes (51.9%). The high-risk nodes include W_{12} (upper air-intake surface), W_{13} (front bumper surface), W_{14} (lower air-intake surface), W_{26} (lidar surface), and W_{34} (rear bumper surface). These nodes show a high level of risk across most of the five major dimensions.

Specifically, front-end surfaces (W_{12}, W_{13}, W_{14}) exhibit high technological risk (R_1) and schedule risk (R_2). The pursuit of distinctive brand identity may lead to aggressive styling language causing (R_{11}: stylistic deviation from brand DNA). In addition, design feasibility conflicts may arise (R_{13}: abrupt engineering hard-points) or aerodynamic performance issues (R_{12}: insufficient CAS surface continuity), leading to repeated cross-departmental review loops (R_{22}), resulting in schedule delays and cost increases.

As an emerging smart-mobility component, W_{26} exhibits pronounced epochal characteristics and is exposed to both technological risk (R_1) and external compliance risk (R_{51}: regulatory updates). Global autonomous driving regulations may trigger design rework. W_{34} shares similar risk patterns with the front-end components, as collision-safety requirements and proportion adjustments frequently cause design re-iteration.

Medium-risk nodes include W_{11}, W_{15}, W_{21}, W_{23}, W_{210}, W_{212}, W_{215}, W_{32} and W_{33}, whose risks tend to be more localized. For instance, W_{11} and W_{32} face technological difficulty (R_{12}) and material cost (R_{33}) due to complex optical appearance. W_{15}, W_{21} and W_{23} bear ergonomic- and regulatory-related risks (R_{13}, R_{51}), possibly leading to partial rework. W_{210}, W_{212} and W_{33} mainly exhibit coordination-related risks (R_{42}), causing increased communication costs. W_{215} needs to balance lightweighting and structural integrity, presenting noticeable technological and cost risk.

6 Conclusion

Taking into account enterprise-level styling design procedures and relevant standards, five major risk factors for automotive styling design tasks are identified. On this basis, an FAHP–VIKOR based method for risk identification of automotive styling design tasks is proposed. By constructing a WBS–RBS risk matrix for automotive styling design, the method employs an improved FAHP–VIKOR procedure to perform fuzzy weighting of risk factors and compromise ranking under multiple criteria. The method is able to effectively determine the weights of evaluation indicators and achieve an integrated ranking of styling design task risks.

Furthermore, an engineering case study of exterior styling design for an SUV model developed by a domestic automobile manufacturer demonstrates that, among the 27 exterior styling sub-tasks examined, the proposed method identifies five high-risk tasks, eight medium-risk tasks, and fourteen low-risk tasks. These results are largely consistent with the logic of design review iterations and prototype modifications driven by Class-A surface smoothness requirements, aerodynamic testing, and related engineering constraints in the exterior styling phase. Therefore, the method enables an effective comprehensive assessment of risk in automotive styling design tasks.

Future research direction: following the identification of styling design task risks, future work will investigate how to reasonably schedule design tasks by integrating risk magnitude into project progress management.

References

[1] Wu, G. J. (2005). Rhythm and harmony in automotive styling design. Zhuangshi (Art & Design), (11), 110–111.

[2] Ranscombe, C., Kinsella, P., & Blijlevens, J. (2017). Data-driven styling: augmenting intuition in the product design process using holistic styling analysis. Journal of Mechanical Design, 139(11), 111417.

[3] Luos, M., Yi, P., Liu, Y., Tang, H., Zhang, J., Shao, J., Shi, J., & Jl, Z. (2025). Automobile styling design process reengineering method driven by intelligent technology. Computer Integrated Manufacturing Systems, CIMS, 31(9), 3113–3122.

[4] Ribeiro, A. S., deCastro, M., Costoya, X., Rusu, L., Dias, J. M., & Gomez-Gesteira, M. (2021). A Delphi method to classify wave energy resource for the 21st century: Application to the NW Iberian Peninsula. Energy, 235.

[5] Larkin, P., Leiss, W., & Krewski, D. (2019). Risk assessment and management frameworks for carbon capture and geological storage: a global perspective. International Journal of Risk Assessment and Management, 22(3-4), 254-285.
[6] Yang, R., & Deng, Y. (2021). Analysis on Security Risks in Tunnel Construction Based on the Fault Tree Analysis. IOP Conference Series: Earth and Environmental Science, 638(1).
[7] Yousefi, S., Alizadeh, A., Hayati, J., & Baghery, M. (2018). HSE risk prioritization using robust DEA-FMEA approach with undesirable outputs: A study of automotive parts industry in Iran. Safety Science, 102, 144–158.
[8] Li, B. W., Lü, K., Ji, Z. Y., et al. (2016). Estimation and Optimization of Project Management Risk Factors Based on the AHP—Taking Car Parts Project as an Example. Science and Technology Management Research, 36(2), 218–223.
[9] Mzougui, I., Carpitella, S., Certa, A., El Felsoufi, Z., & Izquierdo, J. (2020). Assessing Supply Chain Risks in the Automotive Industry through a Modified MCDM-Based FMECA.Processes,8(5), 579.
[10]Hu, M. T. (2023). Risk Identification and Evaluation of Automobile Supply Chain Based on Bayesian Network. Logistics Technology, 42(8), 137–142, 148.
[11]Li, Y. L., Pan, C. X., & Cai, Q. (2023). A Risk Evaluation Study of Car Design Development Based on Fuzzy Analytic Hierarchy Proces. Journal of Tongji University (Natural Science), 51(11), 1771–1774.
[12]Ju, Q., Liu, W., & Ruan, J. (2021). Evaluation of Vehicle Body Styling Based on AHP and EWM Method. Proceedings - 2021 3rd International Conference on Artificial Intelligence and Advanced Manufacture, AIAM 2021, 461–467.
[13]Zhang, G. F., & Wang, R. (2015). Risk Evaluation Model of Automotive Supplier Based on WBS-RBS. Journal of Wuhan University of Technology (Information & Management Engineering), 37(6), 803–807.
[14]Wu, P., Lin, F., Huang, J., & Xu, Y. (2024). Multiscale evaluation of tunnel construction safety risk: a case study of an offshore tunnel construction in Ningbo. Journal of Engineering and Applied Science, 71(1), 108.
[15]Zhou, H. B., Gao, W. J., Cai, L. B., & Zhang, H. (2009). Risk identification and analysis of subway foundation pit by using fault tree analysis method based on WBS-RBS. Rock and Soil Mechanics, 30(9), 2703-2707.
[16]Xiang, Y., Wu, Q., & Zhang, T. (2010). The risk assessment of bridge design based on the AHP-FCE model. China Civil Engineering Journal, 43, 275-280.
[17]Lei, M., Wang, Y. Q., & Wu, L. (2025). Research on the Risk Assessment of Highway Survey and Design based on Integrating AHP and Cloud Model. Highway, 70(2), 256–262.
[18]Hyun, K. C., Min, S., Choi, H., Park, J., & Lee, I. M. (2015). Risk analysis using fault-tree analysis (FTA) and analytic hierarchy process (AHP) applicable to shield TBM tunnels. Tunnelling and underground space technology, 49, 121-129.
[19]Pour, F. S. A., & Gheorghe, A. (2017). Risk assessment of oil and natural gas drilling process by employing fuzzy sets and analytical hierarchy process (AHP). 2017 International Annual Conference of the American Society for Engineering Management, ASEM 2017.
[20]Chen, G. H., Wu, W. S., Xu, S. Y., & Liu, K. (2013). Assessment HSE risk of during sea-crossing bridges project construction based on WBS-RBS and AHP. China Safety Science Journal, 23(9), 51-57.
[21]Dong, S., Li, S., Yu, F., & Wang, K. (2023). Risk Assessment of Immersed Tube Tunnel Construction. Processes, 11(4).
[22]Chen, H. J., Liu, B., & Ren, X. (2015). Key risks identification of management informatization projects construction for coal enterprises based on improved WBS-RBS and FHAP method. China Coal, 41(6), 30–37.
[23]Ji T, Liu J-W, Li Q-F. Safety risk evaluation of large and complex bridges during construction based on the Delphi-improved FAHP-factor analysis method. Adv Civ Eng. 2022;2022(1):5397032.
[24]Kubota, F. I., Cauchick-Miguel, P. A., Hsuan, J., & Lacerda, D. P. (2022). Modularity in design and production relationships: a field study in two automakers. International Journal of Advanced Manufacturing Technology, 123(5–6), 1589–1606.
[25]Qin, Y. (2015). Automobile Headlamp Modeling Design Analysis Based on Brand Design. Packaging Engineering, 36(18), 87–91.
[26]Fu, T., Li, X., Xu, W., Wang, J., Zhang, L., Ye, L., & Yu, R. (2024). Risk analysis of bridge maintenance accidents: A two-stage LEC method and Bayesian network approach. International Journal of Transportation Science and Technology, 15, 51–64.
[27]Zhao, Y., & Liu, Y. (2013). Research on subjective evaluation indexes for vehicle handling stability based on improved analytic hierarchy process and fuzzy comprehensive evaluation. China Mechanical Engineering, 24(18), 2519.
[28]Das, A., Kumar, V., & Dutta, S. (2024). A fuzzy-based AHP-VIKOR framework for risk analysis of safety-critical systems: A case study of nuclear power plant. Annals of Nuclear Energy, 209.

Statements and Declarations

Acknowledgments The authors also would like to sincerely thank the reviewers for their valuable comments on this work.

Author contribution All authors contributed to the study conception and design. **Jiefeng Lyu:** Conceptualization, Methodology. **Ganshu Sheng:** Software, Writing-original draft, Funding acquisition. **Zihao Wang:** Visualization, Investigation.

Conflict of interest The authors declare no competing interests.
