# Supplementary material for: Risk identification method for automotive styling design tasks based on an improved FAHP-VIKOR approach
Source: PLoS One. 2026 Jul 6;21(7):e0352278. doi: 10.1371/journal.pone.0352278 (PMC13336177; doi:10.1371/journal.pone.0352278)
Supplement: Attachment — Submitted filename: Responses to reviewer comments - PONE-D-25-67607.docx [file pone.0352278.s006.docx]

Dear Editor and Reviewers,

Thank you for your letter and for the reviewers’ comments concerning our manuscript entitled “Risk Identification Method for Automotive Styling Design Tasks Based on an Improved FAHP-VIKOR Approach”(**Manuscript Number: PONE-D-25-67607**).

Those comments are all valuable and very helpful for revising and improving our paper, as well as the important guiding significance to our research. We have studied the comments carefully and have made corrections which we hope meet with approval. Revised portions are marked in yellow in the manuscript. The main corrections in the paper and the responses to the reviewers’ comments are as follows.

**List of Responses**

## Journal Requirements

Journal Requirements:

When submitting your revision, we need you to address these additional requirements.

1. Please ensure that your manuscript meets PLOS ONE’s style requirements, including those for file naming. The PLOS ONE style templates can be found at

<https://journals.plos.org/plosone/s/file?id=wjVg/PLOSOne_formatting_sample_main_body.pdf> and
<https://journals.plos.org/plosone/s/file?id=ba62/PLOSOne_formatting_sample_title_authors_affiliations.pdf>

**Response:** The entire manuscript has been revised in strict accordance with PLOS ONE’s formatting and file naming guidelines (including heading hierarchy, figure/table citation and naming, author/affiliation formatting, etc.). All files (main manuscript, figures, supporting information) have been renamed to meet the journal’s specifications, and style consistency is ensured throughout the text.

2. Please note that PLOS One has specific guidelines on code sharing for submissions in which author-generated code underpins the findings in the manuscript. In these cases, we expect all author-generated code to be made available without restrictions upon publication of the work.

Please review our guidelines at <https://journals.plos.org/plosone/s/materials-and-software-sharing#loc-sharing-code> and ensure that your code is shared in a way that follows best practice and facilitates reproducibility and reuse.

**Response:** All author-generated code underpinning the study’s findings has been supplemented and submitted in the Supporting Information files of this revision. Meanwhile, we have clearly stated the details of the code (including its purpose and access method within the Supporting Information) in the manuscript’s Data Availability Statement, fully complying with PLOS ONE’s guidelines on code sharing.

3. We note that the grant information you provided in the ‘Funding Information’ and ‘Financial Disclosure’ sections do not match.

When you resubmit, please ensure that you provide the correct grant numbers for the awards you received for your study in the ‘Funding Information’ section.

**Response:** We have thoroughly reviewed the actual research work and re-supplemented and updated the Funding Information section based on the actual funding support received during the study. We have cross-checked it with the ‘Financial Disclosure’ section to ensure full consistency and accuracy.

4. We note that your Data Availability Statement is currently as follows:

“All relevant data are within the manuscript and its Supporting Information files.”

Please confirm at this time whether or not your submission contains all raw data required to replicate the results of your study. Authors must share the “minimal data set” for their submission. PLOS defines the minimal data set to consist of the data required to replicate all study findings reported in the article, as well as related metadata and methods (<https://journals.plos.org/plosone/s/data-availability#loc-minimal-data-set-definition)>.

For example, authors should submit the following data:

- The values behind the means, standard deviations and other measures reported;
- The values used to build graphs;
- The points extracted from images for analysis.

Authors do not need to submit their entire data set if only a portion of the data was used in the reported study.

If your submission does not contain these data, please either upload them as Supporting Information files or deposit them to a stable, public repository and provide us with the relevant URLs, DOIs, or accession numbers. For a list of recommended repositories, please see <https://journals.plos.org/plosone/s/recommended-repositories>.

If there are ethical or legal restrictions on sharing a de-identified data set, please explain them in detail (e.g., data contain potentially sensitive information, data are owned by a third-party organization, etc.) and who has imposed them (e.g., an ethics committee). Please also provide contact information for a data access committee, ethics committee, or other institutional body to which data requests may be sent. If data are owned by a third party, please indicate how others may request data access.

**Response:** We confirm that the complete minimal data set required to replicate all the study’s findings has been newly supplemented and submitted in the Supporting Information files of this revision. All data are organized in field-standard, machine-readable file formats to ensure reproducibility and reuse, with no ethical or legal restrictions on the sharing of these data. The Data Availability Statement in the manuscript has been updated to reflect this supplementary submission.

5. If the reviewer comments include a recommendation to cite specific previously published works, please review and evaluate these publications to determine whether they are relevant and should be cited. There is no requirement to cite these works unless the editor has indicated otherwise.

**Response:** Relevant and substantively related studies have been added to the manuscript’s reference list with consistent PLOS ONE citation formatting.

## Reviewer #1

1. Refine the research gap and contribution claims.

**Response:** Thanks for your suggestion. The Introduction section has been improved by clarifying the research gaps in traditional risk identification methods, including WBS-RBS and the analytic hierarchy process (AHP). In addition, the research contributions of the improved FAHP-VIKOR method proposed in this study have been explicitly stated. Specifically: (a) a WBS-RBS risk matrix aligned with the automotive styling design process has been constructed to clearly describe the coupling relationship between design tasks and risk factors. and (b) an improved FAHP-VIKOR risk quantification framework has been proposed to accurately classify and rank the risk levels of automotive styling design tasks.

2. Beef up the case study discussion and managerial implications.

**Response:** Thanks for your suggestion. In the revised manuscript, additional discussion and management implications regarding the case study of risk identification for automotive styling design tasks have been incorporated in Section 5, as detailed in Sections 5.3 and 5.4.

With respect to the case study discussion, a more in-depth analysis of the risk causes associated with high-, medium-, and low-risk nodes has been added, clarifying the key risk factors and underlying mechanisms affecting different types of risk nodes.

Regarding management implications, the specific management measures corresponding to different risk levels have been further refined to enhance their practical applicability.

3. Make clear the novelty and limits of the improved FAHP–VIKOR framework.

**Response:** Thanks for your suggestion. In the Introduction section of the revised manuscript, the novelty and limitations of the proposed improved FAHP-VIKOR framework have been explicitly clarified.The main contribution lies in the integration of the improved FAHP method with the VIKOR approach. The improved FAHP is employed to obtain more reliable weights of risk factors, while the VIKOR method introduces a compromise decision-making mechanism that balances group utility and individual regret. This integration enables a risk ranking of automotive styling design tasks that is more consistent with practical engineering conditions.

In addition, the limitations of the proposed method are also discussed. Specifically, the determination of risk occurrence probability and loss severity in the risk matrix still relies on expert scoring. Although the potential bias is mitigated through multi-expert evaluation and iterative revision, the influence of subjectivity cannot be completely eliminated.

4. The idea of an improved FAHP needs more explanation. The paper should explain better how it is better than normal FAHP methods.

**Response:** Thanks for your suggestion. In Section 4.1 of the revised manuscript, the procedure of the improved FAHP method has been further clarified and explained. In addition, its advantages over the conventional FAHP approach are explicitly discussed. Specifically, the improved FAHP method addresses some limitations of the traditional FAHP, such as the complexity of the consistency verification process and the high sensitivity of the results.

5. The paper needs to explain why VIKOR was chosen over other similar methods (like TOPSIS or PROMETHEE), and give reasons based on theory or real-world use.

**Response:** Thanks for your suggestion. In Section 4.2 of the revised manuscript, the theoretical rationale for selecting the VIKOR method has been further elaborated. VIKOR is based on the concept of a compromise solution, aiming to achieve a balance between maximizing group utility and minimizing individual regret. This mechanism highlights the influence of the most unfavorable factors, which correspond to critical risk constraints. Such a feature is highly consistent with the characteristics of risks in automotive styling design, where certain factors, such as regulatory compliance and engineering feasibility, often act as decisive constraints that cannot be compensated for by the advantages of other indicators. The individual regret measure in VIKOR is particularly effective in capturing the impact of these critical risk factors.

Moreover, by incorporating the individual regret measure, VIKOR prevents the dominance of a single advantageous indicator from fully offsetting serious deficiencies. The method also allows the decision-making coefficient *v* to be adjusted to represent different risk preference scenarios, thereby providing better interpretability and practical applicability.

6. The study uses expert opinions a lot to score and weigh risks, which can be subjective. It would be good to talk about how experts were chosen, how bias was reduced, or how reliable the analysis is.

**Response:** Thanks for your suggestion. In Section 3.3 of the revised manuscript, additional details regarding the expert selection process have been provided, including the evaluation criteria, the number of experts, their professional experience, and their positions or titles.

First, unified evaluation standards and risk definitions were established in the study. The scoring criteria for risk occurrence probability (P) and loss severity (L) were clearly defined to ensure that all experts conducted the assessment based on a consistent standard.

Second, the expert panel covers three key domains: styling design, engineering interface coordination, and project management. This multidisciplinary composition ensures that risks can be evaluated from multiple perspectives, thereby improving the comprehensiveness and accuracy of the risk identification process. In addition, multiple rounds of discussion combined with averaging of expert scores were adopted to reduce potential subjective bias.

7. The example used is complete and realistic, but mostly just describes things. It would be better if it dug deeper into how the results help managers make choices or make projects better.

**Response:** Thanks for your suggestion. In Section 5 of the revised manuscript, a new subsection (Section 5.5) has been added to provide a more detailed discussion of the results. In addition, a Gantt chart is introduced to support managerial decision-making in the SUV styling design project. The risk identification results provide managers with a clear localization of potential risks, enabling them to focus on critical task nodes. The five high-risk nodes W_12_, W_13_, W_14_, W_26_, and W_34_ identified in the case study represent key stages that significantly influence project schedule, cost, and quality. Based on these results, managers can prioritize resource allocation, such as assigning additional design personnel, extending review cycles, and reserving contingency budgets.

For high-risk nodes, proactive control measures are recommended, including early cross-departmental validation and optimization of the review process. For medium-risk nodes, targeted mitigation strategies can be adopted, such as strengthening virtual simulation validation and clarifying cross-departmental interface responsibilities. For low-risk nodes, standardized process management can be applied, including modular design practices and the reuse of accumulated design experience.

## Reviewer #2

1. Please, by the end of the introduction highlight as separate paragraphs or subsections the motivation, research gap, and the contribution.

**Response:** Thanks for your suggestion. In the Introduction section of the revised manuscript, separate paragraphs have been added to further clarify the research gap, research contributions, and the main innovations of this study.

2. “For these reasons, an improved FAHP [27] is employed to determine risk weights. By transforming the reciprocal matrix into a fuzzy consistency judgment matrix” . The idea is not clear from step 2. What is the relation between this step and accounting for fuzziness? How can this method avoid the flaw in the FAHP? More explanation is needed.

**Response:** Thanks for your suggestion. In Section 4.1, Step 2 of the improved FAHP procedure has been further elaborated. The primary objective of this step is to construct a consistency judgment matrix. This formulation avoids the strict consistency verification required in the traditional AHP method and overcomes the limitation of the classical FAHP approach that relies on iterative eigenvector computation. Instead, the weight vector is obtained by directly calculating the norm of each row vector in the matrix, which effectively reduces computational complexity.

Accordingly, Step 2 does not involve fuzzy processing, as the treatment of fuzziness has already been completed in Step 1 during the construction of the judgment matrix. In the original manuscript, the descriptions of Step 1 and Step 2 contained certain inconsistencies. These issues have now been corrected, and the corresponding revisions are highlighted in yellow in the manuscript.

3. Step 5 is also not clear. Why do we need this step? What is the relation between the infinity norm of a vector and a max eigenvalue?

**Response:** Thanks for your suggestion. In Section 4.1 of the revised manuscript, Step 5 of the improved FAHP procedure has been further explained in detail. For a non-negative consistent matrix, the sum of the elements in each row vector (i.e., the infinity norm) can reflect the relative importance of the corresponding indicator with respect to other indicators, and its direction is consistent with that of the principal eigenvector of the matrix. According to the Perron-Frobenius theorem, the largest eigenvalue of a positive matrix corresponds to a unique positive eigenvector. Therefore, normalization based on the infinity norm can approximate the principal eigenvector without explicitly solving the eigenvalue problem, thereby enabling efficient weight calculation.

The vector infinity norm $\left\| V_{k+1} \right\|_{\infty}$ refers to the element with the largest absolute value in the vector $V_{k+1}$. Its relationship with the maximum eigenvalue $\lambda_{max}$ is as follows: when the reciprocal matrix E satisfies the consistency condition, repeated iterations of the vector $V_{k+1}$ lead its infinity norm $\left\| V_{k+1} \right\|_{\infty}$ to converge to the maximum eigenvalue $\lambda_{max}$ of matrix E.

4. “The expert panel” nothing is mentioned about the experts. How many, their experience, their positions, etc.

**Response:** Thanks for your suggestion. In addition, Section 3.3 has been supplemented with detailed information regarding the expert selection process, including the evaluation criteria, number of experts, professional experience, and positions or titles.

The expert panel consists of seven members from well-known domestic automobile manufacturers and related supporting departments involved in automotive styling design. The selection criteria were mainly based on professional experience, relevance of job responsibilities, and participation in vehicle development projects. Specifically, the panel includes experts from three key areas: styling design, styling–engineering interface coordination, and project management. Among them, three are exterior or interior styling design specialists with 8-15 years of vehicle development experience; two are styling–engineering interface experts familiar with engineering feasibility and manufacturing constraints; and the remaining two are product project managers or styling management personnel.

5. More insights must be given in section 5.3.

**Response:** Thanks for your suggestion. In Section 5.3 of the revised manuscript, a more detailed analysis of the risk identification results for the SUV styling design project has been provided.

The results indicate that there are five high-risk nodes, nine medium-risk nodes, and thirteen low-risk nodes. High-risk nodes are mainly concentrated in the front-end styling, lidar integration area, and rear bumper region, where risks arise from the combined influence of technical challenges, schedule pressure, regulatory constraints, and cross-departmental coordination. Medium-risk nodes are relatively more dispersed and are typically associated with localized issues caused by one or a limited number of risk factors, such as technical feasibility, cost considerations, or coordination problems. Overall, styling aggressiveness, engineering feasibility, regulatory requirements, and cross-departmental collaboration are identified as the primary drivers leading to task rework, schedule delays, and cost increases.

6. Recommendations and managerial implications should be added.

**Response:** Thanks for your suggestion. In addition, a new subsection (Section 5.5) has been added to further discuss the results in detail, and a Gantt chart is introduced to support managerial decision-making in the SUV styling design project.

The risk identification results provide managers with clear risk localization, enabling them to focus on critical task nodes. The five high-risk nodes W12, W13, W14, W26, and W34 identified in the case study represent key stages that significantly influence project schedule, cost, and quality. Based on these findings, managers can prioritize resource allocation, such as assigning additional design personnel, extending review cycles, and reserving contingency budgets.

For high-risk nodes, proactive management measures can be implemented, including early cross-departmental validation and optimization of review procedures. For medium-risk nodes, targeted mitigation strategies may be adopted, such as strengthening virtual simulation validation and clarifying cross-departmental interface responsibilities. For low-risk nodes, standardized process management can be applied, including modular design practices and the reuse of accumulated design experience.

## Reviewer #3

1. does not clearly state what new insight or advantage is achieved compared to existing methods.

· No quantitative or qualitative result is highlighted

**Response:** Thanks for your suggestion. We have revised the Abstract to further emphasize the comparison with existing methods, including the standalone FAHP and FAHP-TOPSIS approaches, and have added qualitative analysis. We have highlighted the advantages of the proposed risk identification approach: the full-process WBS-RBS matrix and improved FAHP-VIKOR framework, which successfully identifies high-risk nodes such as the front bumper, LiDAR surface, and rear bumper. This method provides valuable decision support for risk management and resource optimization in automotive styling design projects.

2. research gap not strong

does not sufficiently explain why VIKOR is superior to other ranking methods

**Response:** Thanks for your suggestion. In Section 1 , we have strengthened the description of the research gap. Additionally, in Section 4.2, we have provided further explanation regarding the choice of the VIKOR method. Compared with TOPSIS, VIKOR avoids masking critical risks; relative to PROMETHEE, it is more stable and practical; and compared with single-weighted methods, it offers higher accuracy. Moreover, VIKOR is well suited to capturing the dynamic characteristics of risk in automotive styling design tasks.

3. limited conceptual explanation

**Response:** Thanks for your suggestion. In Section 2, Figure 1 provides an overview of the overall workflow of the proposed improved FAHP-VIKOR method. All the terms and concepts presented in the figure have been elaborated in Section 4.1 on the determination of risk weights using the improved FAHP method and in Section 4.2 on risk quantification and ranking using the VIKOR method. The key concepts are also explained in detail within each procedural step to ensure clarity and ease of understanding.

4. The rationale for choosing five stages is not theoretically justified.

· No discussion on alternative WBS structures or validation by industry experts.

**Response:** Thanks for your suggestion. In this study, the work breakdown structure for automotive styling design is divided into five core stages: project initiation and preparation, conceptual design and exploration, detailed design and digital model development, physical validation and review, and final delivery and archiving. The theoretical support for this division comes from two main aspects. First, as a complete project, automotive styling design follows the project lifecycle of initiation, planning, execution, control, and closure. The five stages correspond to different phases of the project lifecycle, covering the entire process from preliminary preparation to final delivery. Second, these stages are informed by the CATARC styling process guidelines and the styling design workflows of leading domestic and international automakers such as Dongfeng, SAIC, and Geely. They represent the core processes of automotive styling design, comprehensively covering key activities and aligning with industry standards.

The WBS structure used in this study consists of four layers: overall objective, five stages, sub-tasks, and work packages. By combining the characteristics of the styling design process with the physical decomposition of the vehicle structure, this structure accounts for both procedural and physical aspects, making it practical and well-suited to engineering practice.

5. ·	Risk indicators are experience-driven, but expert selection and validation are not described.

· Overlap between some risk categories is missing

**Response:** Thanks for your suggestion. In Section 3.3, we have added details regarding the selection of experts, scoring criteria, number of experts, professional experience, and positions. A total of seven experts from well-known automotive companies were selected, covering the areas of styling design, engineering interfaces, and project management, with five to fifteen years of relevant experience.

Regarding the issue of overlapping risk categories, some categories, such as technical risk and external compliance risk, do intersect. The expert panel clarified the core boundaries of each category and assigned overlapping risk factors to their primary category. To minimize the impact of overlapping risks on the evaluation results, during the construction of the WBS-RBS matrix, experts conducted focused discussions to cross-check the scoring results, identify potential duplicate evaluations or logical conflicts between different risk factors, and appropriately adjust the scores based on the underlying risk mechanisms.

6. The assumption that P and L can be multiplied linearly is not justified

**Response:** Thanks for your suggestion. In Section 3.3, we have added justification for the linear multiplication of probability (P) and loss (L). According to system safety engineering and the LEC evaluation method, the magnitude of a risk depends on both the probability of occurrence and the severity of potential loss. Considering the characteristics of risks in automotive styling design, P and L are treated as independent core indicators, and their linear multiplication provides a simple and accurate way to quantify the risk level of an individual task, with the risk level calculated as the product of probability and loss (Risk = P×L).

7. The improvement over classical FAHP is not fundamentally new in the area.

· Mathematical notation is complex and not suitable for non-technical readers.

**Response:** Thanks for your suggestion. In Section 4.1, we have added further clarification. The core contribution of the improved FAHP lies in its scenario-adaptive optimization rather than a fundamental mathematical innovation. By constructing a fuzzy consistency matrix and using the vector infinity norm to calculate weights, it addresses the limitations of traditional FAHP in automotive styling design, where subjective and fuzzy evaluations often make consistency checks cumbersome and weights unstable, thereby enhancing the practical applicability of the method in engineering. To address the complexity of mathematical symbols, we have added explanatory notes and provided plain-language explanations of the core computational logic.

8. The choice of v = 0.5 is not justified.

· No comparison with results from another MCDM method.

**Response:** Thanks for your suggestion. The parameter v in the VIKOR method serves as the decision-making coefficient, balancing group utility and individual regret. A value of 0.5 represents a balanced decision preference, which aligns with the core requirement of automotive styling design to manage overall risk while addressing critical weak points. This value is also consistent with commonly used settings in similar engineering risk assessment studies and can most accurately reflect the actual risk ranking needs in styling design.

In addition, we have supplemented comparative experiments with the FAHP-TOPSIS method and the standalone FAHP method. The results show that the proposed method achieves higher consistency with practical engineering risk levels, highlighting its superiorit

9. · The case study is descriptive not explain analytically

· No comparison with:

·	Traditional risk identification results.

·	Managerial decisions before/after applying the model.

**Response:** Thanks for your suggestion. In Section 5.3, we have strengthened the case analysis by providing additional explanation of the causes and impact mechanisms of high, medium, and low risk nodes. Combined with the quantitative logic of the improved FAHP-VIKOR method, this clarifies the basis for risk level classification.

In Section 5.4, we have added comparisons with traditional risk identification methods, including FAHP-TOPSIS and standalone FAHP. The results show that the proposed method accurately identifies high-risk nodes while avoiding subjective bias associated with expert judgment.

In Section 5.5, we have supplemented recommendations for project managers following risk identification. For high-risk nodes, managers can implement proactive control measures, such as early cross-department validation and optimized review processes. For medium-risk nodes, localized optimization and targeted preventive actions can be applied, including enhanced virtual simulation verification and clearly defined cross-department responsibilities. For low-risk nodes, standard process management can be adopted, with modular design and reuse of prior experience.

10. Conclusions mainly repeat the methodology rather than synthesize the insights.

**Response:** Thanks for your suggestion. We have also optimized Section 6, the conclusion, emphasizing the key research insights, which include:

1.Clarifying the distribution characteristics and core causes of high-risk nodes in automotive styling design;

2.Verifying the applicability and superiority of the improved FAHP-VIKOR framework in quantifying styling design risks;

3.Proposing a risk management strategy focused on classified control and precise interventions;

4.Identifying the limitations of the study and directions for future research.

Special thanks to you for your comments.

At last, we tried our best to improve the manuscript and made some changes in the manuscript. These changes will not influence the content and framework of the paper. And we marked them in yellow in the revised paper.

We appreciate for Editor’s and Reviewers’ warm work earnestly and hope that the correction will meet with approval.

Once again, thank you very much for your comments and suggestions.

Yours sincerely,

Ganshu Sheng (Corresponding Author)

shenggs@hbut.edu.cn
