# Supplementary material for: Risk identification method for automotive styling design tasks based on an improved FAHP-VIKOR approach
Source: PLoS One. 2026 Jul 6;21(7):e0352278. doi: 10.1371/journal.pone.0352278 (PMC13336177; doi:10.1371/journal.pone.0352278)
Supplement: Peer review history

# Peer review: Decision Letter 0

**DOI:** 10.1371/journal.pone.0352278.r001

**Authors:** Mithilesh K. Dikshit

28 Jan 2026

PONE-D-25-67607

Risk Identification Method for Automotive Styling Design Tasks Based on an Improved FAHP–VIKOR Approach

PLOS One

Dear Dr. Sheng,

Thank you for submitting your manuscript to PLOS ONE. After careful consideration, we feel that it has merit but does not fully meet PLOS ONE’s publication criteria as it currently stands. Therefore, we invite you to submit a revised version of the manuscript that addresses the points raised during the review process.

Please submit your revised manuscript by Mar 14 2026 11:59PM. If you will need more time than this to complete your revisions, please reply to this message or contact the journal office at plosone@plos.org. When you're ready to submit your revision, log on to https://www.editorialmanager.com/pone/ and select the 'Submissions Needing Revision' folder to locate your manuscript file.

Please include the following items when submitting your revised manuscript:

- A letter that responds to each point raised by the academic editor and reviewer(s). You should upload this letter as a separate file labeled 'Response to Reviewers'.
- A marked-up copy of your manuscript that highlights changes made to the original version. You should upload this as a separate file labeled 'Revised Manuscript with Track Changes'.
- An unmarked version of your revised paper without tracked changes. You should upload this as a separate file labeled 'Manuscript'.

If you would like to make changes to your financial disclosure, please include your updated statement in your cover letter. Guidelines for resubmitting your figure files are available below the reviewer comments at the end of this letter.

If applicable, we recommend that you deposit your laboratory protocols in protocols.io to enhance the reproducibility of your results. Protocols.io assigns your protocol its own identifier (DOI) so that it can be cited independently in the future. For instructions see: https://journals.plos.org/plosone/s/submission-guidelines#loc-laboratory-protocols. Additionally, PLOS ONE offers an option for publishing peer-reviewed Lab Protocol articles, which describe protocols hosted on protocols.io. Read more information on sharing protocols at https://plos.org/protocols?utm_medium=editorial-email&utm_source=authorletters&utm_campaign=protocols.

We look forward to receiving your revised manuscript.

Kind regards,

Mithilesh K. Dikshit

Academic Editor

PLOS One

Journal Requirements:

When submitting your revision, we need you to address these additional requirements.

1. Please ensure that your manuscript meets PLOS ONE's style requirements, including those for file naming. The PLOS ONE style templates can be found at

https://journals.plos.org/plosone/s/file?id=wjVg/PLOSOne_formatting_sample_main_body.pdf and

https://journals.plos.org/plosone/s/file?id=ba62/PLOSOne_formatting_sample_title_authors_affiliations.pdf

2. Please note that PLOS One has specific guidelines on code sharing for submissions in which author-generated code underpins the findings in the manuscript. In these cases, we expect all author-generated code to be made available without restrictions upon publication of the work.

Please review our guidelines at https://journals.plos.org/plosone/s/materials-and-software-sharing#loc-sharing-code and ensure that your code is shared in a way that follows best practice and facilitates reproducibility and reuse.

3. We note that the grant information you provided in the ‘Funding Information’ and ‘Financial Disclosure’ sections do not match.

When you resubmit, please ensure that you provide the correct grant numbers for the awards you received for your study in the ‘Funding Information’ section.

4. We note that your Data Availability Statement is currently as follows:

“All relevant data are within the manuscript and its Supporting Information files.”

Please confirm at this time whether or not your submission contains all raw data required to replicate the results of your study. Authors must share the “minimal data set” for their submission. PLOS defines the minimal data set to consist of the data required to replicate all study findings reported in the article, as well as related metadata and methods (https://journals.plos.org/plosone/s/data-availability#loc-minimal-data-set-definition).

For example, authors should submit the following data:

- The values behind the means, standard deviations and other measures reported;

- The values used to build graphs;

- The points extracted from images for analysis.

Authors do not need to submit their entire data set if only a portion of the data was used in the reported study.

If your submission does not contain these data, please either upload them as Supporting Information files or deposit them to a stable, public repository and provide us with the relevant URLs, DOIs, or accession numbers. For a list of recommended repositories, please see https://journals.plos.org/plosone/s/recommended-repositories.

If there are ethical or legal restrictions on sharing a de-identified data set, please explain them in detail (e.g., data contain potentially sensitive information, data are owned by a third-party organization, etc.) and who has imposed them (e.g., an ethics committee). Please also provide contact information for a data access committee, ethics committee, or other institutional body to which data requests may be sent. If data are owned by a third party, please indicate how others may request data access.

5. If the reviewer comments include a recommendation to cite specific previously published works, please review and evaluate these publications to determine whether they are relevant and should be cited. There is no requirement to cite these works unless the editor has indicated otherwise.

**Additional Editor Comments:**

Revised the manuscript as per the reviewers ' comments.

[Note: HTML markup is below. Please do not edit.]

Reviewers' comments:

Reviewer's Responses to Questions

**Comments to the Author**

1. Is the manuscript technically sound, and do the data support the conclusions?

The manuscript must describe a technically sound piece of scientific research with data that supports the conclusions. Experiments must have been conducted rigorously, with appropriate controls, replication, and sample sizes. The conclusions must be drawn appropriately based on the data presented.

Reviewer #1: Partly

Reviewer #2: Yes

**********

2. Has the statistical analysis been performed appropriately and rigorously?

Reviewer #1: No

Reviewer #2: N/A

**********

3. Have the authors made all data underlying the findings in their manuscript fully available?

The PLOS Data policy requires authors to make all data underlying the findings described in their manuscript fully available without restriction, with rare exception (please refer to the Data Availability Statement in the manuscript PDF file). The data should be provided as part of the manuscript or its supporting information, or deposited to a public repository. For example, in addition to summary statistics, the data points behind means, medians and variance measures should be available. If there are restrictions on publicly sharing data—e.g. participant privacy or use of data from a third party—those must be specified.

Reviewer #1: Yes

Reviewer #2: Yes

**********

4. Is the manuscript presented in an intelligible fashion and written in standard English?

PLOS ONE does not copyedit accepted manuscripts, so the language in submitted articles must be clear, correct, and unambiguous. Any typographical or grammatical errors should be corrected at revision, so please note any specific errors here.

Reviewer #1: Yes

Reviewer #2: Yes

**********

5. Review Comments to the Author

Please use the space provided to explain your answers to the questions above. You may also include additional comments for the author, including concerns about dual publication, research ethics, or publication ethics. (Please upload your review as an attachment if it exceeds 20,000 characters)

Reviewer #1: Refine the research gap and contribution claims.

Beef up the case study discussion and managerial implications.

Make clear the novelty and limits of the improved FAHP–VIKOR framework.

The idea of an improved FAHP needs more explanation. The paper should explain better how it is better than normal FAHP methods.

The paper needs to explain why VIKOR was chosen over other similar methods (like TOPSIS or PROMETHEE), and give reasons based on theory or real-world use.

The study uses expert opinions a lot to score and weigh risks, which can be subjective. It would be good to talk about how experts were chosen, how bias was reduced, or how reliable the analysis is.

The example used is complete and realistic, but mostly just describes things. It would be better if it dug deeper into how the results help managers make choices or make projects better.

Reviewer #2: 1. Please, by the end of the introduction highlight as separate paragraphs or subsections the motivation, research gap, and the contribution.

2. “For these reasons, an improved FAHP [27] is employed to determine risk weights. By transforming the reciprocal matrix into a fuzzy consistency judgment matrix” . The idea is not clear from step 2. What is the relation between this step and accounting for fuzziness? How can this method avoid the flaw in the FAHP? More explanation is needed.

3. Step 5 is also not clear. Why do we need this step? What is the relation between the infinity norm of a vector and a max eigenvalue?

4. “The expert panel” nothing is mentioned about the experts. How many, their experience, their positions, etc.

5. More insights must be given in section 5.3.

6. Recommendations and managerial implications should be added.

**********

6. PLOS authors have the option to publish the peer review history of their article (what does this mean?). If published, this will include your full peer review and any attached files.

If you choose “no”, your identity will remain anonymous but your review may still be made public.

**Do you want your identity to be public for this peer review?**  For information about this choice, including consent withdrawal, please see our Privacy Policy.

Reviewer #1: No

Reviewer #2: No

**********

[NOTE: If reviewer comments were submitted as an attachment file, they will be attached to this email and accessible via the submission site. Please log into your account, locate the manuscript record, and check for the action link "View Attachments". If this link does not appear, there are no attachment files.]

To ensure your figures meet our technical requirements, please review our figure guidelines: https://journals.plos.org/plosone/s/figures

You may also use PLOS’s free figure tool, NAAS, to help you prepare publication quality figures: https://journals.plos.org/plosone/s/figures#loc-tools-for-figure-preparation.

NAAS will assess whether your figures meet our technical requirements by comparing each figure against our figure specifications.

Attachment Submitted filename: PONE-D-25-67607.pdf

# Author comment: Author response to Decision Letter 1

**DOI:** 10.1371/journal.pone.0352278.r002

12 Mar 2026

Dear Editor and Reviewers,

Thank you for your letter and for the reviewers’ comments concerning our manuscript entitled “Risk Identification Method for Automotive Styling Design Tasks Based on an Improved FAHP-VIKOR Approach”(Manuscript Number: PONE-D-25-67607).

Those comments are all valuable and very helpful for revising and improving our paper, as well as the important guiding significance to our research. We have studied the comments carefully and have made corrections which we hope meet with approval. Revised portions are marked in yellow in the manuscript. The main corrections in the paper and the responses to the reviewers’ comments are as follows.

List of Responses

Reviewer #1

1. Refine the research gap and contribution claims.

Response: Thanks for your suggestion. The Introduction section has been improved by clarifying the research gaps in traditional risk identification methods, including WBS-RBS and the analytic hierarchy process (AHP). In addition, the research contributions of the improved FAHP-VIKOR method proposed in this study have been explicitly stated. Specifically: (a) a WBS-RBS risk matrix aligned with the automotive styling design process has been constructed to clearly describe the coupling relationship between design tasks and risk factors. and (b) an improved FAHP-VIKOR risk quantification framework has been proposed to accurately classify and rank the risk levels of automotive styling design tasks.

2. Beef up the case study discussion and managerial implications.

Response: Thanks for your suggestion. In the revised manuscript, additional discussion and management implications regarding the case study of risk identification for automotive styling design tasks have been incorporated in Section 5, as detailed in Sections 5.3 and 5.4.

With respect to the case study discussion, a more in-depth analysis of the risk causes associated with high-, medium-, and low-risk nodes has been added, clarifying the key risk factors and underlying mechanisms affecting different types of risk nodes.

Regarding management implications, the specific management measures corresponding to different risk levels have been further refined to enhance their practical applicability.

3. Make clear the novelty and limits of the improved FAHP–VIKOR framework.

Response: Thanks for your suggestion. In the Introduction section of the revised manuscript, the novelty and limitations of the proposed improved FAHP-VIKOR framework have been explicitly clarified.The main contribution lies in the integration of the improved FAHP method with the VIKOR approach. The improved FAHP is employed to obtain more reliable weights of risk factors, while the VIKOR method introduces a compromise decision-making mechanism that balances group utility and individual regret. This integration enables a risk ranking of automotive styling design tasks that is more consistent with practical engineering conditions.

In addition, the limitations of the proposed method are also discussed. Specifically, the determination of risk occurrence probability and loss severity in the risk matrix still relies on expert scoring. Although the potential bias is mitigated through multi-expert evaluation and iterative revision, the influence of subjectivity cannot be completely eliminated.

4. The idea of an improved FAHP needs more explanation. The paper should explain better how it is better than normal FAHP methods.

Response: Thanks for your suggestion. In Section 4.1 of the revised manuscript, the procedure of the improved FAHP method has been further clarified and explained. In addition, its advantages over the conventional FAHP approach are explicitly discussed. Specifically, the improved FAHP method addresses some limitations of the traditional FAHP, such as the complexity of the consistency verification process and the high sensitivity of the results.

5. The paper needs to explain why VIKOR was chosen over other similar methods (like TOPSIS or PROMETHEE), and give reasons based on theory or real-world use.

Response: Thanks for your suggestion. In Section 4.2 of the revised manuscript, the theoretical rationale for selecting the VIKOR method has been further elaborated. VIKOR is based on the concept of a compromise solution, aiming to achieve a balance between maximizing group utility and minimizing individual regret. This mechanism highlights the influence of the most unfavorable factors, which correspond to critical risk constraints. Such a feature is highly consistent with the characteristics of risks in automotive styling design, where certain factors, such as regulatory compliance and engineering feasibility, often act as decisive constraints that cannot be compensated for by the advantages of other indicators. The individual regret measure in VIKOR is particularly effective in capturing the impact of these critical risk factors.

Moreover, by incorporating the individual regret measure, VIKOR prevents the dominance of a single advantageous indicator from fully offsetting serious deficiencies. The method also allows the decision-making coefficient v to be adjusted to represent different risk preference scenarios, thereby providing better interpretability and practical applicability.

6. The study uses expert opinions a lot to score and weigh risks, which can be subjective. It would be good to talk about how experts were chosen, how bias was reduced, or how reliable the analysis is.

Response: Thanks for your suggestion. In Section 3.3 of the revised manuscript, additional details regarding the expert selection process have been provided, including the evaluation criteria, the number of experts, their professional experience, and their positions or titles.

First, unified evaluation standards and risk definitions were established in the study. The scoring criteria for risk occurrence probability (P) and loss severity (L) were clearly defined to ensure that all experts conducted the assessment based on a consistent standard.

Second, the expert panel covers three key domains: styling design, engineering interface coordination, and project management. This multidisciplinary composition ensures that risks can be evaluated from multiple perspectives, thereby improving the comprehensiveness and accuracy of the risk identification process. In addition, multiple rounds of discussion combined with averaging of expert scores were adopted to reduce potential subjective bias.

7. The example used is complete and realistic, but mostly just describes things. It would be better if it dug deeper into how the results help managers make choices or make projects better.

Response: Thanks for your suggestion. In Section 5 of the revised manuscript, a new subsection (Section 5.5) has been added to provide a more detailed discussion of the results. In addition, a Gantt chart is introduced to support managerial decision-making in the SUV styling design project. The risk identification results provide managers with a clear localization of potential risks, enabling them to focus on critical task nodes. The five high-risk nodes W12, W13, W14, W26, and W34 identified in the case study represent key stages that significantly influence project schedule, cost, and quality. Based on these results, managers can prioritize resource allocation, such as assigning additional design personnel, extending review cycles, and reserving contingency budgets.

For high-risk nodes, proactive control measures are recommended, including early cross-departmental validation and optimization of the review process. For medium-risk nodes, targeted mitigation strategies can be adopted, such as strengthening virtual simulation validation and clarifying cross-departmental interface responsibilities. For low-risk nodes, standardized process management can be applied, including modular design practices and the reuse of accumulated design experience.

Reviewer #2

1. Please, by the end of the introduction highlight as separate paragraphs or subsections the motivation, research gap, and the contribution.

Response: Thanks for your suggestion. In the Introduction section of the revised manuscript, separate paragraphs have been added to further clarify the research gap, research contributions, and the main innovations of this study.

2. “For these reasons, an improved FAHP [27] is employed to determine risk weights. By transforming the reciprocal matrix into a fuzzy consistency judgment matrix” . The idea is not clear from step 2. What is the relation between this step and accounting for fuzziness? How can this method avoid the flaw in the FAHP? More explanation is needed.

Response: Thanks for your suggestion. In Section 4.1, Step 2 of the improved FAHP procedure has been further elaborated. The primary objective of this step is to construct a consistency judgment matrix. This formulation avoids the strict consistency verification required in the traditional AHP method and overcomes the limitation of the classical FAHP approach that relies on iterative eigenvector computation. Instead, the weight vector is obtained by directly calculating the norm of each row vector in the matrix, which effectively reduces computational complexity.

Accordingly, Step 2 does not involve fuzzy processing, as the treatment of fuzziness has already been completed in Step 1 during the construction of the judgment matrix. In the original manuscript, the descriptions of Step 1 and Step 2 contained certain inconsistencies. These issues have now been corrected, and the corresponding revisions are highlighted in yellow in the manuscript.

3. Step 5 is also not clear. Why do we need this step? What is the relation between the infinity norm of a vector and a max eigenvalue?

Response: Thanks for your suggestion. In Section 4.1 of the revised manuscript, Step 5 of the improved FAHP procedure has been further explained in detail. For a non-negative consistent matrix, the sum of the elements in each row vector (i.e., the infinity norm) can reflect the relative importance of the corresponding indicator with respect to other indicators, and its direction is consistent with that of the principal eigenvector of the matrix. According to the Perron-Frobenius theorem, the largest eigenvalue of a positive matrix corresponds to a unique positive eigenvector. Therefore, normalization based on the infinity norm can approximate the principal eigenvector without explicitly solving the eigenvalue problem, thereby enabling efficient weight calculation.

The vector infinity norm ‖V_(k+1) ‖_∞ refers to the element with the largest absolute value in the vector V_(k+1). Its relationship with the maximum eigenvalue λ_max is as follows: when the reciprocal matrix E satisfies the consistency condition, repeated iterations of the vector V_(k+1) lead its infinity norm ‖V_(k+1) ‖_∞ to converge to the maximum eigenvalue λ_max of matrix E.

4. “The expert panel” nothing is mentioned about the experts. How many, their experience, their positions, etc.

Response: Thanks for your suggestion. In addition, Section 3.3 has been supplemented with detailed information regarding the expert selection process, including the evaluation criteria, number of experts, professional experience, and positions or titles.

The expert panel consists of seven members from well-known domestic automobile manufacturers and related supporting departments involved in automotive styling design. The selection criteria were mainly based on professional experience, relevance of job responsibilities, and participation in vehicle development projects. Specifically, the panel includes experts from three key areas: styling design, styling–engineering interface coordination, and project management. Among them, three are exterior or interior styling design specialists with 8-15 years of vehicle development experience; two are styling–engineering interface experts familiar with engineering feasibility and manufacturing constraints; and the remaining two are product project managers or styling management personnel.

5. More insights must be given in section 5.3.

Response: Thanks for your suggestion. In Section 5.3 of the revised manuscript, a more detailed analysis of the risk identification results for the SUV styling design project has been provided.

The results indicate that there are five high-risk nodes, nine medium-risk nodes, and thirteen low-risk nodes. High-risk nodes are mainly concentrated in the front-end styling, lidar integration area, and rear bumper region, where risks arise from the combined influence of technical challenges, schedule pressure, regulatory constraints, and cross-departmental coordination. Medium-risk nodes are relatively more dispersed and are typically associated with localized issues caused by one or a limited number of risk factors, such as technical feasibility, cost considerations, or coordination problems. Overall, styling aggressiveness, engineering feasibility, regulatory requirements, and cross-departmental collaboration are identified as the primary drivers leading to task rework, schedule delays, and cost increases.

6. Recommendations and managerial implications should be added.

Response: Thanks for your suggestion. In addition, a new subsection (Section 5.5) has been added to further discuss the results in detail, and a Gantt chart is introduced to support managerial decision-making in the SUV styling design project.

The risk identification results provide managers with clear risk localization, enabling them to focus on critical task nodes. The five high-risk nodes W12, W13, W14, W26, and W34 identified in the case study represent key stages that significantly influence project schedule, cost, and quality. Based on these findings, managers can prioritize resource allocation, such as assigning additional design personnel, extending review cycles, and reserving contingency budgets.

For high-risk nodes, proactive management measures can be implemented, including early cross-departmental validation and optimization of review procedures. For medium-risk nodes, targeted mitigation strategies may be adopted, such as strengthening virtual simulation validation and clarifying cross-departmental interface responsibilities. For low-risk nodes, standardized process management can be applied, including modular design practices and the reuse of accumulated design experience.

Reviewer #3

1. does not clearly state what new insight or advantage is achieved compared to existing methods.

· No quantitative or qualitative result is highlighted

Response: Thanks for your suggestion. We have revised the Abstract to further emphasize the comparison with existing methods, including the standalone FAHP and FAHP-TOPSIS approaches, and have added qualitative analysis. We have highlighted the advantages of the proposed risk identification approach: the full-process WBS-RBS matrix and improved FAHP-VIKOR framework, which successfully identifies high-risk nodes such as the front bumper, LiDAR surface, and rear bumper. This method provides valuable decision support for risk management and resource optimization in automotive styling design projects.

2. research gap not strong

does not sufficiently explain why VIKOR is superior to other ranking methods

Response: Thanks for your suggestion. In Section 1 , we have strengthened the description of the research gap. Additionally, in Section 4.2, we have provided further explanation regarding the choice of the VIKOR method. Compared with TOPSIS, VIKOR avoids masking critical risks; relative to PROMETHEE, it is more stable and practical; and compared with single-weighted methods, it offers higher accuracy. Moreover, VIKOR is well suited to capturing the dynamic characteristics of risk in automotive styling design tasks.

3. limited conceptual explanation

Response: Thanks for your suggestion. In Section 2, Figure 1 provides an overview of the overall workflow of the proposed improved FAHP-VIKOR method. All the terms and concepts presented in the figure have been elaborated in Section 4.1 on the determination of risk weights using the improved FAHP method and in Section 4.2 on risk quantification and ranking using the VIKOR method. The key concepts are also explained in detail within each procedural step to ensure cl

Attachment Submitted filename: Responses to reviewer comments - PONE-D-25-67607.docx

# Peer review: Decision Letter 1

**DOI:** 10.1371/journal.pone.0352278.r003

**Authors:** Mithilesh K. Dikshit, Mithilesh K. Dikshit

8 Apr 2026

PONE-D-25-67607R1

Risk Identification Method for Automotive Styling Design Tasks Based on an Improved FAHP-VIKOR Approach

PLOS One

Dear Dr. Sheng,

Thank you for submitting your manuscript to PLOS ONE. After careful consideration, we feel that it has merit but does not fully meet PLOS ONE’s publication criteria as it currently stands. Therefore, we invite you to submit a revised version of the manuscript that addresses the points raised during the review process.

Please submit your revised manuscript by May 23 2026 11:59PM. If you will need more time than this to complete your revisions, please reply to this message or contact the journal office at plosone@plos.org. When you're ready to submit your revision, log on to https://www.editorialmanager.com/pone/ and select the 'Submissions Needing Revision' folder to locate your manuscript file.

Please include the following items when submitting your revised manuscript:

- A letter that responds to each point raised by the academic editor and reviewer(s). You should upload this letter as a separate file labeled 'Response to Reviewers'.
- A marked-up copy of your manuscript that highlights changes made to the original version. You should upload this as a separate file labeled 'Revised Manuscript with Track Changes'.
- An unmarked version of your revised paper without tracked changes. You should upload this as a separate file labeled 'Manuscript'.

If you would like to make changes to your financial disclosure, please include your updated statement in your cover letter. Guidelines for resubmitting your figure files are available below the reviewer comments at the end of this letter.

If applicable, we recommend that you deposit your laboratory protocols in protocols.io to enhance the reproducibility of your results. Protocols.io assigns your protocol its own identifier (DOI) so that it can be cited independently in the future. For instructions see: https://journals.plos.org/plosone/s/submission-guidelines#loc-laboratory-protocols. Additionally, PLOS ONE offers an option for publishing peer-reviewed Lab Protocol articles, which describe protocols hosted on protocols.io. Read more information on sharing protocols at https://plos.org/protocols?utm_medium=editorial-email&utm_source=authorletters&utm_campaign=protocols.

As the corresponding author, your ORCID iD is verified in the submission system and will appear in the published article. PLOS supports the use of ORCID, and we encourage all coauthors to register for an ORCID iD and use it as well. Please encourage your coauthors to verify their ORCID iD within the submission system before final acceptance, as unverified ORCID iDs will not appear in the published article. *Only*  the individual author can complete the verification step; PLOS staff *cannot*  verify ORCID iDs on behalf of authors.

We look forward to receiving your revised manuscript.

Kind regards,

Mithilesh K. Dikshit

Academic Editor

PLOS One

Journal Requirements:

If the reviewer comments include a recommendation to cite specific previously published works, please review and evaluate these publications to determine whether they are relevant and should be cited. There is no requirement to cite these works unless the editor has indicated otherwise.

Please review your reference list to ensure that it is complete and correct. If you have cited papers that have been retracted, please include the rationale for doing so in the manuscript text, or remove these references and replace them with relevant current references. Any changes to the reference list should be mentioned in the rebuttal letter that accompanies your revised manuscript. If you need to cite a retracted article, indicate the article’s retracted status in the References list and also include a citation and full reference for the retraction notice.

**Additional Editor Comments:**

The authors are required to revise the manuscript in accordance with the reviewers’ comments. If a reviewer has suggested citing their own research papers, inclusion of such references is not mandatory unless they are considered relevant and add significant value to the revised manuscript.

[Note: HTML markup is below. Please do not edit.]

Reviewers' comments:

Reviewer's Responses to Questions

**Comments to the Author**

1. If the authors have adequately addressed your comments raised in a previous round of review and you feel that this manuscript is now acceptable for publication, you may indicate that here to bypass the “Comments to the Author” section, enter your conflict of interest statement in the “Confidential to Editor” section, and submit your "Accept" recommendation.

Reviewer #2: All comments have been addressed

Reviewer #3: (No Response)

**********

2. Is the manuscript technically sound, and do the data support the conclusions?

The manuscript must describe a technically sound piece of scientific research with data that supports the conclusions. Experiments must have been conducted rigorously, with appropriate controls, replication, and sample sizes. The conclusions must be drawn appropriately based on the data presented.

Reviewer #2: Yes

Reviewer #3: Partly

**********

3. Has the statistical analysis been performed appropriately and rigorously?

Reviewer #2: N/A

Reviewer #3: No

**********

4. Have the authors made all data underlying the findings in their manuscript fully available?

The PLOS Data policy requires authors to make all data underlying the findings described in their manuscript fully available without restriction, with rare exception (please refer to the Data Availability Statement in the manuscript PDF file). The data should be provided as part of the manuscript or its supporting information, or deposited to a public repository. For example, in addition to summary statistics, the data points behind means, medians and variance measures should be available. If there are restrictions on publicly sharing data—e.g. participant privacy or use of data from a third party—those must be specified.

Reviewer #2: Yes

Reviewer #3: Yes

**********

5. Is the manuscript presented in an intelligible fashion and written in standard English?

PLOS ONE does not copyedit accepted manuscripts, so the language in submitted articles must be clear, correct, and unambiguous. Any typographical or grammatical errors should be corrected at revision, so please note any specific errors here.

Reviewer #2: Yes

Reviewer #3: Yes

**********

6. Review Comments to the Author

Please use the space provided to explain your answers to the questions above. You may also include additional comments for the author, including concerns about dual publication, research ethics, or publication ethics. (Please upload your review as an attachment if it exceeds 20,000 characters)

Reviewer #2: (No Response)

Reviewer #3: This manuscript deals with development of Risk Identification Method for Automotive Styling Design Tasks Based on an Improved FAHP-VIKOR Approach. The manuscript is well organized, however have several shortcomings which needs adequate addressing. Some of my comments are below

1. Why author had chosen VIKOR method, there are several new MCDM methods are available. Authors need to add advantages of VIKOR over other MCDM.

2. Authors need to add adequate problem definition (it doesn't specify what makes current methods inadequate). Also. research gap is implied rather than explicitly articulated. Author need to carefully write down research gaps which is unclear right now.

3. What authors have improved here is unclear. FAHP already existed in literature VIKOR is also existing technique. Whether author claiming integration as improvement or any mathematical changes, must be specified clearly.

4. Authors are advised to add sensitivity analysis, which is mandatory for justifying the results.

5. In addition, authors need to compare the results with other FAHP based methods (atleast 2-3) for better understanding of results.

6. The introduction section seems weak and required to add qualitative literature in the context of MCDM methods in different applications as given below.

https://link.springer.com/article/10.1007/s12046-018-1020-x, https://link.springer.com/article/10.1007/s12633-020-00600-4, https://link.springer.com/article/10.1007/s12008-023-01528-w, https://doi.org/10.1002/pc.26097, https://journals.sagepub.com/doi/abs/10.1177/1464420720953447, https://www.nature.com/articles/s41598-025-16827-3, https://link.springer.com/article/10.1007/s12633-025-03374-9

7. How the authors have identified high risk nodes is unclear?

8. Authors must classify these high risk nodes in terms of design complexity, manufacturing constraints, or technological uncertainty (e.g., lidar integration) for better clarification.

9. Authors need to add some implications of this work with challenges and future work.

10. The authors need to work highly on linguistic and grammatical errors throughout the manuscript.

Best wishes

**********

7. PLOS authors have the option to publish the peer review history of their article (what does this mean?). If published, this will include your full peer review and any attached files.

If you choose “no”, your identity will remain anonymous but your review may still be made public.

**Do you want your identity to be public for this peer review?**  For information about this choice, including consent withdrawal, please see our Privacy Policy.

Reviewer #2: No

Reviewer #3: **Yes:**  Vimal Kumar Pathak

**********

[NOTE: If reviewer comments were submitted as an attachment file, they will be attached to this email and accessible via the submission site. Please log into your account, locate the manuscript record, and check for the action link "View Attachments". If this link does not appear, there are no attachment files.]

To ensure your figures meet our technical requirements, please review our figure guidelines: https://journals.plos.org/plosone/s/figures

You may also use PLOS’s free figure tool, NAAS, to help you prepare publication quality figures: https://journals.plos.org/plosone/s/figures#loc-tools-for-figure-preparation.

NAAS will assess whether your figures meet our technical requirements by comparing each figure against our figure specifications.

# Author comment: Author response to Decision Letter 2

**DOI:** 10.1371/journal.pone.0352278.r004

5 May 2026

Dear Editor and Reviewers,

Thank you for your letter and for the reviewers’ comments concerning our manuscript entitled “Risk Identification Method for Automotive Styling Design Tasks Based on an Improved FAHP-VIKOR Approach”(Manuscript Number: PONE-D-25-67607R1).

Those comments are all valuable and very helpful for revising and improving our paper, as well as the important guiding significance to our research. We have studied the comments carefully and have made corrections which we hope meet with approval. Revised portions are marked in yellow in the manuscript. The main corrections in the paper and the responses to the reviewers’ comments are as follows.

List of Responses

Journal Requirements

1.If the reviewer comments include a recommendation to cite specific previously published works, please review and evaluate these publications to determine whether they are relevant and should be cited. There is no requirement to cite these works unless the editor has indicated otherwise.

Response: Relevant and substantively related studies have been added to the manuscript’s reference list with consistent PLOS ONE citation formatting.

2.Please review your reference list to ensure that it is complete and correct. If you have cited papers that have been retracted, please include the rationale for doing so in the manuscript text, or remove these references and replace them with relevant current references. Any changes to the reference list should be mentioned in the rebuttal letter that accompanies your revised manuscript. If you need to cite a retracted article, indicate the article’s retracted status in the References list and also include a citation and full reference for the retraction notice.

Response: We have carefully reviewed the entire reference list. No retracted papers have been cited in this manuscript. All references are complete and correct to the best of our knowledge. No changes have been made to the reference list.

3.Additional Editor Comments: The authors are required to revise the manuscript in accordance with the reviewers’ comments. If a reviewer has suggested citing their own research papers, inclusion of such references is not mandatory unless they are considered relevant and add significant value to the revised manuscript.

Response: We have carefully evaluated all publications suggested by the reviewers. Among them, we found that Yadav et al. [31] and Gangwar et al. [32] are highly relevant to our study, as they provide important comparative context for TOPSIS and MOORA methods in multi-criteria engineering applications. These two references have been added to the Introduction to strengthen our discussion on the limitations of fully compensatory MCDM methods and to better justify the selection of VIKOR for automotive styling risk identification.

The remaining suggested references were assessed and deemed less directly relevant to the specific focus of our article (i.e., risk identification for automotive styling design tasks). Therefore, they were not included, in accordance with the editorial guidance that such citations are not mandatory unless they add significant value to the revised article.

Reviewer #3:

This manuscript deals with development of Risk Identification Method for Automotive Styling Design Tasks Based on an Improved FAHP-VIKOR Approach. The manuscript is well organized, however have several shortcomings which needs adequate addressing. Some of my comments are below

1.Why author had chosen VIKOR method, there are several new MCDM methods are available. Authors need to add advantages of VIKOR over other MCDM.

Response: Thanks for your suggestion. In the revised manuscript, we have added a concise comparative discussion of mainstream MCDM methods (TOPSIS, PROMETHEE, and MOORA) and clarified the rationale for selecting the VIKOR method in the Introduction section.

Specifically, we point out that methods such as TOPSIS and MOORA are based on fully compensatory assumptions, which may weaken the influence of critical non-compensatory risk factors, while PROMETHEE is sensitive to preference function and threshold settings. In contrast, VIKOR emphasizes compromise solutions by balancing group utility and individual regret, making it more suitable for highlighting dominant risk factors in constraint-driven automotive styling design.

2.Authors need to add adequate problem definition (it doesn't specify what makes current methods inadequate). Also. research gap is implied rather than explicitly articulated. Author need to carefully write down research gaps which is unclear right now.

Response: Thanks for your suggestion.In the revised manuscript, we have clarified the problem definition and research gap in the Introduction section.

Specifically, we point out that existing studies on automotive product risk identification mainly focus on manufacturing and supply chain stages, while the styling design stage characterized by high uncertainty, multi-factor coupling, and strong subjectivity has received limited attention. We further summarize the limitations of existing methods, including the lack of structured and quantitative capabilities in traditional approaches, the strong subjectivity of AHP-based methods, the absence of ranking ability in WBS-RBS, and the inadequacy of fully compensatory methods in capturing non-compensatory critical risks. Accordingly, we explicitly identify the research gaps and note that no previous study has integrated an improved FAHP with VIKOR for risk identification in complete‑vehicle styling design tasks.

3.What authors have improved here is unclear. FAHP already existed in literature VIKOR is also existing technique. Whether author claiming integration as improvement or any mathematical changes, must be specified clearly.

Response: Thanks for your suggestion. we have clarified the main contribution in the Introduction sections.

Specifically, we emphasize that the proposed method introduces a mathematical improvement to the FAHP approach by constructing a fuzzy consistent matrix and solving it based on vector norm, which enables more accurate determination of risk factor weights. On this basis, the improved FAHP is further integrated with the VIKOR method to achieve more reliable risk ranking. These revisions explicitly highlight the methodological contribution and novelty of the proposed approach.

4.Authors are advised to add sensitivity analysis, which is mandatory for justifying the results.

Response: Thanks for your suggestion. we have added a sensitivity analysis in Section 6.2 to evaluate the robustness of the proposed method.

Specifically, taking the headlamp design task (W11) as an example, the results show that technical risk has the highest weight, followed by schedule risk, while cost risk has the lowest contribution, indicating that the overall risk is mainly driven by engineering feasibility and design complexity. Furthermore, the sensitivity analysis demonstrates that small variations in the weight of technical risk lead to significant changes in the overall risk ranking, whereas changes in cost risk have a relatively limited impact. These findings indicate that the proposed model is highly sensitive to critical risk factors and can effectively identify dominant risks, thereby validating the rationality of the weight distribution and the stability of the model. The weights of the five risk factors for other WBS nodes are provided in the supplementary material.

5.In addition, authors need to compare the results with other FAHP based methods (atleast 2-3) for better understanding of results.

Response: Thanks for your suggestion. we have added a comparative study in Section 5.4 to further validate the proposed method.

Specifically, we compare our improved FAHP‑VIKOR approach with three other methods using the same WBS‑RBS matrix and risk factor weights: (1) single FAHP weighted evaluation, (2) traditional FAHP‑TOPSIS, and (3) traditional FAHP‑VIKOR. The results show that single FAHP is sensitive to local extreme values and overestimates certain risks; the traditional FAHP‑TOPSIS still suffers from full compensation, which may mask critical non‑compensatory risks; and the traditional FAHP‑VIKOR is limited by the stability of weight determination. In contrast, our improved FAHP‑VIKOR method achieves more stable and reasonable risk rankings, better reflecting the non‑compensatory nature of critical risks in automotive styling design.

6.The introduction section seems weak and required to add qualitative literature in the context of MCDM methods in different applications as given below.

https://link.springer.com/article/10.1007/s12046-018-1020-x, https://link.springer.com/article/10.1007/s12633-020-00600-4, https://link.springer.com/article/10.1007/s12008-023-01528-w,

https://doi.org/10.1002/pc.26097,

https://journals.sagepub.com/doi/abs/10.1177/1464420720953447, https://www.nature.com/articles/s41598-025-16827-3, https://link.springer.com/article/10.1007/s12633-025-03374-9

Response: Thanks for your suggestion. we have strengthened the literature review in the Introduction section by incorporating additional studies on MCDM applications across different engineering domains.

Specifically, we have added representative works involving methods such as VIKOR, TOPSIS, MOORA, PROMETHEE, and their hybrid extensions (e.g., FAHP–VIKOR, FAHP–TOPSIS), covering applications in risk assessment, material selection, and infrastructure evaluation. we have cited two of the references you recommended: Yadav et al. [31] (TOPSIS-PSI for material selection) and Gangwar et al. [32] (AHP-MOORA for material optimization), which are now included in our discussion of fully compensatory MCDM methods and their limitations in risk-sensitive contexts, and several recent studies have also been added to further enhance the timeliness of the literature review.

7.How the authors have identified high risk nodes is unclear?

Response: Thanks for your suggestion. We have clarified the identification criteria for high-risk nodes in Section 4.2. Specifically, the risk classification standard is based on the VIKOR benefit ratio Q, referencing ISO 31000/ISO 31010 risk grading principles and LEC method interval division, combined with the risk distribution characteristics of automotive styling design tasks. The continuous evaluation results are divided into three levels: high risk (Q: 0-0.3), medium risk (Q: 0.3-0.7), and low risk (Q: 0.7-1.0). This grading enhances the interpretability of risk identification results and facilitates hierarchical decision-making and resource allocation in engineering management.

8.Authors must classify these high risk nodes in terms of design complexity, manufacturing constraints, or technological uncertainty (e.g., lidar integration) for better clarification.

Response: Thanks for your suggestion. we have classified the high- and medium-risk nodes in Section 5.3 according to their risk driving mechanisms.

Specifically, these nodes are categorized into three types: (1) high design complexity tasks (e.g., front and rear faces), where risks arise from the strong coupling between styling innovation and multiple engineering constraints; (2) engineering and manufacturing constraint-driven tasks (e.g., body structural parts), where risks mainly originate from conflicts between manufacturability and regulatory compliance; and (3) technological uncertainty-driven tasks (e.g., LiDAR surface), where risks are influenced by both new technology integration and regulatory changes. In addition, some medium-risk nodes exhibit notable cross-departmental coordination characteristics. The corresponding classification results are summarized in Table 8.

9.Authors need to add some implications of this work with challenges and future work.

Response: Thanks for your suggestion.

We have added a dedicated discussion on the implications, challenges, and future work in the Conclusion section. The main additions are as follows:

Implications: This study advances automotive styling risk management from manufacturing/supply-chain stages to the critical early design phase. The proposed WBS-RBS framework and improved FAHP-VIKOR method provide enterprises with an actionable tool for proactive risk control, enabling graded management strategies (e.g., time buffers for high-risk tasks, dynamic monitoring for medium-risk tasks) that directly support project scheduling and resource allocation.

Challenges: Three limitations are acknowledged: (1) reliance on expert scoring for risk probability and loss quantification, though mitigated through multi-round calibration; (2) static framework without capturing dynamic risk evolution across design stages; (3) need for further validation of model generalizability across different enterprise workflows and risk appetites.

Future work: We propose integrating risk quantification results with project schedule optimization, such as embedding risk weights into critical path methods or resource-constrained scheduling models. Additionally, dynamic scheduling mechanisms aligned with multi-stage design iterations will be explored to enable real-time task priority adjustment based on risk evolution.

10.The authors need to work highly on linguistic and grammatical errors throughout the manuscript. Best wishes

Response: Thanks for your suggestion. The manuscript has been thoroughly revised to improve language quality and readability. We have carefully checked grammar, terminology, and sentence structure throughout the paper to ensure clarity and professionalism.

Special thanks to you for your comments.

At last, we tried our best to improve the manuscript and made some changes in the manuscript. These changes will not influence the content and framework of the paper. And we marked them in yellow in the revised paper. Based on the reviewer comments, the supplementary files have been revised and re-uploaded.

We appreciate for Editor’s and Reviewers’ warm work earnestly and hope that the correction will meet with approval.

Once again, thank you very much for your comments and suggestions.

Yours sincerely,

Ganshu Sheng (Corresponding Author)

shenggs@hbut.edu.cn

Attachment Submitted filename: Responses to reviewer comments - PONE-D-25-67607R1.docx

# Peer review: Decision Letter 2

**DOI:** 10.1371/journal.pone.0352278.r005

**Authors:** Mithilesh K. Dikshit, Mithilesh K. Dikshit, Mithilesh K. Dikshit

8 Jun 2026

Risk Identification Method for Automotive Styling Design Tasks Based on an Improved FAHP-VIKOR Approach

PONE-D-25-67607R2

Dear Dr. Sheng,

We’re pleased to inform you that your manuscript has been judged scientifically suitable for publication and will be formally accepted for publication once it meets all outstanding technical requirements.

Within one week, you’ll receive an e-mail detailing the required amendments. When these have been addressed, you’ll receive a formal acceptance letter and your manuscript will be scheduled for publication.

An invoice will be generated when your article is formally accepted. Please note, if your institution has a publishing partnership with PLOS and your article meets the relevant criteria, all or part of your publication costs will be covered. Please make sure your user information is up-to-date by logging into Editorial Manager at Editorial Manager® and clicking the ‘Update My Information' link at the top of the page. For questions related to billing, please contact billing support.

If your institution or institutions have a press office, please notify them about your upcoming paper to help maximize its impact. If they’ll be preparing press materials, please inform our press team as soon as possible -- no later than 48 hours after receiving the formal acceptance. Your manuscript will remain under strict press embargo until 2 pm Eastern Time on the date of publication. For more information, please contact onepress@plos.org.

Kind regards,

Mithilesh K. Dikshit

Academic Editor

PLOS One

Additional Editor Comments (optional):

The manuscript is accepted in the present form.

Reviewers' comments:

Reviewer's Responses to Questions

**Comments to the Author**

1. If the authors have adequately addressed your comments raised in a previous round of review and you feel that this manuscript is now acceptable for publication, you may indicate that here to bypass the “Comments to the Author” section, enter your conflict of interest statement in the “Confidential to Editor” section, and submit your "Accept" recommendation.

Reviewer #3: All comments have been addressed

**********

2. Is the manuscript technically sound, and do the data support the conclusions?

The manuscript must describe a technically sound piece of scientific research with data that supports the conclusions. Experiments must have been conducted rigorously, with appropriate controls, replication, and sample sizes. The conclusions must be drawn appropriately based on the data presented.

Reviewer #3: Yes

**********

3. Has the statistical analysis been performed appropriately and rigorously?

Reviewer #3: Yes

**********

4. Have the authors made all data underlying the findings in their manuscript fully available?

The PLOS Data policy requires authors to make all data underlying the findings described in their manuscript fully available without restriction, with rare exception (please refer to the Data Availability Statement in the manuscript PDF file). The data should be provided as part of the manuscript or its supporting information, or deposited to a public repository. For example, in addition to summary statistics, the data points behind means, medians and variance measures should be available. If there are restrictions on publicly sharing data—e.g. participant privacy or use of data from a third party—those must be specified.

Reviewer #3: Yes

**********

5. Is the manuscript presented in an intelligible fashion and written in standard English?

PLOS ONE does not copyedit accepted manuscripts, so the language in submitted articles must be clear, correct, and unambiguous. Any typographical or grammatical errors should be corrected at revision, so please note any specific errors here.

Reviewer #3: Yes

**********

6. Review Comments to the Author

Please use the space provided to explain your answers to the questions above. You may also include additional comments for the author, including concerns about dual publication, research ethics, or publication ethics. (Please upload your review as an attachment if it exceeds 20,000 characters)

Reviewer #3: All the comments have been addressed, which improves the quality of manuscript significantly. Thus, can be accepted in its current form.

**********

7. PLOS authors have the option to publish the peer review history of their article (what does this mean?). If published, this will include your full peer review and any attached files.

If you choose “no”, your identity will remain anonymous but your review may still be made public.

**Do you want your identity to be public for this peer review?**  For information about this choice, including consent withdrawal, please see our Privacy Policy.

Reviewer #3: **Yes:**  Vimal Kumar Pathak

**********

# Editor report: Acceptance letter

**DOI:** 10.1371/journal.pone.0352278.r006

**Authors:** Mithilesh K. Dikshit, Mithilesh K. Dikshit, Mithilesh K. Dikshit

PONE-D-25-67607R2

PLOS One

Dear Dr. Sheng,

I'm pleased to inform you that your manuscript has been deemed suitable for publication in PLOS One. Congratulations! Your manuscript is now being handed over to our production team.

At this stage, our production department will prepare your paper for publication. This includes ensuring the following:

* All references, tables, and figures are properly cited

* All relevant supporting information is included in the manuscript submission,

* There are no issues that prevent the paper from being properly typeset

You will receive further instructions from the production team, including instructions on how to review your proof when it is ready. Please keep in mind that we are working through a large volume of accepted articles, so please give us a few days to review your paper and let you know the next and final steps.

Lastly, if your institution or institutions have a press office, please let them know about your upcoming paper now to help maximize its impact. If they'll be preparing press materials, please inform our press team within the next 48 hours. Your manuscript will remain under strict press embargo until 2 pm Eastern Time on the date of publication. For more information, please contact onepress@plos.org.

You will receive an invoice from PLOS for your publication fee after your manuscript has reached the completed accept phase. If you receive an email requesting payment before acceptance or for any other service, this may be a phishing scheme. Learn how to identify phishing emails and protect your accounts at https://explore.plos.org/phishing.

If we can help with anything else, please email us at customercare@plos.org.

Thank you for submitting your work to PLOS ONE and supporting open access.

Kind regards,

PLOS ONE Editorial Office Staff

on behalf of

Dr. Mithilesh K. Dikshit

Academic Editor

PLOS One
